# Impact of Sample Type and DNA Isolation Procedure on Genomic Inference of Microbiome Composition

**DOI:** 10.1128/mSystems.00095-16

**Published:** 2016-10-18

**Authors:** Berith E. Knudsen, Lasse Bergmark, Patrick Munk, Oksana Lukjancenko, Anders Priemé, Frank M. Aarestrup, Sünje J. Pamp

**Affiliations:** a National Food Institute, Technical University of Denmark, Kgs. Lyngby, Denmark; b Department of Biology, University of Copenhagen, Copenhagen, Denmark; Pacific Northwest National Laboratory

**Keywords:** 16S rRNA gene profiling, DNA isolation, metagenomics, microbial ecology, microbiome, next-generation sequencing

## Abstract

Explorations of complex microbiomes using genomics greatly enhance our understanding about their diversity, biogeography, and function. The isolation of DNA from microbiome specimens is a key prerequisite for such examinations, but challenges remain in obtaining sufficient DNA quantities required for certain sequencing approaches, achieving accurate genomic inference of microbiome composition, and facilitating comparability of findings across specimen types and sequencing projects. These aspects are particularly relevant for the genomics-based global surveillance of infectious agents and antimicrobial resistance from different reservoirs. Here, we compare in a stepwise approach a total of eight commercially available DNA extraction kits and 16 procedures based on these for three specimen types (human feces, pig feces, and hospital sewage). We assess DNA extraction using spike-in controls and different types of beads for bead beating, facilitating cell lysis. We evaluate DNA concentration, purity, and stability and microbial community composition using 16S rRNA gene sequencing and for selected samples using shotgun metagenomic sequencing. Our results suggest that inferred community composition was dependent on inherent specimen properties as well as DNA extraction method. We further show that bead beating or enzymatic treatment can increase the extraction of DNA from Gram-positive bacteria. Final DNA quantities could be increased by isolating DNA from a larger volume of cell lysate than that in standard protocols. Based on this insight, we designed an improved DNA isolation procedure optimized for microbiome genomics that can be used for the three examined specimen types and potentially also for other biological specimens. A standard operating procedure is available from https://dx.doi.org/10.6084/m9.figshare.3475406.

**IMPORTANCE** Sequencing-based analyses of microbiomes may lead to a breakthrough in our understanding of the microbial worlds associated with humans, animals, and the environment. Such insight could further the development of innovative ecosystem management approaches for the protection of our natural resources and the design of more effective and sustainable solutions to prevent and control infectious diseases. Genome sequence information is an organism (pathogen)-independent language that can be used across sectors, space, and time. Harmonized standards, protocols, and workflows for sample processing and analysis can facilitate the generation of such actionable information. In this study, we assessed several procedures for the isolation of DNA for next-generation sequencing. Our study highlights several important aspects to consider in the design and conduct of sequence-based analysis of microbiomes. We provide a standard operating procedure for the isolation of DNA from a range of biological specimens particularly relevant in clinical diagnostics and epidemiology.

## Abstract (precis)

Sequencing-based analyses of microbiomes may lead to a breakthrough in our understanding of the microbial worlds associated with humans, animals, and the environment. Such insight could further the development of innovative ecosystem management approaches for the protection of our natural resources and the design of more effective and sustainable solutions to prevent and control infectious diseases. Genome sequence information is an organism (pathogen)-independent language that can be used across sectors, space, and time. Harmonized standards, protocols, and workflows for sample processing and analysis can facilitate the generation of such actionable information. In this study, we assessed several procedures for the isolation of DNA for next-generation sequencing. Our study highlights several important aspects to consider in the design and conduct of sequence-based analysis of microbiomes. We provide a standard operating procedure for the isolation of DNA from a range of biological specimens particularly relevant in clinical diagnostics and epidemiology.

## INTRODUCTION

Microbial communities fulfill central roles in biological systems, such as in human, animal, and environmental ecosystems. Genomics-based interrogations of these communities can provide unprecedented insight into their composition and function and reveal general principles and rules about their ecology and evolution (1–4).

Genomics-based microbiome analyses can also have important practical implications, such as for the diagnosis and management of infectious diseases. Together with relevant metadata, attribute data, and appropriate bioinformatics and statistical approaches, genomic sequencing data could enable the global surveillance of emerging and reemerging infectious diseases and teach us about the reservoirs and transmission pathways of pathogens (5–7). Ultimately, genomics-based information about infectious disease epidemiology may help us to predict, prevent, and control infectious diseases faster, more precisely, and more sustainably.

In order to facilitate large-scale microbiome analyses, harmonized standards for sample handling and data analysis need to be ensured. To be able to establish pathogen reservoirs and transmission pathways, specimens from different sources, such as from humans, animals, and the environment, will need to be examined. For genomics analysis, the DNA needs to be isolated from the specimens for DNA sequencing. However, DNA isolation methods are often evaluated and established only in the context of specimens from an individual source (e.g., human fecal specimens) and seldom across a variety of specimen types (8–12), which is addressed in the present study.

Current sequencing technologies, such as Illumina MiSeq and HiSeq, PacBio, Ion Torrent, and nanopore systems, still require large initial DNA template quantities, particularly from the perspective of PCR-free metagenomics-based analysis. In contrast, 16S rRNA gene profiling can reveal a bacterial and archaeal composition for samples with low initial DNA template quantities. In metagenomics, low quantities of input DNA can result in low sequencing data output and impact the inferred microbial community composition (13). Hence, modified DNA isolation protocols for increasing DNA quantities obtained from different types of specimens are desirable.

Here, we examine three specimen types (human feces, animal feces, and sewage), a total of eight commercially available DNA isolation kits, and a number of protocol modifications in regard to output DNA (quantity, purity, and stability) and microbiome composition (16S rRNA gene profiling and metagenomics). Our results suggest that both the specimen itself and the DNA isolation procedure can affect DNA quantity and quality and inferred microbiome composition. Based on the insight gained, we have developed an improved laboratory protocol that can be used for DNA isolations from a variety of biological specimens.

## RESULTS

### DNA concentration, purity, and stability depend on the type of specimen and DNA isolation method.

**FIG 1:**
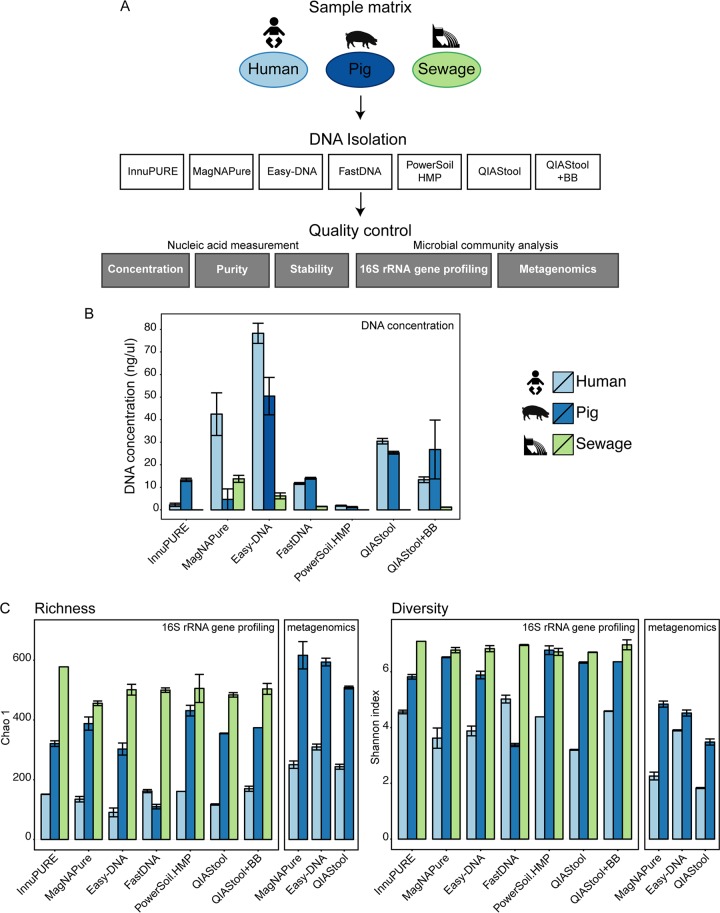
Comparison of DNA extraction methods. (A) Experimental design. Human feces, pig feces, and hospital sewage were extracted using seven different DNA extraction methods (Table 1): InnuPure C16, MagNA Pure LC DNA isolation kit III, Easy-DNA gDNA purification kit, MP FastDNA Spin kit, PowerSoil DNA isolation kit, QIAamp DNA stool minikit, and QIAamp DNA stool minikit plus bead beating (for details, see Materials and Methods). DNA concentration, purity, and stability were examined, and microbial community composition was determined using 16S rRNA gene profiling and metagenomics (selected samples). (B) DNA from each method was dissolved in 100 µl solution, and DNA concentrations were determined using Qubit dsDNA BR assay kit measurements. Values represent averages from duplicate or triplicate DNA extractions (see also Table S1A in the supplemental material). (C) Ecological richness (Chao 1) and diversity (Shannon index) were determined based on contingency tables from 16S rRNA gene profiling and metagenomic sequencing data at OTU and species levels, respectively (see also Table S1B).

We extracted DNA from human feces, pig feces, and hospital sewage, using seven commonly used DNA isolation kits, and determined DNA concentration, purity, and stability of the isolated DNA (Fig. 1A; Table 1). The DNA concentrations varied greatly (Fig. 1B; see also Table S1A in the supplemental material). For human feces, the highest DNA concentrations were obtained using the Easy-DNA, MagNA Pure, and QIAamp DNA stool minikit (QIAStool) procedures; for pig feces, the highest concentrations were obtained using the Easy-DNA, QIAStool, and QIAStool plus bead beating (QIAStool+BB) procedures; and for sewage, the highest concentrations were obtained using the MagNA Pure and Easy-DNA procedures, while for three methods the DNA concentration from sewage was below the detection limit. On average across the three types of specimen, the highest DNA concentrations were obtained using Easy-DNA (44.96 ± 20.99 [standard error of the mean {SEM}] ng/µl) and QIAStool (27.88 ± 2.55 [SEM] ng/µl), and the lowest were obtained using the PowerSoil.HMP (1.55 ± 0.31 [SEM] ng/µl) and InnuPure (7.77 ± 5.54 [SEM] ng/µl) methods.

10.1128/mSystems.00095-16.7 Table S1  Comparison of DNA extraction methods. (A) DNA concentration, purity, and stability; (B) microbiome richness and diversity. Download Table S1, XLSX file, 0.5 MB. Copyright © 2016 Knudsen et al. 2016 Knudsen et al. This content is distributed under the terms of the Creative Commons Attribution 4.0 International license.

**TABLE 1:** Overview of DNA extraction procedures

Extraction method^d^	Sample amount (g)	Cell lysis methods	Bead type	DNA separation	Cost per extraction (€)^a^	Processing time for 20 samples (h)
Step 1: seven commonly used DNA extraction kits						
InnuPure C16 (Analytic Jena AG) [A]	0.1	Chemical, mechanical, heat	Ceramic	Magnetic beads	7.3	4
MagNA Pure LC DNA isolation kit III (Roche) [A]	0.25	Chemical, heat		Magnetic beads	2.6^b^	2.5
Easy-DNA gDNA purification kit (Invitrogen)	0.25	Chemical, enzymatic	None	Phenol-chloroform precipitation	4.5	8.8
MP FastDNA Spin kit (MP Biomedicals)	0.5	Chemical, mechanical	Ceramic and garnet	Silica membrane- based columns	14.1^c^	5
PowerSoil DNA isolation kit (MoBio)	0.25	Chemical, mechanical, heat	Garnet	Silica membrane- based columns	5.3	5.5
QIAamp DNA stool minikit (Qiagen)	0.2	Chemical, heat		Silica membrane- based columns	5.3	4
QIAamp DNA stool minikit (Qiagen) + BB (lysing matrix A; MP Biomedicals)	0.2	Chemical, mechanical, heat	Ceramic and garnet	Silica membrane- based columns	12.7	4
Step 2: new DNA extraction kit and modified DNA extraction procedures						
QIAamp DNA stool minikit (Qiagen) + BB (garnet bead tubes; MoBio)	0.2	Chemical, mechanical, heat	Garnet	Silica membrane- based columns	8.5	3
QIAamp Fast DNA stool minikit	0.2	Chemical, mechanical, heat		Silica membrane- based columns	6.2	2.6
QIAamp Fast DNA stool minikit + BB (lysing matrix A; MP Biomedicals)	0.2	Chemical, mechanical, heat	Ceramic and garnet	Silica membrane- based columns	13.6	3
QIAamp Fast DNA stool minikit + BB (pathogen lysis tubes S; Qiagen)	0.2	Chemical, mechanical, heat	Glass	Silica membrane- based columns	10	3
QIAamp Fast DNA stool minikit + BB (pathogen lysis tubes L; Qiagen)	0.2	Chemical, mechanical, heat	Glass	Silica membrane- based columns	10	3
QIAamp Fast DNA stool minikit + BB (garnet bead tubes; MoBio)	0.2	Chemical, mechanical, heat	Garnet	Silica membrane- based columns	8.5	3
QIAamp Fast DNA stool minikit + BB (bead beating tubes; A&A Biotechnology)	0.2	Chemical, mechanical, heat	Zirconia-silica	Silica membrane- based columns	8.2	3

^a^ Calculations do not include costs for additional laboratory supplies, such as pipette tips and reaction tubes.

^b^ Excluding costs for special pipette tips and plastic cartridges required for the robot.

^c^ Based on price in the United States, excluding general sales tax that is added in other countries.

^d^ Abbreviations: [A], automated procedure; BB, bead beating.

With regard to DNA purity, the best results for human and pig feces were obtained using the Easy-DNA, QIAStool, and QIAStool+BB procedures (see Table S1A in the supplemental material). The DNA was generally stable for at least 7 days when stored at room temperature (22°C) with some exceptions (see Table S1A in the supplemental material). A decrease in DNA concentration over time was observed, for example for the human feces when extracted with Easy-DNA (57% decrease in DNA concentration) or MagNA Pure (21% decrease in DNA concentration), suggesting the presence of DNases in these extracts. In some cases, an increase in DNA concentration over time was observed, such as for the pig feces when extracted with Easy-DNA (32% increase in DNA concentration). An increase in DNA concentration over time at room temperature was previously shown to be related to the hyperchromicity of DNA and dependent on the DNA concentration and ionic strength of the solution (14).

### Microbial richness and diversity are influenced by DNA isolation procedure.

For the human fecal specimen, the highest bacterial operational taxonomic unit (OTU) richness and diversity were detected using the QIAStool+BB and FastDNA methods, followed by InnuPure and PowerSoil.HMP as assessed by 16S rRNA gene profiling (Fig. 1C; see also Table S1B in the supplemental material). In comparison, the determined richness and diversity for the Easy-DNA method were low, and the relative abundance of *Ruminococcaceae* and *Bifidobacteriaceae* dominated the composition compared to the extracts from the other methods (Fig. 1C; see also Fig. S1A in the supplemental material). Thirty-nine samples (human feces, pig feces, and sewage) with high DNA concentrations were selected and examined using metagenomic sequencing. In this assessment, the species richness and diversity for human feces were highest for the Easy-DNA procedure, and a high relative abundance of *Ruminococcaceae* and *Bifidobacteriaceae* was apparent in this analysis as well (see Fig. S1A in the supplemental material).

10.1128/mSystems.00095-16.2 Figure S1  Microbial community composition. The 10 most abundant families for the human fecal (A), pig fecal (B), and hospital sewage (C) samples based on (i) 16S rRNA gene profiling, (ii) metagenomics analysis that includes normalization based on reference genome size, and (iii) metagenomics analysis without normalization according to genome size. For details regarding sequence data analysis and normalization, see Materials and Methods. Download Figure S1, TIF file, 0.7 MB. Copyright © 2016 Knudsen et al. 2016 Knudsen et al. This content is distributed under the terms of the Creative Commons Attribution 4.0 International license.

For the pig fecal specimen, the highest bacterial richness and diversity were detected using the PowerSoil.HMP and MagNA Pure methods, followed by QIAStool+BB (Fig. 1C; see also Table S1B in the supplemental material). Similarly, richness and diversity were highest using the MagNA Pure and Easy-DNA methods when assessed using metagenomics. Based on 16S rRNA gene profiling, the richness and diversity for the FastDNA method were lower than those for all other methods, and the relative abundance of *Clostridiaceae* and *Turicibacteraceae* was higher and the abundance of *Prevotellaceae* and *Ruminococcaceae* was lower using this method than using the other methods (Fig. 1C; see also Fig. S1A in the supplemental material).

For the sewage specimen, the highest bacterial richness and diversity were detected using the InnuPure method, followed by PowerSoil.HMP and QIAStool+BB, and similar levels were achieved using the other methods (Fig. 1C; see also Table S1B in the supplemental material). The relative abundance of *Clostridiaceae* was highest in the samples extracted using Easy-DNA, and the abundance of *Enterobacteriales* was highest in the samples extracted using PowerSoil.HMP.

Overall, the relative abundance of predicted Gram-positive bacteria was highest in the human and sewage specimens when extracted with the Easy-DNA method and highest in the pig specimen when extracted using the FastDNA method (see Fig. S2 in the supplemental material). The abundance of predicted Gram-positive bacteria was lowest using MagNA Pure and QIAStool, the two methods that included neither a bead beating step nor specific enzymatic cell wall digestion.

10.1128/mSystems.00095-16.3 Figure S2  Microbial community composition based on predicted Gram staining. Gram-positive and Gram-negative affiliations were assigned at the order level based on information found in the literature. For some taxa, the Gram staining status was unknown. Download Figure S2, TIF file, 0.6 MB. Copyright © 2016 Knudsen et al. 2016 Knudsen et al. This content is distributed under the terms of the Creative Commons Attribution 4.0 International license.

### Microbial community composition depends on the choice of DNA isolation procedure.

**FIG 2:**
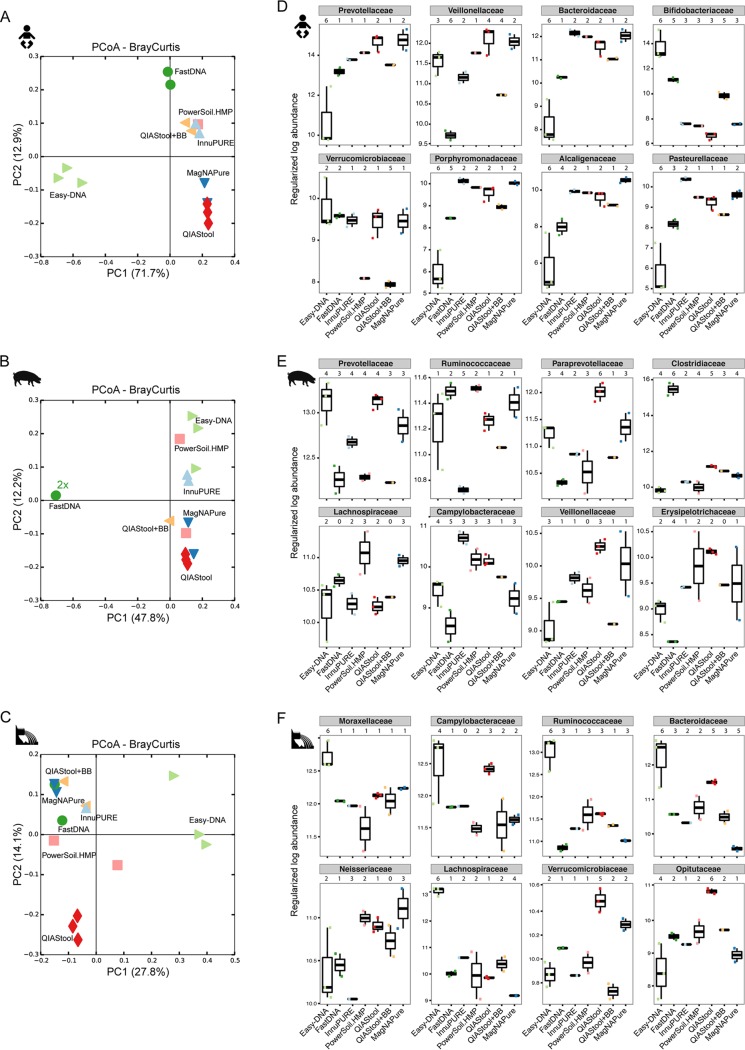
Microbial community dissimilarity. The dissimilarity between the microbiotas from the human, pig, and sewage samples based on DNA extraction methods was examined using principal-coordinate analysis of Bray-Curtis distances (A to C) and differential abundance analysis using DESeq2 (D to F) from 16S rRNA amplicon data. (A to C) For the PCoA Bray-Curtis ordination analysis, only samples with 800 or more reads were included. (D to F) For the differential abundance analysis, pairwise testing by the DNA extraction method was performed, and bacterial families were considered significantly differentially abundant if their adjusted *P* value was <0.1 (see also Table S2 in the supplemental material). Examples for differentially abundant families are shown that are among the 10 most abundant taxa found in the sample. For each family, the total number of DNA isolation procedures that exhibit significantly different abundance values compared to a particular DNA isolation procedure is indicated above the plot. Easy-DNA, light green; FastDNA, dark green; InnuPURE, light blue; PowerSoil.HMP, light red; QIAStool, red; QIAStool+BB, orange; MagNAPure, blue.

The microbial communities from the three types of specimen clustered separately according to specimen type when examined in principal-coordinate analysis (PCoA) Bray-Curtis ordination and not according to DNA isolation procedure (see Fig. S3 in the supplemental material), indicating that the largest differences between these samples are driven by the inherent microbiota composition. Bray-Curtis dissimilarity distance analysis carried out separately for each of the three specimens revealed that the samples largely clustered according to DNA isolation procedure (Fig. 2A to C). For the human fecal specimen, the bacterial community composition derived from the Easy-DNA isolation differed from the communities obtained using all other methods (Fig. 2A), which is in agreement with the observations on microbial richness (above). The Bray-Curtis distances between the samples from InnuPure, MagNA Pure, FastDNA, PowerSoil.HMP, QIAStool, and QIAStool+BB DNA isolations were on average 0.337 ± 0.012 (SEM), whereas the distances between these and the ones derived from the Easy-DNA procedure were on average 0.825 ± 0.014 (SEM).

10.1128/mSystems.00095-16.4 Figure S3  Microbial community dissimilarity. The dissimilarity between the microbiotas from the human, pig, and sewage samples was examined using principal-coordinate analysis of Bray-Curtis distances based on the 16S rRNA gene count data. For the PCoA Bray-Curtis ordination analysis, only samples with a minimum of 800 reads were included. Additional results regarding community dissimilarity (based on Bray-Curtis) and similarity (based on Jaccard similarity coefficient) within and between DNA extraction procedures across sample types as well as for a given sample type are available through Figshare at https://dx.doi.org/10.6084/m9.figshare.3814239. Download Figure S3, TIF file, 0.1 MB. Copyright © 2016 Knudsen et al. 2016 Knudsen et al. This content is distributed under the terms of the Creative Commons Attribution 4.0 International license.

10.1128/mSystems.00095-16.8 Table S2  Differential abundance of families. (A) human fecal microbial community; (B) pig fecal microbial community; (C) hospital sewage microbial community. Download Table S2, PDF file, 0.3 MB. Copyright © 2016 Knudsen et al. 2016 Knudsen et al. This content is distributed under the terms of the Creative Commons Attribution 4.0 International license.

For the pig fecal specimen, the bacterial communities derived from the FastDNA isolation differed from all other communities (Fig. 2B). The average Bray-Curtis distance between the samples originating from all but the FastDNA procedure was on average 0.473 ± 0.008 (SEM), whereas the distance between these and the ones derived from the FastDNA procedure was on average 0.877 ± 0.007 (SEM).

For the hospital sewage specimen, the bacterial communities originating from the Easy-DNA method differed from all others (average Bray-Curtis distance, 0.600 ± 0.006 [SEM]) (Fig. 2C), similar to the human fecal matrix (Fig. 2A). In addition, the communities originating from the QIAStool DNA isolation differed from all others (average Bray-Curtis distance, 0.514 ± 0.009 [SEM]), whereas the average Bray-Curtis distance between all but the QIAStool and Easy-DNA samples was 0.460 ± 0.11 (SEM).

### Distinct taxa account for the differences observed between DNA isolation methods.

To quantify the effect of DNA isolation method on microbial community composition, we tested for differential abundance of taxa between the communities derived from the different DNA isolation methods using DESeq2 analyses. In pairwise comparisons, significant differences between the DNA isolation methods were observed (Fig. 2D to F; see also Table S2 in the supplemental material).

The most abundant family on average in the human fecal specimen was *Prevotellaceae* (*Bacteroidetes*), and its abundance was significantly lower in the samples extracted with Easy-DNA than in samples with all other methods (e.g., 18.3-fold lower in Easy-DNA than in QIAStool; adjusted *P* value, 1.91^−6^) (Fig. 2D; see also Table S2 in the supplemental material). Similarly, the abundance of *Bacteroidaceae* (*Bacteroidetes*), *Porphyromonadaceae* (*Bacteroidetes*), *Alcaligenaceae* (*Betaproteobacteria*), and *Pasteurellaceae* (*Gammaproteobacteria*) was lower in the samples from the Easy-DNA isolation than in samples from the other methods. In contrast, the abundance of *Bifidobacteriaceae* (*Actinobacteria*) was higher in the samples originating from the Easy-DNA procedure than in samples from all other methods (e.g., 770-fold higher in Easy-DNA than in QIAStool; adjusted *P* value, 7.49^−57^). The abundance of *Verrucomicrobiaceae* (*Verrucomicrobia*) was significantly lower in the samples from the QIAStool+BB and PowerSoil.HMP DNA isolations (e.g., 4.15-fold lower in QIAStool+BB than in QIAStool; adjusted *P* value, 0.001).

The most abundant family on average in the pig fecal specimen was *Prevotellaceae* (*Bacteroidetes*), and its abundance differed significantly between the DNA isolation procedures (e.g., 2.3-fold lower in Easy-DNA than in PowerSoil.HMP; adjusted *P* value, 1.28^−5^) (Fig. 2E; see also Table S2 in the supplemental material). The abundance of *Clostridiaceae* (*Clostridia*), the fourth most abundant family in the pig feces on average, was significantly higher in the samples extracted by the FastDNA method (e.g., 166-fold higher in FastDNA than in Easy-DNA; adjusted *P* value, 7.35^−110^).

*Moraxellaceae* (*Gammaproteobacteria*) was the most abundant family on average in the hospital sewage, and its abundance was significantly higher in the samples from the Easy-DNA isolation than in samples from other DNA isolation methods (e.g., 2.6-fold higher in Easy-DNA than in PowerSoil.HMP; adjusted *P* value, 3.82^−5^) (Fig. 2F; see also Table S2 in the supplemental material). *Ruminococcaceae* (*Clostridia*), the third most abundant family in sewage on average, was also significantly more abundant in the samples from the Easy-DNA isolation than in samples from other DNA isolation procedures (e.g., 7.3-fold higher in Easy-DNA than in FastDNA; adjusted *P* value, 4.28^−17^).

### DNA isolation procedure affects the abundance of taxa differently across specimens.

**FIG 3:**
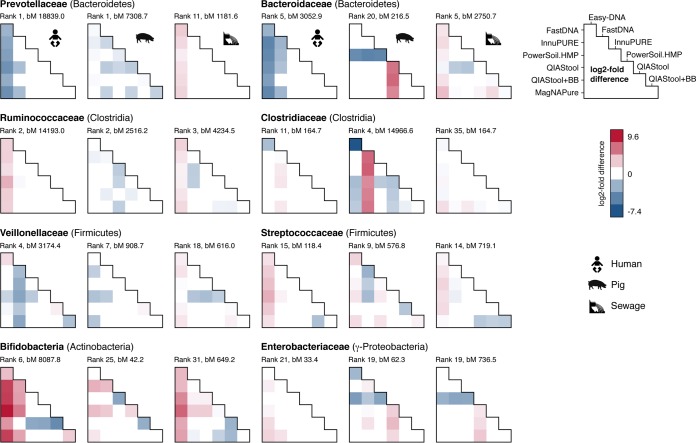
Differential abundance of bacterial families. Pairwise testing by the DNA extraction method was performed using DESeq2, and the log_2_ fold difference was displayed (column versus rows) for selected families present in all sample matrices if their adjusted *P* value was <0.1 (see also Table S2 in the supplemental material). The rank abundance position for each family per sample matrix type is noted according to their regularized log abundance. The baseMean (bM) indicates the mean of negative-binomial-based normalized read counts. The pairwise comparisons based on relative abundance normalization (total-sum scaling) of the bacterial families for the different DNA isolation procedures and three sample types are available through Figshare at https://dx.doi.org/10.6084/m9.figshare.3811254.

Given that differential taxon abundances were observed for the different DNA isolation procedures for the three specimen types, we investigated whether the abundance differed in the same way between DNA isolation procedures across specimens. For example, we were asking the following: if taxon A is observed at a higher abundance upon DNA isolation with method X than with method Y in specimen type 1, is this taxon also observed at a higher abundance upon DNA isolation with method X than with method Y in specimen type 2? We examined taxa that were detected in all three specimen types and selected representative families from different phyla (Fig. 3).

Similar patterns of differential abundance were observed for certain taxa across specimen types, with exceptions, including two families from the *Bacteroidetes* phylum. The abundance of *Prevotellaceae* and *Bacteroidaceae* was significantly lower when human fecal specimens were extracted with Easy-DNA than with other methods. In contrast, these two families were observed at a significantly higher abundance when sewage was extracted with Easy-DNA than with other methods (Fig. 3).

Likewise, *Ruminococcaceae* of the phylum *Clostridia* were observed at a significantly higher abundance in human fecal and hospital sewage samples but not in pig fecal samples when extracted with the Easy-DNA method than with other methods. The same pattern was, however, not observed for all families of the phylum *Clostridia*. *Clostridiaceae* abundance appeared higher in human and pig feces when extracted with FastDNA than with other methods, and *Clostridiaceae* abundance appeared higher in sewage when extracted using the Easy-DNA method than with other methods (Fig. 3).

Thus, we found significant differences in the abundances of certain families according to specimen type, which sometimes depended on the DNA isolation procedure. Some of the differential abundance patterns were similar across the three types of specimens, while others differed.

### Detection of spiked bacteria is dependent on DNA isolation procedure and specimen type.

In order to quantify DNA isolation efficiency, we spiked the three specimen with known numbers of two bacterial representatives, namely, *Salmonella enterica* serotype Typhimurium DT104 (Gram negative) and *Staphylococcus aureus* ST398 (Gram positive) in a CFU ratio of 1.02. Both *S. enterica* and *S. aureus* were present in negligible numbers in the three specimens before spiking. DNA was isolated from these samples using the seven different DNA isolation methods, and the abundance of the two strains was determined using 16S rRNA gene profiling and for some samples also using metagenomics. Based on 16S rRNA gene profiling, the spiked organisms accounted for an average abundance of 1.0% (±0.29% [SEM]) *Enterobacteriaceae* and 0.29% (±0.11% [SEM]) *Staphylococcaceae* across the three types of specimen.

**FIG 4:**
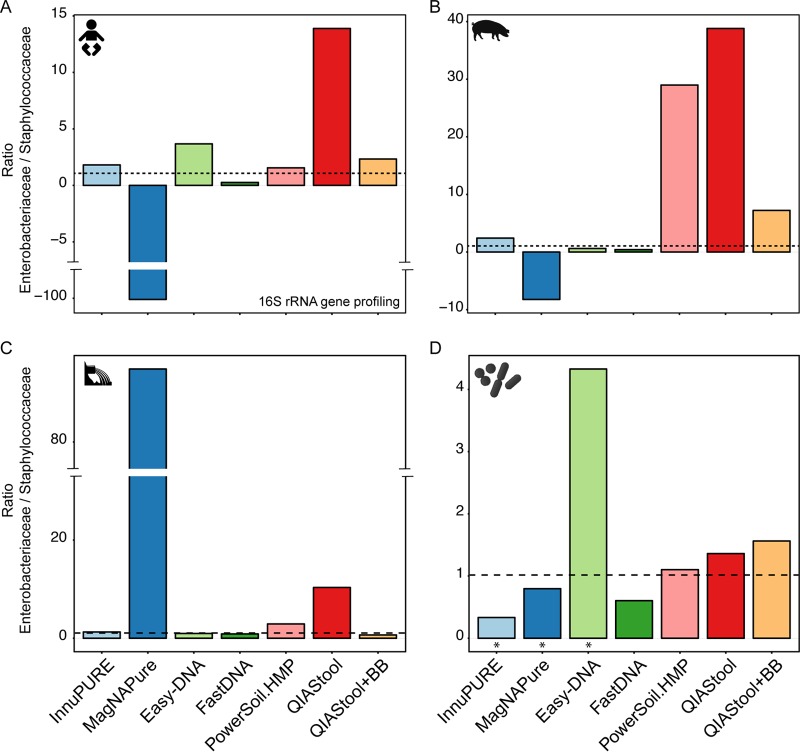
Detection of spiked bacteria. The human fecal (A), pig fecal (B), and hospital sewage (C) samples were spiked with a strain mix composed of *Salmonella enterica* serotype Typhimurium DT104 and *Staphylococcus aureus* ST398 in a CFU ratio of 1.02. The three sample matrices, as well as aliquots of the strain mix (D), were extracted using seven different DNA extraction methods. The two strains were detected by 16S rRNA gene profiling, and their ratios were determined. For details, see Materials and Methods. An asterisk in panel D indicates that the values for the particular DNA extraction of the strain mix are based on single measurements. All other values are based on averages from duplicate or triplicate DNA extractions. The dashed line indicates the ratio of the strain mix based on CFU determinations. The *x* axis scale is the same for all panels (A to D), and the *y* axis scale is specific for each sample type.

Using QIAStool, a DNA isolation method that does not involve a bead beating step, the abundance of *Enterobacteriaceae* was higher in the spiked human fecal specimen than expected, with an *Enterobacteriaceae*/*Staphylococcaceae* ratio of 13.9 (Fig. 4A). This ratio was lower in the spiked human fecal specimen using InnuPure, FastDNA, PowerSoil.HMP, and QIAStool+BB, which are all methods that involve a bead beating step (*Enterobacteriaceae*/*Staphylococcaceae* ratio range, 0.3 to 2.3). The Easy-DNA method involves an additional enzymatic lysis step, and using this method, the determined *Enterobacteriaceae*/*Staphylococcaceae* ratio was 3.7. Using the MagNA Pure method, no or lower read numbers assigned to *Staphylococcaceae* were detected in the spiked samples than in nonspiked samples in the human fecal specimen, and hence, the ratio resulted in negative values (Fig. 4A). A similar result was obtained when the samples were examined using metagenomics (see Fig. S4 in the supplemental material).

10.1128/mSystems.00095-16.5 Figure S4  Detection of spiked bacteria using metagenomics. The human fecal (A) and pig fecal (B) samples were spiked with a strain mix composed of *Salmonella enterica* serotype Typhimurium DT104 and *Staphylococcus aureus* ST398 in a CFU ratio of 1.02. These two sample matrices, as well as aliquots of the strain mix (C), were extracted using three different DNA extraction methods. The two strains were detected by metagenomics analysis, and their ratios were determined. For details, see Materials and Methods. An asterisk indicates that the values for the particular DNA extraction of the strain mix (D) are based on single measurements. All other values are based on averages from duplicate or triplicate measurements. The dashed line indicates the ratio of the strain mix based on CFU determinations. Download Figure S4, TIF file, 0.3 MB. Copyright © 2016 Knudsen et al. 2016 Knudsen et al. This content is distributed under the terms of the Creative Commons Attribution 4.0 International license.

Overall, most DNA isolation methods exhibited similar tendencies across the three types of specimen. For example, for all three specimen types, the *Enterobacteriaceae*/*Staphylococcaceae* ratio was higher using the QIAStool method than using the other methods (except MagNA Pure for sewage). However, when the strain mix, composed of *S. enterica* and *S. aureus* only, was extracted using the seven DNA isolation procedures, their determined *Enterobacteriaceae*/*Staphylococcaceae* ratio was in almost all cases similar to the expected ratio of 1.02, including the QIAStool method.

### Protocol modifications for increasing DNA concentration.

One goal in genomics is to obtain a predicted pattern of microbial community composition that closely resembles the actual composition of microorganisms in a particular environment. Another challenge is to obtain sufficient DNA for metagenome sequencing. To address this aspect, we examined the effect of modifications to standard protocols on output DNA concentration (modifications are described in detail in the supplemental materials and methods [see Text S1 in the supplemental material]). We chose the QIAStool method as a starting point, as we obtained DNA extracts using this method that were of high purity and stability (see Table S1A in the supplemental material). Another concern is processing time and costs for DNA isolation procedures, particularly for large-scale microbiome projects. The protocol of the QIAamp Fast DNA stool minikit (QIAFast), a kit that became available at the time that the present study was carried out, suggested reduced processing time compared to the QIAStool method. When we compared the QIAStool and QIAFast methods using metagenomic sequencing, we obtained similar richness, diversity, and microbial community composition with these two methods (see Fig. S5 in the supplemental material).

10.1128/mSystems.00095-16.1 Text S1  Supplemental materials and methods. Details regarding specimen collection and handling, spiking with the strain mix, DNA isolation, DNA quantitation and quality assessment, 16S rRNA gene profiling, metagenomics, differential abundance analysis, and quantification of the strain mix are described. Download Text S1, PDF file, 0.2 MB. Copyright © 2016 Knudsen et al. 2016 Knudsen et al. This content is distributed under the terms of the Creative Commons Attribution 4.0 International license.

10.1128/mSystems.00095-16.6 Figure S5  Comparison between QIAStool and QIAFast DNA extraction methods by metagenomics. Pig feces was extracted using the QIAamp DNA stool minikit and QIAamp Fast DNA stool minikit and analyzed using metagenomics. The alpha diversity (Chao 1 and Shannon index) was determined at species level. The microbial community composition was examined at genus level, and the relative abundances of the 10 most abundant taxa are shown here. Download Figure S5, TIF file, 0.3 MB. Copyright © 2016 Knudsen et al. 2016 Knudsen et al. This content is distributed under the terms of the Creative Commons Attribution 4.0 International license.

**FIG 5:**
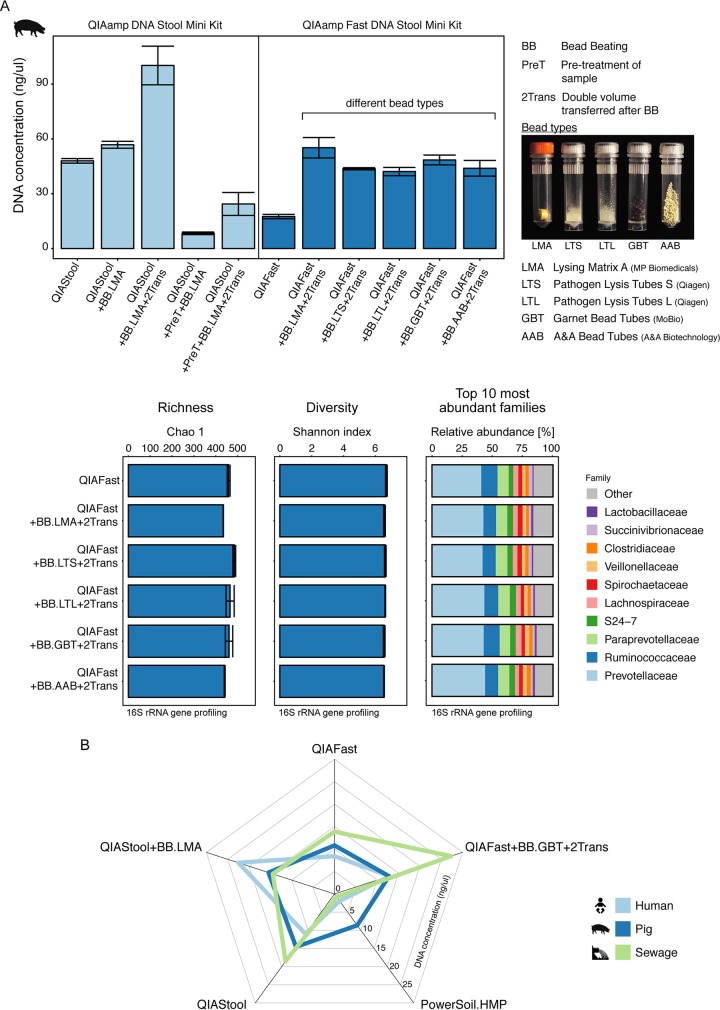
Effect of protocol modifications. (A) Pig feces was extracted using standard as well as modified protocols based on the QIAamp DNA stool minikit and QIAamp Fast DNA stool minikit. The modifications included bead beating, pretreatment of the sample, and transfer of the double amount of volume after cell lysis. In the bead beating step, different bead types were examined (for details, see Materials and Methods; Table 1). The alpha diversity (Chao 1 and Shannon index) was determined at OTU level, and the microbial community composition was examined at family level based on 16S rRNA gene profiling. (B) Selected standard and modified DNA extraction protocols were employed to extract DNA from human feces, pig feces, and sewage, and their DNA concentration was displayed in a star plot. The values indicate the averages from duplicate extractions.

Furthermore, given that our previous results suggested that including a bead beating step might result in a predicted community composition that was more similar to the community of known composition than without this step (Fig. 4), we included a bead beating step and examined the effect of beads of differing types and costs (Table 1). We obtained a higher DNA concentration using pig feces and the QIAStool kit, when bead beating was applied and the double amount of volume after cell lysis was transferred (Fig. 5A). Similarly, for the QIAFast method, we obtained on average a 2.6-fold-higher DNA concentration by including a bead beating step and transferring the double amount of volume after cell lysis, compared to DNA isolations without these modifications (Fig. 5A). Both DNA purity and stability were in the expected range (see Table S3 in the supplemental material). Even though the DNA concentration was higher with these protocol modifications, the richness, diversity, and community composition did not significantly differ when assessed by 16S rRNA gene profiling (Fig. 5A).

10.1128/mSystems.00095-16.9 Table S3  Comparison of DNA extraction methods. DNA concentration, purity, and stability, for different DNA isolation procedures based on the QIAamp DNA stool minikit and QIAamp Fast DNA stool minikit. Download Table S3, XLSX file, 0.5 MB. Copyright © 2016 Knudsen et al. 2016 Knudsen et al. This content is distributed under the terms of the Creative Commons Attribution 4.0 International license.

A particular DNA isolation method did not, however, lead to the highest DNA concentrations for each of the three types of specimen. Whereas the highest DNA concentration for sewage was achieved using the QIAFast+BB.GBT+2Trans method (27.30 ± 4.5 [SEM] ng/µl), the highest DNA concentration for human feces was obtained using the QIAStool+BB.LMA method (22.50 ± 4.77 [SEM] ng/µl) (Fig. 5B). For pig feces, the highest DNA concentrations were obtained using the QIAStool+BB.LMA (15.43 ± 3.48 [SEM] ng/µl) and QIAStool (14.57 ± 3.62 [SEM] ng/µl) methods. On average across the three types of specimens, the highest DNA concentrations were obtained using the QIAFast+BB.GBT+2Trans (17.66 ± 4.82 [SEM] ng/µl) and QIAStool+BB.LMA (17.46 ± 2.54 [SEM] ng/µl) methods.

## DISCUSSION

Genomics-based investigations of complex microbiomes greatly enhance our understanding about microbial community composition and function relevant to human, animal, and plant health; infectious diseases; environmental pollution; agriculture; and food safety. One current ambitious goal is to establish a global surveillance system for infectious agents and antimicrobial resistance based on next-generation DNA sequencing approaches (15). Given that infectious agents occupy various ecological habitats, DNA needs to be extracted from various types of specimen using standardized approaches in a time- and cost-efficient manner. It is advantageous if a range of different specimens can be processed using the same standard operating procedure (SOP). In light of these considerations, we compared eight commercially available DNA isolation kits (a total of 16 protocols) and based on the findings developed an improved protocol using the QIAamp Fast DNA stool minikit.

Overall, the amounts of DNA obtained from each DNA isolation method differed greatly, and there was no significant correlation between increasing DNA amount and increase in community diversity or richness. The taxonomic microbiome composition appeared to be dependent on both the specimen and the DNA isolation method. For example, the Easy-DNA procedure preferentially extracted DNA from Gram-positive bacteria from the human feces and hospital sewage, while the FastDNA procedure preferentially extracted DNA from Gram-positive bacteria from pig feces. Methods that did not include a bead beating or enzymatic treatment step generally extracted less DNA from Gram-positive bacteria. Furthermore, the results from our experiment that included the detection of spiked bacteria (Gram negative and Gram positive) suggest that quantification of distinct organisms from complex specimens is more challenging when the organisms are present at lower abundance levels. Inherent specimen properties may influence the DNA isolation efficiency, leading to a biased pattern of microbial community composition.

When using a particular procedure, we found some similar abundance patterns of specific bacterial families among the three specimen types. However, we also observed several differences (e.g., Fig. 2 and 3). Hence, one cannot conclude that the DNA from a particular bacterial family will be extracted preferentially using one specific DNA isolation method across different types of specimens. This could be due to different inherent cellular properties of the taxa belonging to a specific family, affecting mechanical and enzymatic cell lysis. Moreover, the chemical and physical composition of the specimen could influence DNA isolation and downstream procedures. For example, it is well known that certain compounds, such as humic acid, polysaccharides, and bilirubin, can affect PCR (16). Furthermore, fecal sample consistency, reflecting differences in water content and activity, can impact microbial community composition (17).

Our observations from 16S rRNA gene profiling and metagenomics generally agreed, but the taxonomic patterns also exhibited some differences. One reason could be the known primer biases toward certain taxa in 16S rRNA gene-based analysis (18). An additional reason could be differences in the composition of the reference databases used for the two sequence-based strategies. While 16S rRNA gene databases are composed of 16S rRNA gene sequences from a high diversity of taxa, the metagenomic sequence databases are based on whole- and draft genome sequences from fewer and less diverse taxa. The two strategies complement each other, and efforts are ongoing in developing harmonized analytical workflows for sequence-based microbial community analysis.

Based on the insight gained in this study, we have developed an improved DNA isolation method based on the QIAamp Fast DNA stool minikit. This procedure includes a bead beating step to obtain DNA from both Gram-positive and Gram-negative taxa and a step in which the double amount of cell lysate is transferred to the column to increase the DNA quantity. For aqueous sample types, like sewage, additional modifications are included, such as increasing the input amount and processing aliquots of it in parallel, as described in the standard operating procedure (SOP). While there was no single approach among the 16 procedures tested that appeared to completely resolve all challenges, we found the SOP based on the QIAamp Fast DNA stool minikit useful for a number of reasons, including the following: (i) DNA extracts contained large amounts of DNA (sufficient to permit PCR-free metagenomic sequencing) with high reproducibility, (ii) DNA extracts were of high quality in terms of DNA purity and stability, (iii) DNA from both Gram-positive and Gram-negative bacteria was reasonably well extracted (including from *Bifidobacteria*) as determined by 16S rRNA amplicon profiling and metagenomic sequencing of spiked and unspiked complex samples, (iv) the method worked well for all examined sample types based on the DNA quality assessment and inferred microbiota composition, (v) the reagents and materials required were cheaper, and (vi) the time needed for carrying out the DNA isolation was shorter than for several of the other procedures. A standard operating procedure for this DNA isolation method is available from https://dx.doi.org/10.6084/m9.figshare.3475406; it can be used for different specimen types and may be relevant to projects like EFFORT-against-AMR, COMPARE-Europe, the International Microbiome Initiative, and International Human Microbiome Standards.

In summary, our findings provide new insight into the effect of different specimen types and DNA isolation methods on DNA quantities and genomics-based inference of microbiome composition. We offer an optimized strategy for DNA isolation for different sample types providing a representative insight into community composition and which can be conducted in a time- and cost-efficient manner.

## MATERIALS AND METHODS

### Specimen collection and handling.

Human fecal specimens were collected from a healthy individual. Pig fecal specimens were collected from animals at a conventional pig production farm in Denmark. Untreated sewage was collected from the sewage inlet of the Herlev Hospital wastewater treatment plant, Denmark. For details regarding sample handling and processing, see the supplemental materials and methods (see Text S1 in the supplemental material).

### Spiking with strain mix.

Subsequent to specimen collection, about half of the aliquots from the human, pig, and sewage samples were spiked with representatives of Gram-positive and Gram-negative bacteria, namely, *Staphylococcus aureus* ST398 (strain S0385) and *Salmonella enterica* serotype Typhimurium DT104. For details regarding the preparation of the strain mix, see the supplemental materials and methods (see Text S1 in the supplemental material).

### DNA isolation.

In a first step, seven DNA isolation procedures were examined, namely, InnuPure C16 from Analytic Jena AG (InnuPure), MagNA Pure LC DNA isolation kit III from Roche (MagNA Pure), Easy-DNA genomic DNA (gDNA) purification kit from Invitrogen (Easy-DNA), MP FastDNA Spin kit from MP Biomedicals (FastDNA), PowerSoil DNA isolation kit from MoBio (PowerSoil.HMP), QIAamp DNA stool minikit from Qiagen (QIAStool), and QIAamp DNA stool minikit plus bead beating from Qiagen (QIAStool+BB) (Table 1 and details below). In a second step, a variety of modifications to two Qiagen kits were examined, namely, the QIAamp DNA stool minikit (QIAStool) and the QIAamp Fast DNA stool minikit (QIAFast). The standard operating procedure for an improved DNA isolation method (i.e., QIAamp Fast DNA stool modified, corresponding to QIAFast+BB.GBT+2Trans described here) can be found at https://dx.doi.org/10.6084/m9.figshare.3475406. For details regarding the individual DNA isolation procedures, see the supplemental materials and methods (see Text S1 in the supplemental material).

### DNA quantitation and quality assessment.

Subsequent to DNA isolation, the DNA was portioned into 10-µl aliquots to prevent repeated freeze-thawing cycles and stored at −20°C. DNA concentrations were measured using the Qubit double-stranded DNA (dsDNA) BR assay kit on a Qubit 2.0 fluorometer (Invitrogen, Carlsbad, CA). As DNA extracts can contain contaminants such as proteins and other organic molecules that can affect downstream procedures such as DNA amplifications in PCR, we determined the DNA purity by measuring the ratios of absorbance at 260/280 and 260/230 using a NanoDrop 1000 spectrophotometer (Thermo Scientific, Pittsburgh, PA, USA). DNA extracts with a 260/280 ratio between ~1.7 and ~2.0 and a 260/230 ratio between ~2.0 and ~2.2 are regarded as “pure.” The stability of the DNA in the extracts was determined by measuring the DNA concentration after 2 and 7 days of incubation at 22°C. A decrease in DNA concentration over time can indicate the presence of DNases in the extract.

### 16S rRNA gene profiling.

16S rRNA gene amplicon libraries were generated using a two-step protocol similar to that described in the document Part 15044223 Rev. B. from Illumina. In a first PCR, the V4 region of the 16S rRNA genes was amplified using the universal primers 515f (5′-TGCCAGCAGCCGCGGTAATAC) (19) and 806r (5′-GGACTACNNGGGTATCTAAT) (20). The samples were pooled in equal concentrations and concentrated using the DNA Clean and Concentrator-5 kit (Zymo Research, Orange, CA). Paired-end 2 × 250-bp sequencing of barcoded amplicons was performed on a MiSeq machine running v2 chemistry (Illumina Inc., San Diego, CA, USA). The sequences were processed using the UPARSE pipeline (21), and an OTU × sample contingency table was created. Using QIIME1.8.0 (22), taxonomy was assigned with uclust using assign_taxonomy.py based on the Greengenes 13.8 reference database. Ecological diversity estimates and microbial community comparisons were performed using the relevant scripts provided by QIIME, phyloseq, and R (22–24). For details regarding the 16S rRNA gene-based microbial community analysis, see the supplemental materials and methods (see Text S1 in the supplemental material) and the additional material provided through Figshare, https://figshare.com/projects/DNA_Isolation_Methodology_for_Microbiome_Genomics/14774.

### Metagenomics.

A subset of the DNA extracts was subjected to metagenomic sequencing. The samples were prepared and sequenced according to the Nextera XT DNA library preparation guide for the MiSeq system Part 15031942 Rev. D, using paired-end v2 2 × 250-bp sequencing. The taxonomic microbiome compositions were determined through the use of the MGmapper pipeline (T. N. Petersen, O. Lukjancenko, M. C. F. Thomsen, M. M. Sperotto, O. Lund, F. M. Aarestrup, and T. Sicheritz-Pontén, unpublished data). For details regarding the metagenomics-based microbial community analysis, see the supplemental materials and methods (see Text S1 in the supplemental material).

### Differential abundance analysis.

In order to test for the differential abundance of taxa that may drive the differences observed between the communities derived from the different DNA isolation procedures, we performed DESeq2 analyses. The read count tables from the 16S rRNA gene profiling and metagenomics sequence analysis were aggregated to the family level in R (v.3.2.3, 64 bit) (24) We performed an analysis that allows for varied sequencing depth, as suggested previously (25), and carried out two-sided Wald tests as implemented in the DESeq2 (v.1.10.1) package (26). The size factors were determined by DESeq2 from the read count tables. For details regarding the differential abundance analysis, see the supplemental materials and methods (see Text S1 in the supplemental material).

### Quantification of strain mix.

The samples that were spiked with the strain mix composed of *S. enterica* Typhimurium DT104 and *S. aureus* ST398 were extracted, sequenced, and analyzed together with the nonspiked samples. For each type of specimen and isolation method, the abundances of *Enterobacteriaceae* and *Staphylococcaceae* for 16S rRNA gene profiling and metagenomics, respectively, were determined. The ratio between *Enterobacteriaceae* and *Staphylococcaceae* was determined for each sample matrix and isolation method and compared to the *S. enterica* Typhimurium DT104/*S. aureus* ST398 ratio of CFU that were added to the original samples. For details regarding the quantification of the strain mix, see the supplemental materials and methods (see Text S1 in the supplemental material).

### Ethics.

The collection of human and pig fecal specimens as well as sewage was noninvasive, was performed in accordance with the Declaration of Helsinki, and complied with Danish and European directives (86/609/EEC). The collection of specimens was conducted in accordance with the Act on Research Ethics of Health Research Projects as administered and confirmed by the National Committee on Health Research Ethics of Denmark (Region Hovedstaden), Journal nr. H-14013582.

### Accession numbers.

The 16S rRNA gene sequences are available through the INSDC, such as from the European Nucleotide Archive (ENA) at the European Bioinformatics Institute (EBI) under accession number PRJEB12431, and the metagenomic sequences are available from ENA at EBI under accession number PRJEB14814.
