# Supplementary figures and images for: Impact of Sample Type and DNA Isolation Procedure on Genomic Inference of Microbiome Composition
Source: mSystems. 2016 Oct 18;1(5):e00095-16. doi: 10.1128/mSystems.00095-16 (PMC5080404; doi:10.1128/mSystems.00095-16)

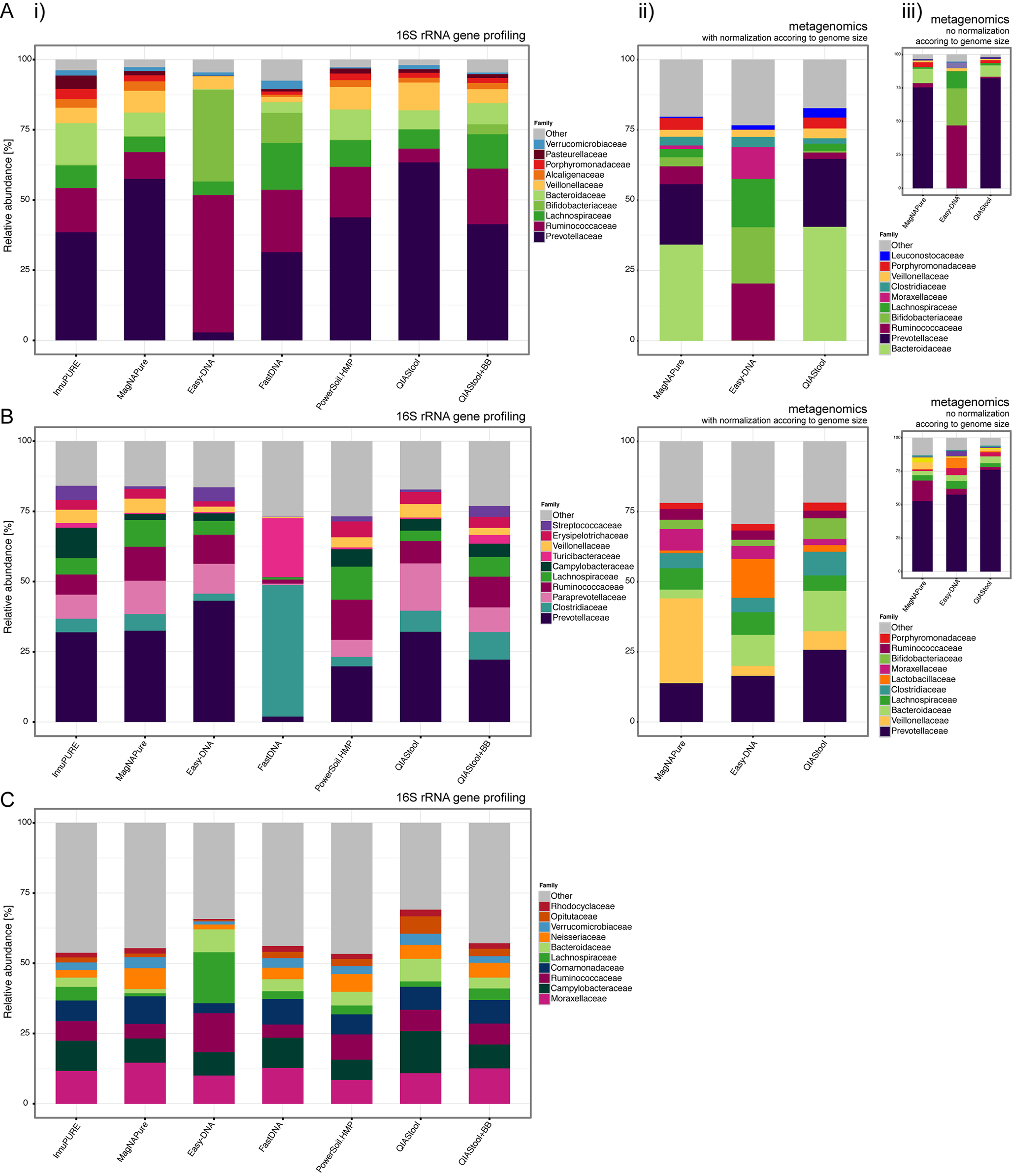

Supplement: Figure S1 [file sys005162057sf1.tif]

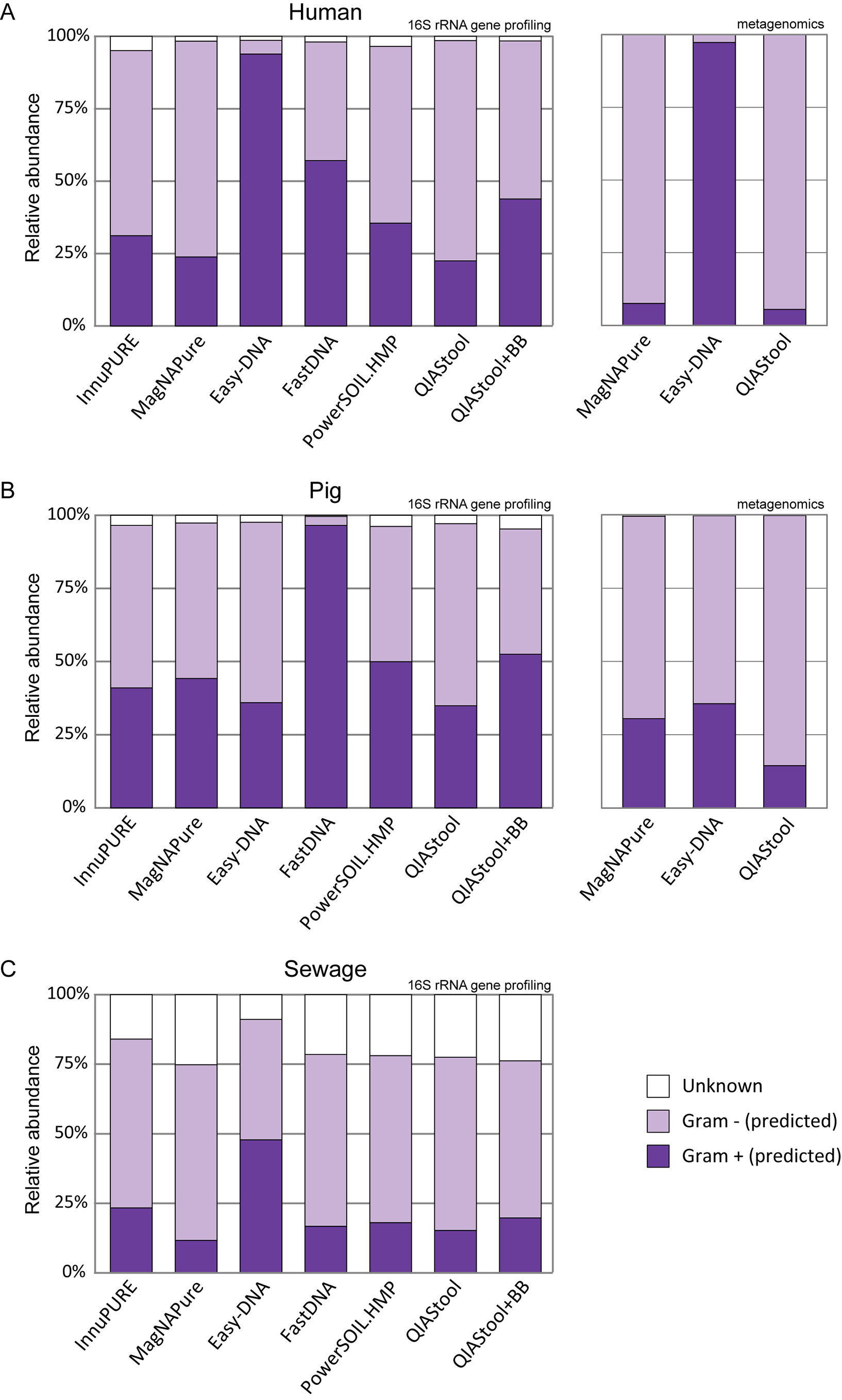

Supplement: Figure S2 [file sys005162057sf2.tif]

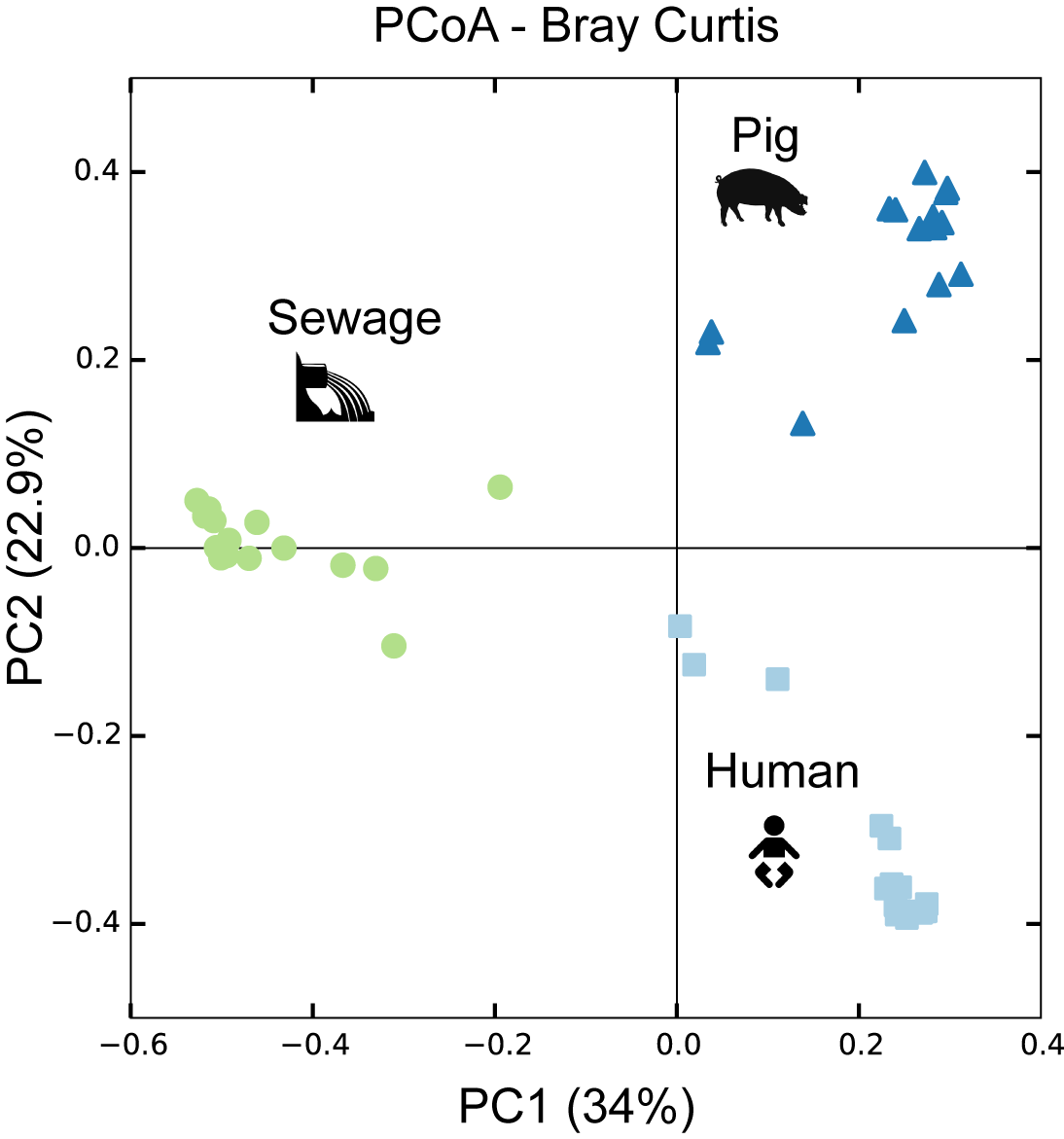

Supplement: Figure S3 [file sys005162057sf3.tif]

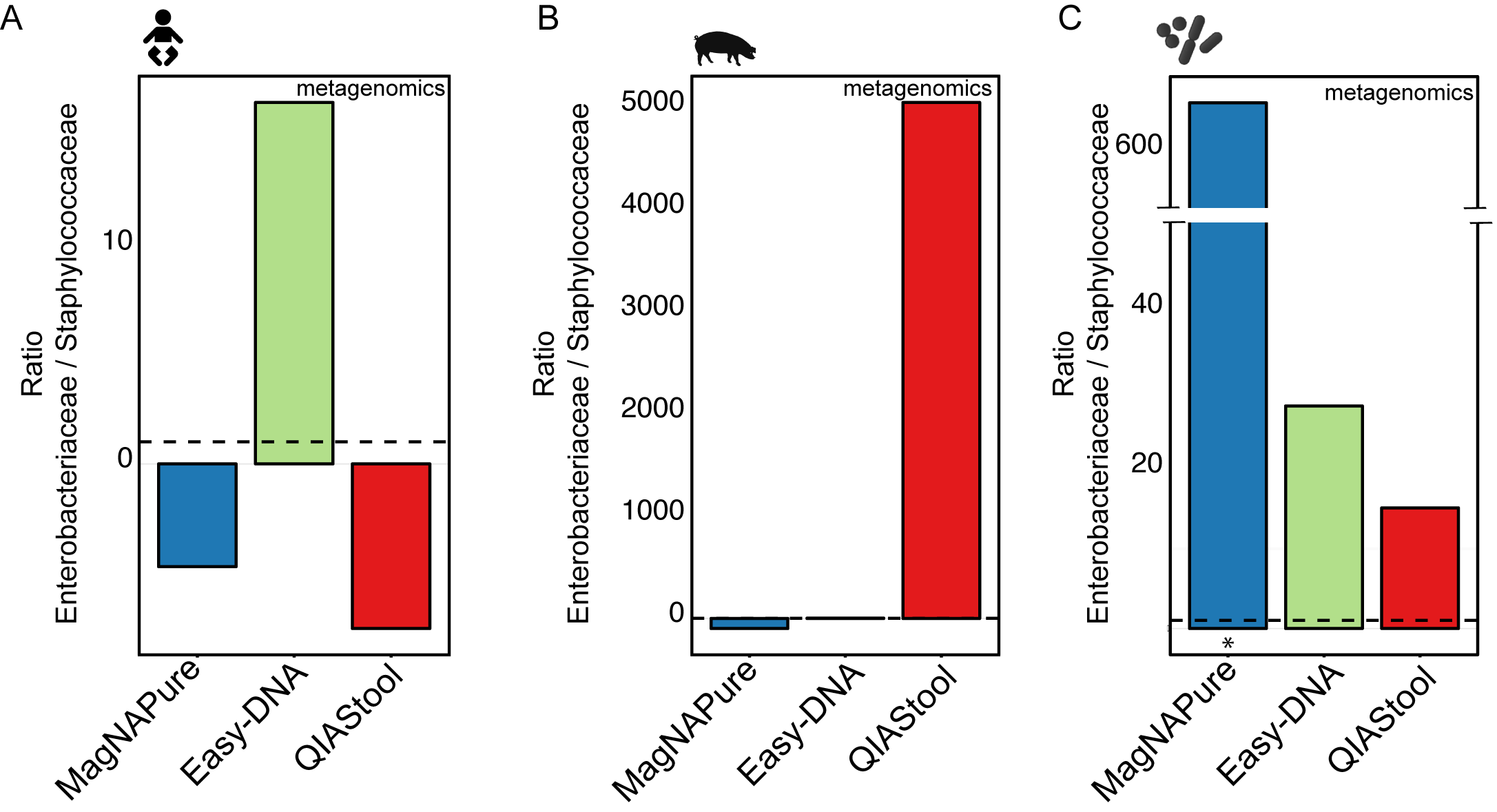

Supplement: Figure S4 [file sys005162057sf4.tif]

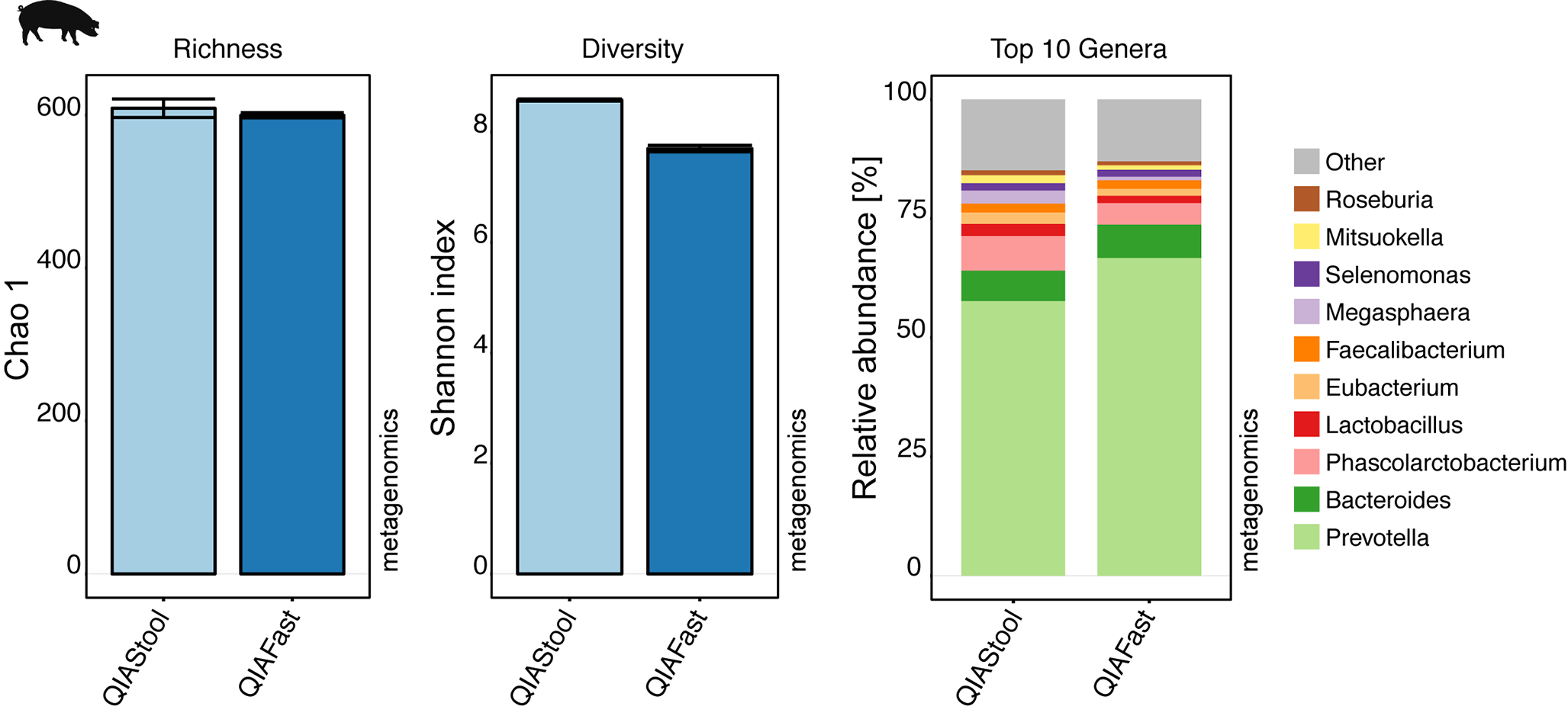

Supplement: Figure S5 [file sys005162057sf5.tif]
