# Supplementary material for: Impact of Sample Type and DNA Isolation Procedure on Genomic Inference of Microbiome Composition
Source: mSystems. 2016 Oct 18;1(5):e00095-16. doi: 10.1128/mSystems.00095-16 (PMC5080404; doi:10.1128/mSystems.00095-16)
Supplement: Table S2 [file sys005162057st2.pdf]

Table S2A. Differential abundance of families - Human fecal microbial community

baseMean	log2FoldCha	lfcSE	stat	pvalue	padj	family	compare
8087.80815	9.58965882	0.59563182	16.0999775	2.55E-58	7.49E-57	Bifidobacteriaceae	EasyDNA vs QIAStool
261.894528	9.6387835	0.59914643	16.0875255	3.12E-58	7.49E-57	[Mogibacteriaceae]	EasyDNA vs QIAStool
261.894528	6.9943178	0.50812435	13.7649727	4.14E-43	2.19E-41	[Mogibacteriaceae]	EasyDNA vs InnuPURE
261.894528	5.98959231	0.47453074	12.6221376	1.59E-36	9.88E-35	[Mogibacteriaceae]	EasyDNA vs FastDNA
261.894528	5.68437867	0.45525408	12.4861675	8.88E-36	4.26E-34	[Mogibacteriaceae]	EasyDNA vs QIAStool+BB
8087.80815	8.06288475	0.6580903	12.251943	1.64E-34	4.35E-33	Bifidobacteriaceae	EasyDNA vs InnuPURE
8087.80815	8.10323384	0.66343913	12.2139824	2.62E-34	1.52E-32	Bifidobacteriaceae	EasyDNA vs MagNAPure
261.894528	7.46735031	0.63949976	11.6768617	1.67E-31	4.85E-30	[Mogibacteriaceae]	EasyDNA vs MagNAPure
195.592384	7.11547535	0.63330982	11.2353782	2.73E-29	4.37E-28	Coriobacteriaceae	EasyDNA vs QIAStool
8087.80815	8.22252999	0.81577634	10.0793925	6.81E-24	3.13E-22	Bifidobacteriaceae	EasyDNA vs PowerSoil.HMP
118.367167	4.14939619	0.43086714	9.6303379	5.95E-22	7.14E-21	Streptococcaceae	EasyDNA vs QIAStool
261.894528	6.50010878	0.67196014	9.67335475	3.91E-22	9.00E-21	[Mogibacteriaceae]	EasyDNA vs PowerSoil.HMP
195.592384	6.52674567	0.70556721	9.25035285	2.24E-20	3.95E-19	Coriobacteriaceae	EasyDNA vs InnuPURE
8087.80815	6.05120873	0.6598584	9.17046551	4.71E-20	1.70E-18	Bifidobacteriaceae	FastDNA vs QIAStool
750.272969	-5.5100567	0.62548793	-8.8092135	1.26E-18	1.67E-17	Porphyromonadaceae	EasyDNA vs InnuPURE
750.272969	-4.8876587	0.57173354	-8.5488402	1.24E-17	1.19E-16	Porphyromonadaceae	EasyDNA vs QIAStool
750.272969	-5.3905037	0.62605815	-8.6102285	7.29E-18	1.41E-16	Porphyromonadaceae	EasyDNA vs MagNAPure
195.592384	6.37027321	0.76210202	8.35881943	6.33E-17	9.19E-16	Coriobacteriaceae	EasyDNA vs MagNAPure
195.592384	5.59941114	0.66873123	8.37318631	5.61E-17	1.35E-15	Coriobacteriaceae	EasyDNA vs QIAStool+BB
14192.9873	4.1205321	0.50198036	8.20855245	2.24E-16	1.79E-15	Ruminococcaceae	EasyDNA vs QIAStool
3174.43554	-3.2374316	0.4150328	-7.8004235	6.17E-15	9.84E-14	Veillonellaceae	FastDNA vs QIAStool
195.592384	5.37934136	0.69281817	7.76443455	8.20E-15	9.84E-14	Coriobacteriaceae	FastDNA vs QIAStool
8087.80815	5.04874196	0.65458206	7.71292439	1.23E-14	1.97E-13	Bifidobacteriaceae	EasyDNA vs QIAStool+BB
195.592384	7.26194948	0.9934254	7.31000989	2.67E-13	4.10E-12	Coriobacteriaceae	EasyDNA vs PowerSoil.HMP
188.732855	-5.3331196	0.7367495	-7.2387149	4.53E-13	5.25E-12	[Barnesiellaceae]	EasyDNA vs MagNAPure
3052.94009	-4.8906164	0.69105732	-7.0770053	1.47E-12	1.56E-11	Bacteroidaceae	EasyDNA vs InnuPURE
3052.94009	-4.7809223	0.6911788	-6.9170558	4.61E-12	4.46E-11	Bacteroidaceae	EasyDNA vs MagNAPure
680.114734	-5.3983716	0.79094213	-6.8252422	8.78E-12	7.75E-11	Pasteurellaceae	EasyDNA vs InnuPURE
3052.94009	-4.2181346	0.62453115	-6.754082	1.44E-11	9.86E-11	Bacteroidaceae	EasyDNA vs QIAStool
750.272969	-5.1128	0.75653447	-6.7581851	1.40E-11	1.61E-10	Porphyromonadaceae	EasyDNA vs PowerSoil.HMP
3174.43554	-3.1274017	0.45366454	-6.8936438	5.44E-12	2.23E-10	Veillonellaceae	FastDNA vs MagNAPure
118.367167	3.1468372	0.46933577	6.70487405	2.02E-11	2.42E-10	Streptococcaceae	EasyDNA vs QIAStool+BB
188.732855	-4.4807982	0.6822668	-6.5675161	5.12E-11	3.07E-10	[Barnesiellaceae]	EasyDNA vs QIAStool
50.0848862	-6.0497647	0.91762758	-6.5928322	4.32E-11	3.58E-10	[Odoribacteraceae]	EasyDNA vs MagNAPure
8087.80815	-4.5409169	0.65998484	-6.8803351	5.97E-12	5.73E-10	Bifidobacteriaceae	QIAStool vs QIAStool+BB
12.0964552	6.19822102	0.96500816	6.42297263	1.34E-10	7.13E-10	Actinomycetaceae	EasyDNA vs QIAStool
118.367167	3.04916807	0.47421688	6.429902	1.28E-10	9.67E-10	Streptococcaceae	EasyDNA vs InnuPURE
750.272969	-4.0416602	0.62575935	-6.4588091	1.06E-10	1.01E-09	Porphyromonadaceae	EasyDNA vs QIAStool+BB
50.0848862	-5.8312903	0.91432374	-6.3777085	1.80E-10	1.19E-09	[Odoribacteraceae]	EasyDNA vs InnuPURE
8087.80815	4.56478376	0.72120492	6.32938523	2.46E-10	5.05E-09	Bifidobacteriaceae	FastDNA vs MagNAPure
188.732855	-4.5085906	0.73616033	-6.1244683	9.10E-10	5.36E-09	[Barnesiellaceae]	EasyDNA vs InnuPURE
8087.80815	4.52443467	0.7163253	6.31617319	2.68E-10	6.19E-09	Bifidobacteriaceae	FastDNA vs InnuPURE
195.592384	4.79061167	0.75894457	6.31220231	2.75E-10	6.19E-09	Coriobacteriaceae	FastDNA vs InnuPURE
261.894528	-3.9544048	0.64127777	-6.1664462	6.98E-10	3.35E-08	[Mogibacteriaceae]	QIAStool vs QIAStool+BB
3174.43554	2.51360524	0.41567177	6.04709158	1.47E-09	4.57E-08	Veillonellaceae	EasyDNA vs FastDNA
680.114734	-4.0974239	0.72004633	-5.6905004	1.27E-08	6.08E-08	Pasteurellaceae	EasyDNA vs QIAStool
52.7372451	-4.7893782	0.84104417	-5.6945621	1.24E-08	6.56E-08	Rikenellaceae	EasyDNA vs InnuPURE
680.114734	-4.5127186	0.79165921	-5.7003298	1.20E-08	8.67E-08	Pasteurellaceae	EasyDNA vs MagNAPure
55.8474481	4.94136357	0.85181641	5.80097251	6.59E-09	9.01E-08	Lactobacillaceae	FastDNA vs MagNAPure
195.592384	4.63413921	0.81137436	5.71146864	1.12E-08	1.15E-07	Coriobacteriaceae	FastDNA vs MagNAPure
118.367167	3.5108296	0.62265509	5.6384821	1.72E-08	1.58E-07	Streptococcaceae	EasyDNA vs PowerSoil.HMP
129.697728	-2.7013853	0.46425926	-5.8187	5.93E-09	1.90E-07	Erysipelotrichaceae	QIAStool vs QIAStool+BB
261.894528	3.6491912	0.65484847	5.57257343	2.51E-08	2.26E-07	[Mogibacteriaceae]	FastDNA vs QIAStool
3052.94009	-4.6400461	0.84608129	-5.4841611	4.15E-08	3.19E-07	Bacteroidaceae	EasyDNA vs PowerSoil.HMP
14192.9873	3.01986302	0.55817717	5.4102231	6.29E-08	5.04E-07	Ruminococcaceae	EasyDNA vs QIAStool+BB
12.8572515	4.68299174	0.87546116	5.34917137	8.84E-08	6.36E-07	Turicibacteraceae	FastDNA vs QIAStool
129.697728	2.47103163	0.46631338	5.29907944	1.16E-07	6.98E-07	Erysipelotrichaceae	FastDNA vs QIAStool
14192.9873	2.93847088	0.55833808	5.26288821	1.42E-07	9.14E-07	Ruminococcaceae	EasyDNA vs MagNAPure
14192.9873	2.89591167	0.55820633	5.18788757	2.13E-07	1.02E-06	Ruminococcaceae	EasyDNA vs InnuPURE
8087.80815	3.53845009	0.6544545	5.40671674	6.42E-08	1.06E-06	Bifidobacteriaceae	EasyDNA vs FastDNA
750.272969	-3.3818974	0.62676068	-5.3958354	6.82E-08	1.06E-06	Porphyromonadaceae	EasyDNA vs FastDNA
12.0964552	5.12847194	0.99982132	5.12938846	2.91E-07	1.28E-06	Actinomycetaceae	EasyDNA vs InnuPURE
188.732855	-4.5189874	0.86857022	-5.2027888	1.96E-07	1.29E-06	[Barnesiellaceae]	EasyDNA vs PowerSoil.HMP

128.300954	-3.9366676	0.7636346	-5.1551718	2.53E-07	1.47E-06	[Paraprevotellaceae]	EasyDNA vs MagNAPure
52.7372451	-4.3339548	0.84802512	-5.1106443	3.21E-07	1.69E-06	Rikenellaceae	EasyDNA vs MagNAPure
8087.80815	4.6840799	0.86240099	5.43144078	5.59E-08	1.87E-06	Bifidobacteriaceae	FastDNA vs PowerSoil.HMP
195.592384	5.52581549	1.03014075	5.36413637	8.13E-08	1.87E-06	Coriobacteriaceae	FastDNA vs PowerSoil.HMP
18839.0105	-4.1923769	0.82982709	-5.052109	4.37E-07	1.91E-06	Prevotellaceae	EasyDNA vs QIAStool
833.096381	-4.9687451	0.98221289	-5.0587252	4.22E-07	2.04E-06	Alcaligenaceae	EasyDNA vs MagNAPure
3052.94009	-3.5171962	0.69110702	-5.0892208	3.60E-07	2.21E-06	Bacteroidaceae	EasyDNA vs QIAStool+BB
188.732855	-3.7434477	0.73628464	-5.0842398	3.69E-07	2.21E-06	[Barnesiellaceae]	EasyDNA vs QIAStool+BB
12.0964552	4.43642641	0.88398443	5.0186703	5.20E-07	2.77E-06	Actinomycetaceae	EasyDNA vs QIAStool+BB
717.960343	2.58858006	0.5178777	4.99843893	5.78E-07	2.77E-06	Verrucomicrobiaceae	EasyDNA vs QIAStool+BB
55.8474481	3.13191814	0.62594371	5.00351402	5.63E-07	2.90E-06	Lactobacillaceae	FastDNA vs QIAStool
14192.9873	2.87901713	0.55820744	5.15761149	2.50E-07	3.10E-06	Ruminococcaceae	EasyDNA vs FastDNA
118.367167	2.38319975	0.48150613	4.94946913	7.44E-07	3.32E-06	Streptococcaceae	EasyDNA vs MagNAPure
129.697728	2.12324888	0.43267331	4.90727959	9.23E-07	3.69E-06	Erysipelotrichaceae	EasyDNA vs QIAStool
55.8474481	4.67527328	0.85115964	5.49282774	3.96E-08	3.80E-06	Lactobacillaceae	QIAStool+BB vs MagNAPure
118.367167	2.38330929	0.46888891	5.08288693	3.72E-07	3.84E-06	Streptococcaceae	EasyDNA vs FastDNA
128.300954	-3.3578675	0.69956976	-4.7999038	1.59E-06	5.86E-06	[Paraprevotellaceae]	EasyDNA vs QIAStool
52.7372451	-3.7686587	0.79504946	-4.7401563	2.14E-06	7.32E-06	Rikenellaceae	EasyDNA vs QIAStool
18839.0105	-4.359738	0.91400032	-4.7699524	1.84E-06	7.63E-06	Prevotellaceae	EasyDNA vs MagNAPure
3174.43554	-2.7465167	0.55002666	-4.9934247	5.93E-07	9.10E-06	Veillonellaceae	FastDNA vs PowerSoil.HMP
128.300954	-3.603787	0.76190786	-4.7299512	2.25E-06	9.16E-06	[Paraprevotellaceae]	EasyDNA vs InnuPURE
195.592384	3.86327715	0.72509531	5.32795768	9.93E-08	9.54E-06	Coriobacteriaceae	FastDNA vs QIAStool+BB
50.0848862	-4.1453968	0.88843557	-4.665951	3.07E-06	9.83E-06	[Odoribacteraceae]	EasyDNA vs QIAStool
50.0848862	-4.7429127	1.00547833	-4.717071	2.39E-06	1.38E-05	[Odoribacteraceae]	EasyDNA vs PowerSoil.HMP
50.0848862	-4.157899	0.9175784	-4.5313829	5.86E-06	2.56E-05	[Odoribacteraceae]	EasyDNA vs QIAStool+BB
129.697728	2.38013376	0.4664827	5.10229799	3.36E-07	3.22E-05	Erysipelotrichaceae	InnuPURE vs QIAStool
680.114734	-4.3059154	0.9546601	-4.5104173	6.47E-06	3.31E-05	Pasteurellaceae	EasyDNA vs PowerSoil.HMP
833.096381	-3.9165388	0.89595272	-4.3713677	1.23E-05	3.70E-05	Alcaligenaceae	EasyDNA vs QIAStool
833.096381	-4.2614855	0.98211348	-4.3390969	1.43E-05	5.42E-05	Alcaligenaceae	EasyDNA vs InnuPURE
188.732855	-3.231528	0.73880545	-4.3739905	1.22E-05	0.00010307	[Barnesiellaceae]	EasyDNA vs FastDNA
12.0964552	3.88737674	0.89260058	4.35511339	1.33E-05	0.00010307	Actinomycetaceae	EasyDNA vs FastDNA
55.8474481	-2.8658279	0.62501984	-4.585179	4.54E-06	0.00010886	Lactobacillaceae	QIAStool vs QIAStool+BB
680.114734	-3.3086423	0.79130163	-4.1812656	2.90E-05	0.00011596	Pasteurellaceae	EasyDNA vs QIAStool+BB
24.9775852	-3.3034167	0.79477138	-4.1564363	3.23E-05	0.00012499	S24-7	EasyDNA vs MagNAPure
164.683395	2.16861441	0.52508211	4.13004815	3.63E-05	0.00016321	Clostridiaceae	FastDNA vs QIAStool
3174.43554	1.83931522	0.41386092	4.44428346	8.82E-06	0.00016932	Veillonellaceae	QIAStool vs QIAStool+BB
52.7372451	-3.9821991	0.96797847	-4.1139335	3.89E-05	0.00017893	Rikenellaceae	EasyDNA vs PowerSoil.HMP
3174.43554	-1.9851163	0.45364194	-4.3759541	1.21E-05	0.00018135	Veillonellaceae	FastDNA vs InnuPURE
50.0848862	-3.8686456	0.92385007	-4.1875253	2.82E-05	0.00019428	[Odoribacteraceae]	EasyDNA vs FastDNA
12.8572515	4.81248863	1.14323741	4.20952688	2.56E-05	0.00020984	Turicibacteraceae	FastDNA vs MagNAPure
52.7372451	-3.2306201	0.84373606	-3.8289464	0.00012869	0.00047517	Rikenellaceae	EasyDNA vs QIAStool+BB
12.8572515	3.82479629	0.94129838	4.06331974	4.84E-05	0.00054427	Turicibacteraceae	FastDNA vs InnuPURE
2.90835369	5.801207	1.54291246	3.75990677	0.00016998	0.00058278	Flavobacteriaceae	EasyDNA vs QIAStool+BB
24.9775852	-2.6872704	0.73960427	-3.6333895	0.00027972	0.0007898	S24-7	EasyDNA vs QIAStool
20.5947041	-2.5767744	0.70109054	-3.6753803	0.0002375	0.00086092	Desulfovibrionaceae	EasyDNA vs MagNAPure
118.367167	1.7660869	0.47972483	3.68145821	0.0002319	0.00092762	Streptococcaceae	FastDNA vs QIAStool
20.5947041	-2.2850151	0.64393127	-3.5485389	0.00038737	0.001033	Desulfovibrionaceae	EasyDNA vs QIAStool
8087.80815	3.05449188	0.72131966	4.2345884	2.29E-05	0.00109906	Bifidobacteriaceae	QIAStool+BB vs MagNAPure
717.960343	2.05393444	0.51665248	3.97546612	7.02E-05	0.00112386	Verrucomicrobiaceae	QIAStool vs QIAStool+BB
717.960343	2.34984392	0.64833575	3.62442444	0.00028961	0.00121108	Verrucomicrobiaceae	EasyDNA vs PowerSoil.HMP
55.8474481	3.38899548	0.87536361	3.87152888	0.00010815	0.00124378	Lactobacillaceae	FastDNA vs PowerSoil.HMP
14192.9873	2.47838617	0.69245926	3.57910753	0.00034477	0.00132162	Ruminococcaceae	EasyDNA vs PowerSoil.HMP
833.096381	-4.1016476	1.16606691	-3.5175062	0.00043562	0.00154143	Alcaligenaceae	EasyDNA vs PowerSoil.HMP
3052.94009	-2.5171693	0.6913973	-3.6406987	0.0002719	0.00156067	Bacteroidaceae	EasyDNA vs FastDNA
55.8474481	-2.3828644	0.6553515	-3.6360097	0.00027689	0.00156067	Lactobacillaceae	EasyDNA vs FastDNA
2.90835369	5.46873449	1.5682075	3.48725183	0.00048801	0.00169597	Flavobacteriaceae	EasyDNA vs InnuPURE
24.9775852	-2.7362663	0.78754989	-3.4744037	0.00051199	0.00169597	S24-7	EasyDNA vs InnuPURE
833.096381	-3.3916737	0.98214712	-3.4533255	0.00055372	0.00177191	Alcaligenaceae	EasyDNA vs QIAStool+BB
50.0848862	-2.1811191	0.60399625	-3.6111467	0.00030485	0.00196263	[Odoribacteraceae]	FastDNA vs MagNAPure
164.683395	2.091138	0.58305007	3.5865496	0.00033508	0.00196263	Clostridiaceae	FastDNA vs MagNAPure
164.683395	-1.8905223	0.53660316	-3.5231293	0.00042648	0.0022035	Clostridiaceae	EasyDNA vs FastDNA
1.64901163	5.58253937	1.67724193	3.32840438	0.00087345	0.00220661	Corynebacteriaceae	EasyDNA vs QIAStool
680.114734	-2.7600494	0.79209328	-3.4845004	0.00049306	0.0023515	Pasteurellaceae	EasyDNA vs FastDNA
18839.0105	-3.0749852	0.91400099	-3.3643128	0.00076735	0.00239231	Prevotellaceae	EasyDNA vs InnuPURE
8087.80815	-3.0141428	0.71644091	-4.2071059	2.59E-05	0.00248315	Bifidobacteriaceae	InnuPURE vs QIAStool+BB

11.6781466	2.99916116	0.92454824	3.2439207	0.00117897	0.00282952	Staphylococcaceae	EasyDNA vs QIAStool
52.7372451	-2.5787944	0.72537335	-3.555127	0.0003778	0.00340017	Rikenellaceae	FastDNA vs InnuPURE
717.960343	2.18530557	0.56391756	3.87522171	0.00010653	0.00350549	Verrucomicrobiaceae	FastDNA vs QIAStool+BB
12.8572515	3.50252691	0.90541725	3.86841197	0.00010955	0.00350549	Turicibacteraceae	FastDNA vs QIAStool+BB
55.8474481	-2.1167741	0.65447232	-3.2343219	0.00121932	0.00365796	Lactobacillaceae	EasyDNA vs QIAStool+BB
128.300954	-2.9732328	0.91355445	-3.2545765	0.00113562	0.00373131	[Paraprevotellaceae]	EasyDNA vs PowerSoil.HMP
12.0964552	3.6784619	1.13856961	3.23077471	0.00123455	0.00378596	Actinomycetaceae	EasyDNA vs PowerSoil.HMP
33.4361572	2.81733334	0.86547361	3.25525042	0.00113292	0.00386527	Enterobacteriaceae	EasyDNA vs MagNAPure
1.82114912	5.23886708	1.65581045	3.16392923	0.00155655	0.00402037	Sphingobacteriaceae	EasyDNA vs QIAStool+BB
128.300954	-2.4114715	0.76277926	-3.1614277	0.00156998	0.00402037	[Paraprevotellaceae]	EasyDNA vs QIAStool+BB
33.4361572	2.54187917	0.80503432	3.15747925	0.0015914	0.00402037	Enterobacteriaceae	EasyDNA vs QIAStool+BB
3174.43554	-1.7292853	0.45259586	-3.8208156	0.00013301	0.00425635	Veillonellaceae	QIAStool+BB vs MagNAPure
12.0964552	2.92089424	0.9106931	3.20733102	0.00133973	0.0043169	Actinomycetaceae	EasyDNA vs MagNAPure
261.894528	2.6444657	0.67913295	3.89388516	9.87E-05	0.00473527	[Mogibacteriaceae]	InnuPURE vs QIAStool
18839.0105	-3.4408077	1.0966717	-3.1375002	0.00170395	0.00479902	Prevotellaceae	EasyDNA vs PowerSoil.HMP
2.73031934	-5.5948167	1.78991433	-3.1257455	0.00177355	0.00479902	Aerococcaceae	EasyDNA vs PowerSoil.HMP
55.8474481	3.46324375	0.85610471	4.04535067	5.22E-05	0.00501551	Lactobacillaceae	InnuPURE vs MagNAPure
717.960343	-2.0716363	0.56501412	-3.6665213	0.00024587	0.00590094	Verrucomicrobiaceae	QIAStool+BB vs MagNAPure
18839.0105	-2.7588902	0.91399679	-3.01849	0.00254038	0.00609691	Prevotellaceae	EasyDNA vs QIAStool+BB
3.42322903	4.0050664	1.33425048	3.00173503	0.00268446	0.0061359	Pseudomonadaceae	EasyDNA vs QIAStool+BB
750.272969	-2.1281593	0.63659993	-3.343009	0.00082875	0.00621564	Porphyromonadaceae	FastDNA vs InnuPURE
50.0848862	-1.9626447	0.59872204	-3.2780565	0.00104524	0.00671943	[Odoribacteraceae]	FastDNA vs InnuPURE
55.8474481	2.55849915	0.84174411	3.03952131	0.00236954	0.00723335	Lactobacillaceae	EasyDNA vs MagNAPure
3052.94009	-2.3734471	0.74564072	-3.1830975	0.00145709	0.00779712	Bacteroidaceae	FastDNA vs InnuPURE
680.114734	-2.6383223	0.83401698	-3.1633915	0.00155942	0.00779712	Pasteurellaceae	FastDNA vs InnuPURE
261.894528	3.13867473	0.8067979	3.89028617	0.00010013	0.00795635	[Mogibacteriaceae]	PowerSoil.HMP vs QIAStool
129.697728	2.20203213	0.58468421	3.76619048	0.00016576	0.00795635	Erysipelotrichaceae	PowerSoil.HMP vs QIAStool
2.73031934	-5.9403078	1.78353589	-3.3306354	0.00086648	0.00797162	Aerococcaceae	FastDNA vs PowerSoil.HMP
750.272969	-2.0086063	0.63715947	-3.1524389	0.00161913	0.00829803	Porphyromonadaceae	FastDNA vs MagNAPure
12.8572515	2.52115646	0.87157181	2.89265487	0.00382001	0.00873145	Turicibacteraceae	EasyDNA vs QIAStool
12.8249831	2.47583548	0.80177935	3.08792622	0.00201559	0.00907013	Peptostreptococcaceae	FastDNA vs InnuPURE
12.8572515	3.81865729	1.18165368	3.23162137	0.0012309	0.0094369	Turicibacteraceae	FastDNA vs PowerSoil.HMP
24.9775852	-2.6351797	0.91756811	-2.8719173	0.0040799	0.0104264	S24-7	EasyDNA vs PowerSoil.HMP
3052.94009	-2.2637531	0.74575245	-3.0355288	0.00240114	0.01086238	Bacteroidaceae	FastDNA vs MagNAPure
188.732855	-2.1015916	0.6991929	-3.0057393	0.00264936	0.01086238	[Barnesiellaceae]	FastDNA vs MagNAPure
128.300954	-2.2809055	0.7707057	-2.9595026	0.00308136	0.01148507	[Paraprevotellaceae]	FastDNA vs MagNAPure
717.960343	2.05912098	0.56396613	3.65114298	0.00026108	0.01253164	Verrucomicrobiaceae	InnuPURE vs QIAStool+BB
52.7372451	-2.1233709	0.7336022	-2.8944446	0.0037983	0.01297752	Rikenellaceae	FastDNA vs MagNAPure
2.90835369	4.49112126	1.61342038	2.78360267	0.00537588	0.0130153	Flavobacteriaceae	EasyDNA vs PowerSoil.HMP
3174.43554	1.11548884	0.41450174	2.69115597	0.00712049	0.01529563	Veillonellaceae	EasyDNA vs QIAStool+BB
11.6781466	2.68984272	1.00310889	2.6815062	0.00732916	0.01529563	Staphylococcaceae	EasyDNA vs QIAStool+BB
129.697728	-1.7885614	0.47769996	-3.7441104	0.00018103	0.01536327	Erysipelotrichaceae	QIAStool vs MagNAPure
118.367167	-1.7661964	0.4920531	-3.5894428	0.00033139	0.01536327	Streptococcaceae	QIAStool vs MagNAPure
50.0848862	-1.9043678	0.54541108	-3.4916192	0.0004801	0.01536327	[Odoribacteraceae]	QIAStool vs MagNAPure
12.8249831	-2.3955975	0.82460766	-2.9051361	0.00367093	0.01625699	Peptostreptococcaceae	EasyDNA vs FastDNA
833.096381	-2.9212505	1.04951845	-2.7834198	0.00537892	0.01696427	Alcaligenaceae	FastDNA vs MagNAPure
8087.80815	-3.173788	0.86249486	-3.6797762	0.00023344	0.01712544	Bifidobacteriaceae	PowerSoil.HMP vs QIAStool+BB
55.8474481	-3.1229052	0.87472716	-3.5701477	0.00035678	0.01712544	Lactobacillaceae	PowerSoil.HMP vs QIAStool+BB
11.6781466	2.79196033	1.02507165	2.72367334	0.00645603	0.01900943	Staphylococcaceae	EasyDNA vs InnuPURE
24.9775852	-2.0060866	0.78814538	-2.5453256	0.01091759	0.02183518	S24-7	EasyDNA vs QIAStool+BB
3.42322903	-4.3875174	1.50304307	-2.9190896	0.00351055	0.02298506	Pseudomonadaceae	FastDNA vs PowerSoil.HMP
164.683395	1.99810261	0.70063696	2.85183728	0.00434673	0.02298506	Clostridiaceae	FastDNA vs PowerSoil.HMP
717.960343	1.94656943	0.68516789	2.84101089	0.00449708	0.02298506	Verrucomicrobiaceae	FastDNA vs PowerSoil.HMP
1.64901163	4.49276703	1.71286008	2.62296208	0.0087169	0.025279	Corynebacteriaceae	EasyDNA vs MagNAPure
1.82114912	4.39593515	1.68839485	2.60361796	0.00922455	0.02547733	Sphingobacteriaceae	EasyDNA vs MagNAPure
24.9775852	-1.8300403	0.6984518	-2.6201383	0.00878941	0.02574042	S24-7	FastDNA vs MagNAPure
50.0848862	-1.8918656	0.59388004	-3.1856023	0.00144453	0.02773498	[Odoribacteraceae]	QIAStool+BB vs MagNAPure
1.64901163	4.22462547	1.68481228	2.50747548	0.0121597	0.02796731	Corynebacteriaceae	EasyDNA vs PowerSoil.HMP
1.82114912	4.10868763	1.67143497	2.45817976	0.01396433	0.03058852	Sphingobacteriaceae	EasyDNA vs PowerSoil.HMP
195.592384	1.736134	0.65109372	2.66648864	0.00766482	0.03168125	Coriobacteriaceae	EasyDNA vs FastDNA
3.42322903	4.86140167	1.48339223	3.27721931	0.00104835	0.03354717	Pseudomonadaceae	PowerSoil.HMP vs QIAStool+BB
3.42322903	3.53118218	1.35773544	2.6007881	0.00930099	0.03403901	Pseudomonadaceae	EasyDNA vs FastDNA
18839.0105	-2.3758961	0.91401379	-2.5994094	0.00933843	0.03403901	Prevotellaceae	EasyDNA vs FastDNA
52.7372451	-2.2105839	0.85684169	-2.579921	0.00988229	0.03403901	Rikenellaceae	EasyDNA vs FastDNA
750.272969	-1.5057613	0.58394147	-2.5786168	0.00991968	0.03571084	Porphyromonadaceae	FastDNA vs QIAStool

33.4361572	2.28011708	0.9191566	2.48066226	0.01311386	0.03584454	Enterobacteriaceae	FastDNA vs MagNAPure
12.8572515	-2.1618353	0.86641893	-2.4951386	0.0125908	0.04108575	Turicibacteraceae	EasyDNA vs FastDNA
20.5947041	-1.6911916	0.69469488	-2.434438	0.01491493	0.04160481	Desulfovibrionaceae	EasyDNA vs InnuPURE
3052.94009	-1.7009654	0.68492375	-2.4834376	0.01301211	0.0425851	Bacteroidaceae	FastDNA vs QIAStool
3.96627812	2.57800025	1.13190334	2.27757986	0.02275162	0.04368311	Comamonadaceae	EasyDNA vs QIAStool+BB
128.300954	-1.9480249	0.76899668	-2.5332033	0.01130254	0.04623766	[Paraprevotellaceae]	FastDNA vs InnuPURE
33.4361572	1.66163853	0.72289861	2.29857756	0.02152894	0.04635089	Enterobacteriaceae	EasyDNA vs QIAStool
1.29933147	3.68974709	1.61352576	2.28676057	0.0222098	0.04635089	Xanthomonadaceae	EasyDNA vs QIAStool
33.4361572	1.91151815	0.80544298	2.37325075	0.01763229	0.04672557	Enterobacteriaceae	EasyDNA vs InnuPURE
128.300954	-1.7021055	0.70735254	-2.4063043	0.01611483	0.0483445	[Paraprevotellaceae]	FastDNA vs QIAStool
3174.43554	-1.3981164	0.45362294	-3.0821114	0.00205538	0.0493291	Veillonellaceae	FastDNA vs QIAStool+BB
12.8572515	2.65065335	1.14044478	2.32422771	0.02011329	0.05302595	Turicibacteraceae	EasyDNA vs MagNAPure
52.7372451	-1.5580748	0.67048579	-2.3238	0.02013622	0.05576183	Rikenellaceae	FastDNA vs QIAStool
50.0848862	1.68589343	0.53952737	3.1247598	0.0017795	0.0569441	[Odoribacteraceae]	InnuPURE vs QIAStool
20.5947041	-1.4101301	0.61823455	-2.2808982	0.02255447	0.05779583	Desulfovibrionaceae	FastDNA vs MagNAPure
3174.43554	-1.2523153	0.41388181	-3.02578	0.00247993	0.05951824	Veillonellaceae	InnuPURE vs QIAStool
0.882185	4.0371131	1.78622755	2.26013371	0.02381295	0.06009936	Gemellaceae	EasyDNA vs InnuPURE
2.90835369	2.46128406	1.15648797	2.12824009	0.03331718	0.06663437	Flavobacteriaceae	EasyDNA vs QIAStool
14192.9873	1.24151496	0.55827928	2.2238242	0.02616027	0.06726928	Ruminococcaceae	FastDNA vs QIAStool
12.0964552	-3.2773268	1.09799283	-2.9848344	0.00283732	0.06809565	Actinomycetaceae	QIAStool vs MagNAPure
1.64901163	3.77525052	1.6511205	2.2864779	0.02222632	0.06890158	Corynebacteriaceae	EasyDNA vs FastDNA
1.64901163	3.58183657	1.63651122	2.18870272	0.02861845	0.06894446	Corynebacteriaceae	EasyDNA vs InnuPURE
164.683395	1.34909893	0.57279168	2.3553047	0.01850752	0.06940321	Clostridiaceae	FastDNA vs InnuPURE
0.882185	4.01816686	1.78597331	2.24984709	0.02445865	0.07095808	Gemellaceae	EasyDNA vs FastDNA
1.82114912	3.54033456	1.58145933	2.2386504	0.02517867	0.07095808	Sphingobacteriaceae	EasyDNA vs FastDNA
2.73031934	4.97244546	1.63772663	3.03618771	0.0023959	0.07666885	Aerococcaceae	PowerSoil.HMP vs QIAStool
1.29933147	3.36579674	1.65782097	2.03025344	0.04233078	0.07814914	Xanthomonadaceae	EasyDNA vs QIAStool+BB
3052.94009	-2.1228769	0.89019477	-2.384733	0.01709152	0.07862099	Bacteroidaceae	FastDNA vs PowerSoil.HMP
261.894528	1.477758	0.69172546	2.136336	0.03265204	0.07874903	[Mogibacteriaceae]	FastDNA vs MagNAPure
12.0964552	2.31084429	1.08368228	2.13240017	0.03297396	0.07913751	Actinomycetaceae	FastDNA vs QIAStool
680.114734	-1.7526692	0.83469225	-2.099779	0.03574829	0.08142665	Pasteurellaceae	FastDNA vs MagNAPure
128.300954	-1.6557621	0.76773858	-2.1566743	0.03103105	0.08364892	[Paraprevotellaceae]	EasyDNA vs FastDNA
11.6015749	-2.2504463	1.0243547	-2.1969405	0.0280247	0.09112658	Campylobacteraceae	FastDNA vs InnuPURE
55.8474481	1.47811982	0.67420109	2.19240201	0.02835049	0.09112658	Lactobacillaceae	FastDNA vs InnuPURE
0.882185	3.39994624	1.70295575	1.99649711	0.04587984	0.09593057	Gemellaceae	EasyDNA vs PowerSoil.HMP
20.5947041	-1.1183708	0.55185161	-2.0265789	0.04270549	0.09608736	Desulfovibrionaceae	FastDNA vs QIAStool
833.096381	-2.0474946	0.98295866	-2.0829916	0.03725199	0.09623431	Alcaligenaceae	EasyDNA vs FastDNA
1.19877439	3.49866232	1.81740826	1.92508331	0.05421894	0.09638922	Cryomorphaceae	EasyDNA vs QIAStool+BB
1.82114912	3.1528737	1.55024005	2.0337971	0.04197206	0.09671822	Sphingobacteriaceae	EasyDNA vs InnuPURE
18839.0105	-1.9838419	0.98946509	-2.004964	0.04496689	0.09703382	Prevotellaceae	FastDNA vs MagNAPure
2.73031934	-3.6259805	1.69469176	-2.1396106	0.03238625	0.09715874	Aerococcaceae	FastDNA vs InnuPURE
2.90835369	2.67380324	1.29756082	2.06063809	0.03933758	0.0975572	Flavobacteriaceae	EasyDNA vs FastDNA
11.6781466	2.16807721	1.05257629	2.05978154	0.03941943	0.0977461	Staphylococcaceae	EasyDNA vs MagNAPure
0.882185	3.61138826	1.76237664	2.04915804	0.04044666	0.0977461	Gemellaceae	EasyDNA vs MagNAPure
833.096381	-2.2139909	1.04942688	-2.1097143	0.03488297	0.09810835	Alcaligenaceae	FastDNA vs InnuPURE
3174.43554	-0.7238264	0.37173907	-1.9471356	0.05151847	0.09891547	Veillonellaceae	EasyDNA vs QIAStool
18839.0105	-1.8164808	0.91354221	-1.9883929	0.04676826	0.09903866	Prevotellaceae	FastDNA vs QIAStool
750.272969	-1.7309025	0.76559114	-2.2608707	0.02376727	0.09939039	Porphyromonadaceae	FastDNA vs PowerSoil.HMP
188.732855	-1.2492702	0.64121456	-1.9482873	0.0513806	0.09998639	[Barnesiellaceae]	FastDNA vs QIAStool
833.096381	-1.8690442	0.97057026	-1.9257176	0.05413965	0.09998639	Alcaligenaceae	FastDNA vs QIAStool
24.9775852	-1.213894	0.63403122	-1.9145651	0.05554799	0.09998639	S24-7	FastDNA vs QIAStool

Table S2B. Differential abundance of families - Pig fecal microbial community

baseMean	log2FoldCha	lfcSE	stat	pvalue	padj	family	compare
6226.33527	8.23337231	0.2901671	28.3745898	4.16E-177	2.96E-175	Turicibacteraceae	FastDNA vs QIAStool
6226.33527	-8.9600238	0.31794514	-28.181037	9.99E-175	7.99E-173	Turicibacteraceae	EasyDNA vs FastDNA
1329.00016	-7.3100807	0.26522532	-27.561776	3.20E-167	1.28E-165	Peptostreptococcaceae	EasyDNA vs FastDNA
1329.00016	6.29759341	0.23184427	27.1630318	1.78E-162	6.31E-161	Peptostreptococcaceae	FastDNA vs QIAStool
6226.33527	9.16361787	0.33891741	27.0379083	5.30E-161	3.44E-159	Turicibacteraceae	FastDNA vs MagNAPure
1329.00016	6.95674527	0.26345927	26.4053921	1.19E-153	6.18E-152	Peptostreptococcaceae	FastDNA vs InnuPURE
1329.00016	6.24786129	0.26803077	23.310239	3.49E-120	1.13E-118	Peptostreptococcaceae	FastDNA vs MagNAPure
6226.33527	8.37938709	0.35930749	23.3209362	2.72E-120	1.88E-118	Turicibacteraceae	FastDNA vs PowerSoil.HMP
14966.648	-7.3777612	0.32913136	-22.415856	2.76E-111	7.35E-110	Clostridiaceae	EasyDNA vs FastDNA
6226.33527	6.98112067	0.31642099	22.0627609	7.20E-108	1.87E-106	Turicibacteraceae	FastDNA vs InnuPURE
1329.00016	6.57818334	0.30059891	21.8835905	3.72E-106	1.28E-104	Peptostreptococcaceae	FastDNA vs PowerSoil.HMP
1329.00016	6.11331273	0.30715449	19.903055	3.83E-88	2.18E-86	Peptostreptococcaceae	FastDNA vs QIAStool+BB
14966.648	7.01386817	0.36120224	19.4181191	5.42E-84	1.25E-82	Clostridiaceae	FastDNA vs PowerSoil.HMP
14966.648	6.69216818	0.34940405	19.153093	9.12E-82	1.58E-80	Clostridiaceae	FastDNA vs InnuPURE
14966.648	6.16102466	0.34985453	17.6102467	2.06E-69	4.45E-68	Clostridiaceae	FastDNA vs MagNAPure
14966.648	5.44539513	0.318726	17.0848791	1.92E-65	4.55E-64	Clostridiaceae	FastDNA vs QIAStool
6226.33527	6.27791748	0.38345515	16.3719732	3.03E-60	8.64E-59	Turicibacteraceae	FastDNA vs QIAStool+BB
14966.648	5.80051888	0.42495209	13.6498186	2.02E-42	3.85E-41	Clostridiaceae	FastDNA vs QIAStool+BB
254.614729	-2.6451948	0.27545666	-9.6029437	7.77E-22	5.05E-20	S24-7	InnuPURE vs QIAStool
35.1041071	2.34749529	0.28446002	8.25246117	1.55E-16	1.18E-14	Peptococcaceae	EasyDNA vs QIAStool
576.775751	2.79069511	0.38546037	7.23990142	4.49E-13	8.98E-12	Streptococcaceae	EasyDNA vs FastDNA
408.703374	3.64804379	0.50165879	7.27196224	3.54E-13	3.51E-11	Lactobacillaceae	EasyDNA vs MagNAPure
254.614729	-1.8657731	0.26216238	-7.1168605	1.10E-12	4.20E-11	S24-7	EasyDNA vs QIAStool
6226.33527	2.88570039	0.40420787	7.13914937	9.39E-13	1.09E-10	Turicibacteraceae	QIAStool+BB vs MagNAPure
576.775751	2.21032995	0.32171271	6.87050862	6.40E-12	1.62E-10	Streptococcaceae	EasyDNA vs QIAStool
2576.15115	-2.3625033	0.3468527	-6.8112582	9.67E-12	1.72E-10	[Paraprevotellaceae]	FastDNA vs QIAStool
6226.33527	-2.6821064	0.38685912	-6.9330312	4.12E-12	2.06E-10	Turicibacteraceae	EasyDNA vs QIAStool+BB
576.775751	2.51571595	0.36417115	6.90805937	4.91E-12	2.43E-10	Streptococcaceae	EasyDNA vs MagNAPure
35.1041071	2.71384929	0.40707004	6.66678715	2.61E-11	8.63E-10	Peptococcaceae	EasyDNA vs MagNAPure
14966.648	-1.9323661	0.29731783	-6.4993278	8.07E-11	1.53E-09	Clostridiaceae	EasyDNA vs QIAStool
576.775751	-2.5501711	0.41120073	-6.201767	5.58E-10	7.26E-09	Streptococcaceae	FastDNA vs InnuPURE
6226.33527	2.1824972	0.34136207	6.39349657	1.62E-10	8.92E-09	Turicibacteraceae	InnuPURE vs MagNAPure
22.9687795	-4.1589171	0.64930852	-6.4051478	1.50E-10	9.46E-09	Helicobacteraceae	EasyDNA vs InnuPURE
254.614729	-1.8689828	0.30680193	-6.0918223	1.12E-09	1.59E-08	S24-7	FastDNA vs QIAStool
254.614729	-1.8826504	0.30472804	-6.1781333	6.49E-10	1.78E-08	S24-7	InnuPURE vs MagNAPure
1019.42191	-2.9556395	0.49110705	-6.0183202	1.76E-09	1.83E-08	Campylobacteraceae	FastDNA vs InnuPURE
6226.33527	-1.9789032	0.32055255	-6.1734127	6.68E-10	2.11E-08	Turicibacteraceae	EasyDNA vs InnuPURE
408.703374	3.99766567	0.64482803	6.19958424	5.66E-10	3.28E-08	Lactobacillaceae	QIAStool+BB vs MagNAPure
22.9687795	3.05927367	0.51002005	5.99834003	1.99E-09	6.48E-08	Helicobacteraceae	InnuPURE vs QIAStool
576.775751	2.27519194	0.39134713	5.81374372	6.11E-09	1.12E-07	Streptococcaceae	InnuPURE vs MagNAPure
62.3051996	-3.6585641	0.613405	-5.9643533	2.46E-09	1.87E-07	Enterobacteriaceae	EasyDNA vs PowerSoil.HMP
408.703374	2.43422116	0.43645264	5.57728586	2.44E-08	3.71E-07	Lactobacillaceae	EasyDNA vs QIAStool
576.775751	1.96980595	0.35224663	5.59212148	2.24E-08	4.86E-07	Streptococcaceae	InnuPURE vs QIAStool
2576.15115	-1.9272471	0.34922351	-5.5186637	3.42E-08	2.22E-06	[Paraprevotellaceae]	PowerSoil.HMP vs QIAStool
216.46708	6.27411432	1.18075226	5.31365855	1.07E-07	3.22E-06	Bacteroidaceae	PowerSoil.HMP vs QIAStool
62.3051996	2.92721007	0.55710414	5.25433196	1.49E-07	3.22E-06	Enterobacteriaceae	PowerSoil.HMP vs QIAStool
367.01539	-2.774783	0.54483681	-5.09287	3.53E-07	3.63E-06	Spirochaetaceae	FastDNA vs QIAStool
7308.6798	-1.2094339	0.23761089	-5.0899766	3.58E-07	3.63E-06	Prevotellaceae	FastDNA vs QIAStool
71.0660634	-2.1428817	0.41434301	-5.1717579	2.32E-07	3.77E-06	Alcaligenaceae	InnuPURE vs QIAStool
7308.6798	1.22465113	0.23872154	5.13004044	2.90E-07	4.63E-06	Prevotellaceae	EasyDNA vs FastDNA
62.3051996	-3.2981065	0.61505214	-5.3623202	8.22E-08	4.85E-06	Enterobacteriaceae	InnuPURE vs PowerSoil.HMP
2516.24433	1.09546943	0.21932759	4.99467231	5.89E-07	5.11E-06	Ruminococcaceae	FastDNA vs InnuPURE
6226.33527	-1.9554548	0.36446037	-5.3653428	8.08E-08	5.98E-06	Turicibacteraceae	QIAStool vs QIAStool+BB
35.1041071	1.49262726	0.29814049	5.00645612	5.54E-07	7.21E-06	Peptococcaceae	InnuPURE vs QIAStool
408.703374	2.70224502	0.53923011	5.01130217	5.41E-07	7.43E-06	Lactobacillaceae	InnuPURE vs MagNAPure
1019.42191	-2.2116186	0.45182578	-4.8948483	9.84E-07	8.73E-06	Campylobacteraceae	FastDNA vs QIAStool
88.4317884	3.06001619	0.6165416	4.96319502	6.93E-07	9.25E-06	Coriobacteriaceae	EasyDNA vs FastDNA
254.614729	-1.514942	0.30301081	-4.9996301	5.74E-07	9.33E-06	S24-7	PowerSoil.HMP vs QIAStool
62.3051996	3.74759308	0.72291645	5.18399201	2.17E-07	9.99E-06	Enterobacteriaceae	PowerSoil.HMP vs QIAStool+BB
7308.6798	-1.1800909	0.23943127	-4.928725	8.28E-07	1.08E-05	Prevotellaceae	PowerSoil.HMP vs QIAStool
35.1041071	1.93628224	0.38058991	5.0875816	3.63E-07	1.23E-05	Peptococcaceae	EasyDNA vs PowerSoil.HMP
22.9687795	-3.5480864	0.70506262	-5.0322997	4.85E-07	1.23E-05	Helicobacteraceae	EasyDNA vs PowerSoil.HMP
2516.24433	-1.1212344	0.22183279	-5.0544122	4.32E-07	1.27E-05	Ruminococcaceae	InnuPURE vs PowerSoil.HMP
7308.6798	1.19530817	0.24053344	4.96940538	6.72E-07	1.28E-05	Prevotellaceae	EasyDNA vs PowerSoil.HMP
6226.33527	-2.1014696	0.42138725	-4.9870271	6.13E-07	1.41E-05	Turicibacteraceae	PowerSoil.HMP vs QIAStool+BB
14966.648	-1.568473	0.33252325	-4.7168823	2.39E-06	2.59E-05	Clostridiaceae	PowerSoil.HMP vs QIAStool
254.614729	1.65814449	0.33449335	4.95718218	7.15E-07	2.65E-05	S24-7	QIAStool vs QIAStool+BB
2576.15115	-1.5902241	0.34177933	-4.6527802	3.27E-06	3.55E-05	[Paraprevotellaceae]	InnuPURE vs QIAStool

35.1041071	3.04129082	0.65712892	4.62814943	3.69E-06	4.22E-05	Peptococcaceae	EasyDNA vs FastDNA
367.01539	-2.2519712	0.48760528	-4.6184307	3.87E-06	4.47E-05	Spirochaetaceae	EasyDNA vs QIAStool
42.1528852	3.57440506	0.77612071	4.60547568	4.12E-06	4.47E-05	Bifidobacteriaceae	EasyDNA vs QIAStool
30.2465249	5.96837557	1.30932681	4.55835437	5.16E-06	4.79E-05	Verrucomicrobiaceae	PowerSoil.HMP vs QIAStool
71.0660634	-2.5632064	0.56711788	-4.5197066	6.19E-06	4.89E-05	Alcaligenaceae	FastDNA vs QIAStool
1019.42191	-2.3003618	0.49644019	-4.6337139	3.59E-06	6.20E-05	Campylobacteraceae	FastDNA vs PowerSoil.HMP
216.46708	6.67995824	1.44979616	4.60751549	4.08E-06	6.25E-05	Bacteroidaceae	PowerSoil.HMP vs QIAStool+BB
2516.24433	-0.98295	0.21745846	-4.5201736	6.18E-06	6.80E-05	Ruminococcaceae	InnuPURE vs MagNAPure
408.703374	-2.783843	0.59619079	-4.6693829	3.02E-06	7.45E-05	Lactobacillaceae	QIAStool vs QIAStool+BB
35.1041071	1.85898126	0.41668134	4.46139786	8.14E-06	7.46E-05	Peptococcaceae	InnuPURE vs MagNAPure
908.726526	-1.6630925	0.37476149	-4.4377359	9.09E-06	8.64E-05	Veillonellaceae	EasyDNA vs QIAStool
408.703374	2.48203872	0.54739067	4.53430954	5.78E-06	9.39E-05	Lactobacillaceae	FastDNA vs MagNAPure
42.1528852	4.04263564	0.88466376	4.56968603	4.88E-06	0.00010258	Bifidobacteriaceae	EasyDNA vs InnuPURE
810.892629	-2.4775883	0.57438549	-4.3134592	1.61E-05	0.00011411	Erysipelotrichaceae	FastDNA vs QIAStool
576.775751	1.69015115	0.37888619	4.4608413	8.16E-06	0.00012409	Streptococcaceae	EasyDNA vs PowerSoil.HMP
367.01539	-2.2701322	0.52308917	-4.339857	1.43E-05	0.00013239	Spirochaetaceae	InnuPURE vs QIAStool
29.6083625	1.69887968	0.38157423	4.45229143	8.50E-06	0.00013381	[Mogibacteriaceae]	EasyDNA vs InnuPURE
88.4317884	-2.803111	0.63669121	-4.4026225	1.07E-05	0.00013904	Coriobacteriaceae	FastDNA vs MagNAPure
6226.33527	1.25225163	0.2930255	4.27352444	1.92E-05	0.00015633	Turicibacteraceae	InnuPURE vs QIAStool
23.2927606	8.01906524	1.88146584	4.26213705	2.02E-05	0.00016452	Aerococcaceae	PowerSoil.HMP vs QIAStool
22.9687795	2.448443	0.58021396	4.21989674	2.44E-05	0.00017652	Helicobacteraceae	PowerSoil.HMP vs QIAStool
32.5255849	5.81457361	1.39301324	4.17409788	2.99E-05	0.00019446	Staphylococcaceae	PowerSoil.HMP vs QIAStool
367.01539	-2.2707797	0.54976209	-4.1304771	3.62E-05	0.00021392	Spirochaetaceae	PowerSoil.HMP vs QIAStool
71.0660634	-1.6777577	0.39962713	-4.1983078	2.69E-05	0.00022709	Alcaligenaceae	EasyDNA vs QIAStool
75.3501469	-3.3526702	0.78701599	-4.2599773	2.04E-05	0.0002576	Mycoplasmataceae	EasyDNA vs InnuPURE
22.9687795	-2.9648076	0.67504024	-4.3920458	1.12E-05	0.00027792	Helicobacteraceae	EasyDNA vs MagNAPure
17.2832685	-2.2084726	0.53750076	-4.1087804	3.98E-05	0.00028727	Desulfovibrionaceae	InnuPURE vs QIAStool
30.2465249	6.56938288	1.56924588	4.18633114	2.83E-05	0.00032602	Verrucomicrobiaceae	PowerSoil.HMP vs QIAStool+BB
15.8153467	4.63369833	1.14552937	4.04502795	5.23E-05	0.00033768	Moraxellaceae	FastDNA vs QIAStool
7308.6798	1.26879368	0.29728884	4.26788197	1.97E-05	0.00036507	Prevotellaceae	QIAStool vs QIAStool+BB
32.5255849	5.54202541	1.38556172	3.99984016	6.34E-05	0.00037503	Staphylococcaceae	FastDNA vs QIAStool
1329.00016	-1.0124872	0.24978175	-4.0534877	5.05E-05	0.00038349	Peptostreptococcaceae	EasyDNA vs QIAStool
1019.42191	1.93983364	0.47935342	4.04677127	5.19E-05	0.00040801	Campylobacteraceae	InnuPURE vs MagNAPure
7308.6798	1.28401094	0.29817511	4.3062311	1.66E-05	0.00041515	Prevotellaceae	EasyDNA vs QIAStool+BB
576.775751	-2.0306009	0.48938134	-4.1493222	3.33E-05	0.00047518	Streptococcaceae	FastDNA vs QIAStool+BB
15.4343002	1.53247414	0.38535741	3.97676056	6.99E-05	0.00048267	Corynebacteriaceae	EasyDNA vs QIAStool
31.9177507	-3.2697252	0.83566211	-3.912736	9.13E-05	0.0004984	Succinivibrionaceae	FastDNA vs QIAStool
5.3314072	4.35405821	1.11876983	3.89182661	9.95E-05	0.00053892	[Methanomassiliicoccaceae]	PowerSoil.HMP vs QIAStool
14966.648	-1.2467731	0.31964806	-3.9004556	9.60E-05	0.0005811	Clostridiaceae	InnuPURE vs QIAStool
2516.24433	-0.771523	0.19809811	-3.8946512	9.83E-05	0.0005811	Ruminococcaceae	InnuPURE vs QIAStool
216.46708	5.5853327	1.27809173	4.37005621	1.24E-05	0.00064592	Bacteroidaceae	PowerSoil.HMP vs MagNAPure
62.3051996	2.61404432	0.62207451	4.20214024	2.64E-05	0.00068745	Enterobacteriaceae	PowerSoil.HMP vs MagNAPure
2576.15115	-1.514051	0.37895822	-3.995298	6.46E-05	0.00069997	[Paraprevotellaceae]	FastDNA vs MagNAPure
2516.24433	0.80433271	0.20244058	3.97317915	7.09E-05	0.00074466	Ruminococcaceae	EasyDNA vs InnuPURE
22.9687795	-2.8972138	0.74038877	-3.913098	9.11E-05	0.0009112	Helicobacteraceae	EasyDNA vs FastDNA
42.1528852	3.64689481	0.9520937	3.83039483	0.00012794	0.0009504	Bifidobacteriaceae	FastDNA vs InnuPURE
42.1528852	3.17866423	0.85340488	3.72468483	0.00019556	0.00099177	Bifidobacteriaceae	FastDNA vs QIAStool
88.4317884	1.56076323	0.41573031	3.75426855	0.00017385	0.00110104	Coriobacteriaceae	EasyDNA vs QIAStool
88.4317884	1.78168807	0.46431042	3.83727784	0.00012441	0.00111965	Coriobacteriaceae	EasyDNA vs InnuPURE
35.1041071	-1.3708732	0.34666006	-3.9545174	7.67E-05	0.001135	Peptococcaceae	QIAStool vs QIAStool+BB
1019.42191	-1.6885426	0.44454014	-3.798403	0.00014563	0.00114685	Campylobacteraceae	EasyDNA vs InnuPURE
2576.15115	1.67665813	0.42907776	3.90758571	9.32E-05	0.00114975	[Paraprevotellaceae]	QIAStool vs QIAStool+BB
22.9687795	-2.9199313	0.73813187	-3.9558396	7.63E-05	0.00127111	Helicobacteraceae	EasyDNA vs QIAStool+BB
7.44045113	2.81616091	0.73648989	3.82376043	0.00013143	0.00138942	Anaeroplasmataceae	QIAStool vs QIAStool+BB
2576.15115	1.31919125	0.34980343	3.77123584	0.00016244	0.00144392	[Paraprevotellaceae]	EasyDNA vs FastDNA
14966.648	-1.5772423	0.40923926	-3.8540836	0.00011616	0.00145205	Clostridiaceae	EasyDNA vs QIAStool+BB
6226.33527	1.39826642	0.36161179	3.86676115	0.00011029	0.0016201	Turicibacteraceae	InnuPURE vs PowerSoil.HMP
20.723165	-5.185472	1.34638073	-3.8514158	0.00011744	0.0016201	Pseudomonadaceae	InnuPURE vs PowerSoil.HMP
71.0660634	-1.8593941	0.49253655	-3.7751394	0.00015992	0.0016201	Alcaligenaceae	InnuPURE vs PowerSoil.HMP
216.46708	-4.7736527	1.26699265	-3.7677036	0.00016476	0.0016201	Bacteroidaceae	InnuPURE vs PowerSoil.HMP
216.46708	-4.9350175	1.29105875	-3.8224577	0.00013213	0.00182337	Bacteroidaceae	FastDNA vs PowerSoil.HMP
810.892629	-2.373461	0.62905633	-3.7730501	0.00016126	0.00185453	Erysipelotrichaceae	FastDNA vs PowerSoil.HMP
1329.00016	-1.1967679	0.32086779	-3.7297852	0.00019164	0.00191643	Peptostreptococcaceae	EasyDNA vs QIAStool+BB
9.6383629	-2.1679125	0.6068059	-3.5726621	0.00035337	0.00206586	Fibrobacteraceae	EasyDNA vs QIAStool
15.4343002	1.84120962	0.50412692	3.65227395	0.00025993	0.00216607	Corynebacteriaceae	EasyDNA vs QIAStool+BB
71.0660634	-1.6471299	0.4610371	-3.5726625	0.00035337	0.00242942	Alcaligenaceae	InnuPURE vs MagNAPure
216.46708	-4.4629635	1.20206834	-3.7127369	0.00020503	0.00259705	Bacteroidaceae	EasyDNA vs PowerSoil.HMP
254.614729	-1.1032288	0.29278195	-3.7680902	0.0001645	0.002651	S24-7	EasyDNA vs MagNAPure
1329.00016	-1.0622194	0.28366823	-3.7445834	0.00018069	0.002651	Peptostreptococcaceae	EasyDNA vs MagNAPure
15.4343002	1.90293704	0.50943828	3.73536328	0.00018744	0.002651	Corynebacteriaceae	EasyDNA vs MagNAPure

71.0660634	-2.2797188	0.62584767	-3.6426097	0.00026989	0.00266032	Alcaligenaceae	FastDNA vs PowerSoil.HMP
14966.648	-1.2167365	0.33050128	-3.6814881	0.00023188	0.00286947	Clostridiaceae	EasyDNA vs MagNAPure
576.775751	1.44962715	0.4050545	3.57884468	0.00034512	0.00290884	Streptococcaceae	InnuPURE vs PowerSoil.HMP
86.5992792	2.99949869	0.85659517	3.5016526	0.00046238	0.00300548	Porphyromonadaceae	FastDNA vs InnuPURE
4.16403335	5.96645628	1.75160616	3.4062773	0.00065855	0.00311715	Carnobacteriaceae	FastDNA vs QIAStool
15.8153467	5.10720894	1.40712282	3.62954026	0.00028393	0.00323676	Moraxellaceae	FastDNA vs QIAStool+BB
15.4343002	1.94734349	0.53872155	3.61474956	0.00030064	0.00326407	Corynebacteriaceae	EasyDNA vs PowerSoil.HMP
15.4343002	1.5357668	0.43958903	3.4936422	0.00047648	0.00333535	Corynebacteriaceae	EasyDNA vs InnuPURE
15.8153467	4.25898236	1.24098327	3.43194181	0.00059928	0.00346248	Moraxellaceae	FastDNA vs InnuPURE
62.3051996	-2.2078122	0.62740538	-3.5189565	0.00043325	0.00346598	Enterobacteriaceae	EasyDNA vs FastDNA
75.3501469	-2.899597	0.86109053	-3.3673544	0.00075893	0.00394644	Mycoplasmataceae	FastDNA vs InnuPURE
2576.15115	-1.0433121	0.30923781	-3.3738179	0.00074133	0.00402438	[Paraprevotellaceae]	EasyDNA vs QIAStool
23.2927606	6.98533528	1.92158786	3.63518912	0.00027778	0.00426411	Aerococcaceae	PowerSoil.HMP vs MagNAPure
30.2465249	5.15082886	1.43392754	3.59211237	0.00032801	0.00426411	Verrucomicrobiaceae	PowerSoil.HMP vs MagNAPure
30.2465249	-4.7520422	1.38719877	-3.425639	0.00061336	0.00452349	Verrucomicrobiaceae	InnuPURE vs PowerSoil.HMP
408.703374	1.97172535	0.56084378	3.51564092	0.00043869	0.00456242	Lactobacillaceae	PowerSoil.HMP vs MagNAPure
35.1041071	-2.1864228	0.662999	-3.2977769	0.00097454	0.00460689	Peptococcaceae	FastDNA vs InnuPURE
29.6083625	-1.3718487	0.40734356	-3.3677929	0.00075772	0.00463054	[Mogibacteriaceae]	InnuPURE vs MagNAPure
254.614729	-1.1302528	0.33388242	-3.3851821	0.00071131	0.00466304	S24-7	InnuPURE vs PowerSoil.HMP
35.1041071	1.73722724	0.45243877	3.83969578	0.00012319	0.00476323	Peptococcaceae	QIAStool+BB vs MagNAPure
4.00044772	5.78336799	1.78215296	3.24515803	0.00117385	0.0050867	Cytophagaceae	FastDNA vs InnuPURE
1574.16355	1.17263609	0.35784849	3.27690659	0.00104951	0.00524755	Lachnospiraceae	PowerSoil.HMP vs QIAStool
71.0660634	-2.0674546	0.60167145	-3.4361853	0.00058997	0.00547827	Alcaligenaceae	FastDNA vs MagNAPure
29.6083625	1.43379183	0.42701614	3.35769937	0.00078594	0.00561386	[Mogibacteriaceae]	EasyDNA vs QIAStool+BB
23.2927606	-6.4898889	1.90765791	-3.4020192	0.0006689	0.00576926	Aerococcaceae	FastDNA vs PowerSoil.HMP
29.6083625	1.05593006	0.32473651	3.25165181	0.00114736	0.00581332	[Mogibacteriaceae]	EasyDNA vs QIAStool
908.726526	-1.4420041	0.41614103	-3.4651814	0.00052987	0.00582861	Veillonellaceae	EasyDNA vs MagNAPure
5.3314072	-4.298708	1.25525218	-3.4245772	0.00061576	0.00584969	[Methanomassiliicoccaceae]	EasyDNA vs PowerSoil.HMP
576.775751	1.75562174	0.47291523	3.71233916	0.00020535	0.00595522	Streptococcaceae	QIAStool+BB vs MagNAPure
32.5255849	4.24277218	1.31620267	3.22349458	0.00126637	0.00601524	Staphylococcaceae	EasyDNA vs QIAStool
88.4317884	1.85557658	0.56238288	3.29948979	0.00096861	0.0060538	Coriobacteriaceae	EasyDNA vs QIAStool+BB
254.614729	-1.1064385	0.33329617	-3.3196856	0.00090119	0.00703253	S24-7	FastDNA vs MagNAPure
7308.6798	-0.8575932	0.26003373	-3.2980077	0.00097373	0.00703253	Prevotellaceae	FastDNA vs MagNAPure
5.3314072	3.37108488	1.05056705	3.2088241	0.00133279	0.00721928	[Methanomassiliicoccaceae]	InnuPURE vs QIAStool
42.1528852	-3.1585672	0.98137257	-3.21852	0.00128854	0.00760239	Bifidobacteriaceae	InnuPURE vs PowerSoil.HMP
408.703374	1.67631844	0.50776167	3.3013883	0.00096208	0.0081242	Lactobacillaceae	EasyDNA vs PowerSoil.HMP
576.775751	-1.4502357	0.44128745	-3.2863743	0.00101486	0.00856339	Streptococcaceae	QIAStool vs QIAStool+BB
908.726526	1.66195963	0.50683851	3.27907132	0.00104149	0.00856339	Veillonellaceae	QIAStool vs QIAStool+BB
20.723165	4.83629545	1.47007325	3.28983298	0.00100247	0.00922271	Pseudomonadaceae	PowerSoil.HMP vs QIAStool+BB
408.703374	1.48842239	0.47936509	3.10498703	0.00190287	0.00951437	Lactobacillaceae	InnuPURE vs QIAStool
367.01539	2.08867133	0.64924642	3.2170702	0.00129507	0.00958351	Spirochaetaceae	QIAStool vs QIAStool+BB
4.00044772	4.90531979	1.59999884	3.06582709	0.00217069	0.00963243	Cytophagaceae	FastDNA vs QIAStool
23.2927606	-6.0628195	1.88263038	-3.2203982	0.00128013	0.00972896	Aerococcaceae	EasyDNA vs PowerSoil.HMP
15.8153467	4.05506684	1.27803736	3.172886	0.00150932	0.00981057	Moraxellaceae	FastDNA vs MagNAPure
88.4317884	-1.5247829	0.49109684	-3.1048518	0.00190374	0.01047059	Coriobacteriaceae	InnuPURE vs MagNAPure
5.3314072	-3.7627421	1.19345081	-3.1528255	0.00161698	0.0108779	[Methanomassiliicoccaceae]	QIAStool vs QIAStool+BB
42.1528852	2.69033662	0.88649264	3.03480989	0.00240687	0.01117477	Bifidobacteriaceae	PowerSoil.HMP vs QIAStool
23.2927606	6.07256394	1.92001451	3.16276981	0.00156276	0.01192028	Aerococcaceae	PowerSoil.HMP vs QIAStool+BB
408.703374	-2.0259403	0.64952423	-3.1191143	0.00181396	0.01192028	Lactobacillaceae	PowerSoil.HMP vs QIAStool+BB
810.892629	-1.9297672	0.62539388	-3.0856829	0.00203085	0.01200049	Erysipelotrichaceae	FastDNA vs MagNAPure
62.3051996	1.8473546	0.62901353	2.93690754	0.00331503	0.01326012	Enterobacteriaceae	FastDNA vs InnuPURE
29.6083625	1.83023832	0.58732018	3.11625309	0.00183165	0.01332109	[Mogibacteriaceae]	EasyDNA vs FastDNA
7308.6798	-0.8282503	0.26169677	-3.1649236	0.00155124	0.01344405	Prevotellaceae	PowerSoil.HMP vs MagNAPure
810.892629	-1.5061467	0.51416507	-2.9293057	0.0033972	0.01490319	Erysipelotrichaceae	EasyDNA vs QIAStool
75.3501469	-2.1087165	0.72280839	-2.9173935	0.0035297	0.01490319	Mycoplasmataceae	EasyDNA vs QIAStool
20.723165	4.16353174	1.34986951	3.08439573	0.00203966	0.01515177	Pseudomonadaceae	PowerSoil.HMP vs MagNAPure
86.5992792	-2.5986406	0.87070194	-2.9845352	0.0028401	0.01523324	Porphyromonadaceae	InnuPURE vs PowerSoil.HMP
30.7423537	-1.6327572	0.56577317	-2.8858865	0.00390313	0.01561251	Elusimicrobiaceae	EasyDNA vs QIAStool
29.6083625	1.32991944	0.43726084	3.04147851	0.00235419	0.01626534	[Mogibacteriaceae]	EasyDNA vs PowerSoil.HMP
22.9687795	1.79757037	0.62321785	2.8843371	0.00392239	0.01638173	Helicobacteraceae	FastDNA vs QIAStool
62.3051996	2.29684119	0.73468911	3.12627637	0.00177035	0.01681833	Enterobacteriaceae	FastDNA vs QIAStool+BB
908.726526	-1.1737315	0.4109824	-2.8559168	0.00429128	0.0169267	Veillonellaceae	FastDNA vs QIAStool
22.9687795	-1.8651642	0.54282237	-3.4360489	0.00059026	0.01771725	Helicobacteraceae	QIAStool vs MagNAPure
5.3314072	-3.6505736	1.06984005	-3.4122611	0.00064426	0.01771725	[Methanomassiliicoccaceae]	QIAStool vs MagNAPure
17.2832685	-2.4187504	0.86002934	-2.8124045	0.00491726	0.01837503	Desulfovibrionaceae	FastDNA vs QIAStool
1.66132016	4.416643	1.57030905	2.81259475	0.00491435	0.01867455	Leuconostocaceae	EasyDNA vs QIAStool
4.00044772	5.29050645	1.80899893	2.92454925	0.00344955	0.01868509	Cytophagaceae	FastDNA vs MagNAPure
5.8077209	-2.1252395	0.74134296	-2.8667427	0.0041472	0.01925486	Dethiosulfovibrionaceae	InnuPURE vs QIAStool
31.9177507	-1.3581394	0.47904029	-2.8351256	0.00458077	0.01984999	Succinivibrionaceae	InnuPURE vs QIAStool
9.6383629	-1.5817218	0.5627087	-2.810907	0.00494021	0.02006959	Fibrobacteraceae	InnuPURE vs QIAStool

1574.16355	-1.0713078	0.36311938	-2.9502908	0.00317475	0.02010675	Lachnospiraceae	EasyDNA vs PowerSoil.HMP
22.9687795	-1.8202879	0.62049856	-2.9335893	0.00335067	0.02066248	Helicobacteraceae	QIAStool vs QIAStool+BB
35.1041071	-2.0646688	0.68555435	-3.0116778	0.00259808	0.02115581	Peptococcaceae	FastDNA vs QIAStool+BB
1574.16355	-1.1146482	0.39092455	-2.8513128	0.00435391	0.02140673	Lachnospiraceae	InnuPURE vs PowerSoil.HMP
71.0660634	-1.3942701	0.48029311	-2.9029567	0.00369658	0.02161076	Alcaligenaceae	EasyDNA vs PowerSoil.HMP
31.9177507	2.50733503	0.858128	2.92186602	0.00347941	0.02319608	Succinivibrionaceae	EasyDNA vs FastDNA
31.9177507	-2.4656281	0.87371635	-2.8220006	0.00477251	0.02386254	Succinivibrionaceae	FastDNA vs MagNAPure
20.723165	-4.4393022	1.50402412	-2.9516164	0.00316115	0.02423551	Pseudomonadaceae	FastDNA vs PowerSoil.HMP
35.1041071	0.97662205	0.3461751	2.82117935	0.00478474	0.02483044	Peptococcaceae	EasyDNA vs QIAStool+BB
5.3314072	-3.7073919	1.31972034	-2.8092254	0.00496609	0.02483044	[Methanomassiliicoccaceae]	EasyDNA vs QIAStool+BB
88.4317884	1.46816717	0.5184466	2.83185802	0.00462784	0.02512256	Coriobacteriaceae	EasyDNA vs PowerSoil.HMP
35.1041071	0.85486803	0.29757508	2.87278099	0.00406876	0.02563319	Peptococcaceae	EasyDNA vs InnuPURE
35.1041071	1.08141422	0.39086961	2.76668788	0.00566289	0.02570083	Peptococcaceae	InnuPURE vs PowerSoil.HMP
23.2927606	-5.16892	1.89209931	-2.7318439	0.0062981	0.02654198	Aerococcaceae	InnuPURE vs PowerSoil.HMP
30.7423537	-1.9203408	0.72425206	-2.6514814	0.00801395	0.02844954	Elusimicrobiaceae	FastDNA vs QIAStool
1329.00016	-0.6591519	0.24790456	-2.6588937	0.00783977	0.0294383	Peptostreptococcaceae	InnuPURE vs QIAStool
7308.6798	-0.6240217	0.23586236	-2.6457027	0.00815214	0.0294383	Prevotellaceae	InnuPURE vs QIAStool
7.94956632	2.35515765	0.88703846	2.65507951	0.00792897	0.02945047	Christensenellaceae	FastDNA vs InnuPURE
5.3314072	-3.5952234	1.21352904	-2.9626183	0.00305035	0.03019842	[Methanomassiliicoccaceae]	EasyDNA vs MagNAPure
2576.15115	-1.0787948	0.38112544	-2.8305504	0.0046468	0.03020419	[Paraprevotellaceae]	PowerSoil.HMP vs MagNAPure
14966.648	-1.2133493	0.43533275	-2.7871767	0.00531695	0.03057245	Clostridiaceae	PowerSoil.HMP vs QIAStool+BB
4.2789796	-4.8902304	1.84214054	-2.6546457	0.00793918	0.03122742	Xanthomonadaceae	InnuPURE vs PowerSoil.HMP
75.3501469	2.20086404	0.8067117	2.72819154	0.00636826	0.03184131	Mycoplasmataceae	InnuPURE vs MagNAPure
408.703374	1.26821609	0.4885714	2.59576407	0.00943808	0.03190972	Lactobacillaceae	FastDNA vs QIAStool
62.3051996	1.47645817	0.57256579	2.57866992	0.00991815	0.03200858	Enterobacteriaceae	FastDNA vs QIAStool
5.3314072	-3.3157346	1.1972195	-2.7695294	0.00561373	0.03215138	[Methanomassiliicoccaceae]	EasyDNA vs InnuPURE
1019.42191	-1.6633229	0.59035526	-2.8174948	0.00483999	0.03448493	Campylobacteraceae	FastDNA vs QIAStool+BB
8.45632111	5.03537667	1.90368579	2.64506711	0.00816747	0.03539238	Rikenellaceae	PowerSoil.HMP vs QIAStool
2.95003546	4.7042716	1.82149714	2.58264012	0.00980475	0.03548387	Actinomycetaceae	EasyDNA vs QIAStool
1019.42191	1.26709688	0.45944542	2.75788338	0.00581769	0.0358012	Campylobacteraceae	EasyDNA vs FastDNA
1.25729577	3.9531204	1.56655913	2.52344155	0.01162124	0.03587426	Methanobacteriaceae	FastDNA vs QIAStool
9.6383629	-1.9706676	0.68484384	-2.8775431	0.00400785	0.03607067	Fibrobacteraceae	EasyDNA vs MagNAPure
7308.6798	0.63923894	0.23698127	2.69742385	0.00698783	0.03668609	Prevotellaceae	EasyDNA vs InnuPURE
7.44045113	2.10035711	0.79576123	2.6394313	0.00830453	0.03774784	Anaeroplasmataceae	EasyDNA vs QIAStool+BB
6226.33527	0.93024556	0.31721125	2.93257433	0.00336164	0.0381879	Turicibacteraceae	QIAStool vs MagNAPure
88.4317884	-1.3038581	0.44554611	-2.9264268	0.0034288	0.0381879	Coriobacteriaceae	QIAStool vs MagNAPure
32.5255849	-3.9854437	1.38548882	-2.8765614	0.00402034	0.0381879	Staphylococcaceae	QIAStool vs MagNAPure
254.614729	0.76254431	0.27049763	2.81904245	0.00481671	0.0381879	S24-7	QIAStool vs MagNAPure
1574.16355	-0.9682183	0.35443007	-2.7317613	0.00629968	0.0381879	Lachnospiraceae	QIAStool vs MagNAPure
14.253878	2.0500995	0.75196855	2.72631018	0.00640468	0.0381879	Pirellulaceae	QIAStool vs MagNAPure
1019.42191	1.19581281	0.43898336	2.72405041	0.00644867	0.0381879	Campylobacteraceae	QIAStool vs MagNAPure
367.01539	1.41359659	0.52364085	2.69955368	0.00694326	0.0381879	Spirochaetaceae	QIAStool vs MagNAPure
88.4317884	-1.499253	0.60509226	-2.4777262	0.01322226	0.03911584	Coriobacteriaceae	FastDNA vs QIAStool
42.1528852	-2.6816693	0.99400689	-2.6978378	0.00697914	0.03972744	Bifidobacteriaceae	QIAStool vs QIAStool+BB
254.614729	0.77942167	0.29736518	2.62109257	0.00876485	0.04149089	S24-7	EasyDNA vs InnuPURE
20.723165	3.34261958	1.28892921	2.59333062	0.00950513	0.04149089	Pseudomonadaceae	EasyDNA vs InnuPURE
5.22647741	4.53504399	1.75773924	2.58004366	0.00987878	0.04149089	Sphingobacteriaceae	EasyDNA vs InnuPURE
17.2832685	1.59191715	0.59981224	2.65402579	0.00795377	0.04204138	Desulfovibrionaceae	QIAStool vs QIAStool+BB
5.22647741	5.05202816	1.82265879	2.77179041	0.00557489	0.04331319	Sphingobacteriaceae	EasyDNA vs MagNAPure
13.1827693	-2.4080017	0.87080204	-2.7652688	0.00568759	0.04331319	RF16	EasyDNA vs MagNAPure
4.16403335	4.73703395	1.82302293	2.59845001	0.00936457	0.04347835	Carnobacteriaceae	FastDNA vs MagNAPure
2.29903269	-4.3063622	1.6450049	-2.6178416	0.00884879	0.04483386	Deferribacteraceae	EasyDNA vs PowerSoil.HMP
4.16403335	4.24556219	1.70906431	2.48414419	0.01298632	0.04486184	Carnobacteriaceae	EasyDNA vs QIAStool
6226.33527	-0.7266515	0.29467249	-2.4659632	0.01366454	0.04515238	Turicibacteraceae	EasyDNA vs QIAStool
810.892629	-1.5452431	0.62495608	-2.4725627	0.01341482	0.0465047	Erysipelotrichaceae	FastDNA vs InnuPURE
13.1827693	-2.0388951	0.78399797	-2.6006383	0.00930505	0.04652525	RF16	QIAStool vs MagNAPure
1019.42191	1.28455598	0.48482082	2.64954788	0.00805996	0.04656863	Campylobacteraceae	PowerSoil.HMP vs MagNAPure
2.05453403	3.06422851	1.22184327	2.50787363	0.01214601	0.04934316	[Cerasicoccaceae]	PowerSoil.HMP vs QIAStool
2.05453403	-3.5994508	1.41189289	-2.5493795	0.01079148	0.05096371	[Cerasicoccaceae]	EasyDNA vs PowerSoil.HMP
75.3501469	-2.0844308	0.82480975	-2.5271656	0.01149873	0.05096371	Mycoplasmataceae	EasyDNA vs PowerSoil.HMP
2576.15115	0.88393503	0.352154	2.51008088	0.01207035	0.05096371	[Paraprevotellaceae]	EasyDNA vs PowerSoil.HMP
8.45632111	-4.7100683	1.89200483	-2.4894589	0.01279377	0.05117508	Rikenellaceae	EasyDNA vs PowerSoil.HMP
1329.00016	-0.708884	0.28201899	-2.5136037	0.01195046	0.05213896	Peptostreptococcaceae	InnuPURE vs MagNAPure
17.2832685	-1.5355685	0.61355598	-2.5027357	0.01232375	0.05213896	Desulfovibrionaceae	InnuPURE vs MagNAPure
4.16403335	4.13609738	1.72422814	2.39881097	0.0164484	0.05345731	Carnobacteriaceae	FastDNA vs InnuPURE
7.44045113	2.72371757	0.77809085	3.50051356	0.00046436	0.05386606	Anaeroplasmataceae	InnuPURE vs QIAStool+BB
5.8077209	-2.048274	0.83138022	-2.463703	0.013751	0.05402178	Dethiosulfovibrionaceae	InnuPURE vs MagNAPure
15.4343002	1.75738715	0.67749264	2.59395756	0.00948782	0.05421611	Corynebacteriaceae	EasyDNA vs FastDNA
29.6083625	-1.5032074	0.60411383	-2.488285	0.01283608	0.05562303	[Mogibacteriaceae]	FastDNA vs MagNAPure
14.253878	2.21225682	0.86748571	2.55019397	0.0107663	0.05598476	Pirellulaceae	PowerSoil.HMP vs MagNAPure

810.892629	-1.4020193	0.57490323	-2.4387049	0.01474	0.05601199	Erysipelotrichaceae	EasyDNA vs PowerSoil.HMP
2.29903269	-3.8386124	1.56814864	-2.4478626	0.01437064	0.05658441	Deferribacteraceae	EasyDNA vs InnuPURE
908.726526	-1.0058792	0.41567683	-2.4198587	0.01552654	0.05753952	Veillonellaceae	EasyDNA vs InnuPURE
1019.42191	-0.9445218	0.40055924	-2.3580077	0.01837331	0.05818216	Campylobacteraceae	EasyDNA vs QIAStool
71.0660634	-1.1820059	0.44790155	-2.638986	0.00831544	0.05880205	Alcaligenaceae	EasyDNA vs MagNAPure
2.63481049	4.11471371	1.78335384	2.30728957	0.02103868	0.05943999	Cryomorphaceae	FastDNA vs QIAStool
14.253878	-2.3206117	1.01141826	-2.2944135	0.02176676	0.05943999	Pirellulaceae	FastDNA vs QIAStool
5.3314072	-4.2593782	1.62243256	-2.6253037	0.00865717	0.05973448	[Methanomassiliicoccaceae]	FastDNA vs PowerSoil.HMP
2576.15115	0.8484523	0.34193436	2.48133089	0.01308928	0.05999253	[Paraprevotellaceae]	QIAStool vs MagNAPure
576.775751	-1.100544	0.43076515	-2.5548584	0.0106231	0.06126359	Streptococcaceae	FastDNA vs PowerSoil.HMP
31.9177507	-2.2924488	0.90657913	-2.5286804	0.01144922	0.06126359	Succinivibrionaceae	FastDNA vs PowerSoil.HMP
15.8153467	3.36640237	1.3327887	2.52583353	0.01154242	0.06126359	Moraxellaceae	FastDNA vs PowerSoil.HMP
408.703374	1.21382263	0.49751615	2.4397653	0.01469681	0.0621788	Lactobacillaceae	QIAStool vs MagNAPure
8.45632111	4.57043088	1.84612969	2.47568246	0.01329818	0.06286412	Rikenellaceae	PowerSoil.HMP vs MagNAPure
15.8153467	-2.986528	1.18870072	-2.5124305	0.01199027	0.06394811	Moraxellaceae	EasyDNA vs FastDNA
7.44045113	-2.4644294	0.82396759	-2.99093	0.00278129	0.06452599	Anaeroplasmataceae	QIAStool+BB vs MagNAPure
254.614729	0.77621196	0.33732155	2.30110398	0.02138575	0.06541525	S24-7	FastDNA vs InnuPURE
1.25729577	3.29462871	1.41071562	2.3354308	0.01952093	0.06678212	Methanobacteriaceae	InnuPURE vs QIAStool
30.2465249	-3.1037748	1.32591991	-2.3408463	0.01924008	0.06907747	Verrucomicrobiaceae	EasyDNA vs PowerSoil.HMP
1329.00016	-0.7318973	0.31460229	-2.3264208	0.01999611	0.06907747	Peptostreptococcaceae	EasyDNA vs PowerSoil.HMP
7.94956632	1.80438583	0.77397907	2.33131089	0.01973697	0.0690794	Christensenellaceae	EasyDNA vs InnuPURE
1574.16355	-0.9102305	0.38780387	-2.3471412	0.01891808	0.0693663	Lachnospiraceae	InnuPURE vs MagNAPure
30.2465249	2.8646008	1.26008035	2.27334773	0.02300523	0.06993591	Verrucomicrobiaceae	EasyDNA vs QIAStool
7308.6798	-0.5854122	0.25988525	-2.2525795	0.02428568	0.07015862	Prevotellaceae	FastDNA vs InnuPURE
8.45632111	-4.399111	1.79521351	-2.4504667	0.01426711	0.07031649	Rikenellaceae	FastDNA vs PowerSoil.HMP
8.45632111	4.29348299	1.74750758	2.45691809	0.01401346	0.07049952	Rikenellaceae	PowerSoil.HMP vs QIAStool+BB
32.5255849	3.84761504	1.60906299	2.39121468	0.01679273	0.07049952	Staphylococcaceae	PowerSoil.HMP vs QIAStool+BB
2516.24433	0.64083569	0.26815705	2.38977754	0.01685858	0.07049952	Ruminococcaceae	PowerSoil.HMP vs QIAStool+BB
7308.6798	-0.916953	0.31545518	-2.9067617	0.00365191	0.07060364	Prevotellaceae	QIAStool+BB vs MagNAPure
1019.42191	-1.0332649	0.45044532	-2.2938743	0.02179772	0.07202725	Campylobacteraceae	EasyDNA vs PowerSoil.HMP
32.5255849	-3.3654336	1.44833673	-2.3236541	0.02014404	0.07428114	Staphylococcaceae	InnuPURE vs PowerSoil.HMP
5.22647741	4.41383425	1.81274855	2.434885	0.01489652	0.07448262	Sphingobacteriaceae	EasyDNA vs FastDNA
4.2789796	4.42090287	1.86386076	2.37190619	0.01769658	0.07448975	Xanthomonadaceae	PowerSoil.HMP vs MagNAPure
14966.648	-0.8528435	0.36244882	-2.3530039	0.01862244	0.07448975	Clostridiaceae	PowerSoil.HMP vs MagNAPure
42.1528852	2.1770014	0.86093835	2.52863796	0.01145061	0.07557401	Bifidobacteriaceae	EasyDNA vs MagNAPure
31.9177507	-1.9115858	0.86810273	-2.2020272	0.02766339	0.07571032	Succinivibrionaceae	FastDNA vs InnuPURE
71.0660634	1.18925034	0.49362875	2.40919991	0.01598754	0.07887186	Alcaligenaceae	QIAStool vs QIAStool+BB
32.5255849	3.0928854	1.44133799	2.14584325	0.03188549	0.08290226	Staphylococcaceae	FastDNA vs InnuPURE
254.614729	-0.7523977	0.32981434	-2.2812764	0.0225321	0.08369064	S24-7	PowerSoil.HMP vs MagNAPure
408.703374	1.16600506	0.49279223	2.36611902	0.01797566	0.08459136	Lactobacillaceae	EasyDNA vs FastDNA
88.4317884	-1.591849	0.67867494	-2.345525	0.0190003	0.0874014	Coriobacteriaceae	FastDNA vs PowerSoil.HMP
367.01539	-1.3611864	0.59325355	-2.294443	0.02176507	0.08842059	Spirochaetaceae	FastDNA vs MagNAPure
88.4317884	-1.211262	0.54246601	-2.232881	0.0255568	0.08859689	Coriobacteriaceae	PowerSoil.HMP vs MagNAPure
17.2832685	-1.4788953	0.66253337	-2.2321824	0.02560291	0.08865293	Desulfovibrionaceae	InnuPURE vs PowerSoil.HMP
8.45632111	-4.1252908	1.86593498	-2.2108438	0.02704666	0.08865293	Rikenellaceae	InnuPURE vs PowerSoil.HMP
408.703374	-1.5156269	0.63804615	-2.375419	0.01752904	0.09122372	Lactobacillaceae	FastDNA vs QIAStool+BB
7.94956632	2.22772913	0.93886337	2.37279374	0.01765412	0.09122372	Christensenellaceae	FastDNA vs QIAStool+BB
86.5992792	2.35593043	1.00429657	2.34585133	0.01898368	0.09122372	Porphyromonadaceae	FastDNA vs QIAStool+BB
2.60378858	4.2092934	1.79779891	2.34135941	0.01921366	0.09122372	Rhodocyclaceae	FastDNA vs QIAStool+BB
2516.24433	0.61507077	0.26609194	2.31149716	0.02080541	0.09122372	Ruminococcaceae	FastDNA vs QIAStool+BB
2.29903269	3.13747594	1.40445963	2.23393815	0.02548714	0.09345117	Deferribacteraceae	PowerSoil.HMP vs QIAStool
4.16403335	4.04801207	1.8168604	2.22802592	0.02587879	0.09345117	Carnobacteriaceae	PowerSoil.HMP vs QIAStool
62.3051996	-1.4507519	0.631896	-2.2958713	0.02168324	0.09350897	Enterobacteriaceae	FastDNA vs PowerSoil.HMP
29.6083625	-1.0028885	0.45984104	-2.180946	0.02918741	0.09485908	[Mogibacteriaceae]	PowerSoil.HMP vs MagNAPure
7.94956632	-1.8663171	0.86386794	-2.1604194	0.03074022	0.09545646	Christensenellaceae	InnuPURE vs PowerSoil.HMP
14.253878	-2.4827691	1.09644696	-2.2643768	0.02355095	0.09558915	Pirellulaceae	FastDNA vs PowerSoil.HMP
5.3314072	-3.2764048	1.58511548	-2.0669818	0.03873587	0.09591739	[Methanomassiliicoccaceae]	FastDNA vs InnuPURE
35.1041071	-0.9596602	0.42884473	-2.2377801	0.0252354	0.0967357	Peptococcaceae	PowerSoil.HMP vs QIAStool+BB
2.95003546	4.04538074	1.85721321	2.17819942	0.02939119	0.09745501	Actinomycetaceae	EasyDNA vs InnuPURE
5.22647741	3.87561089	1.79473927	2.15942836	0.03081695	0.09758701	Sphingobacteriaceae	EasyDNA vs PowerSoil.HMP
32.5255849	3.57506683	1.60327104	2.22985804	0.02575687	0.09766893	Staphylococcaceae	FastDNA vs QIAStool+BB
5.3314072	-3.6680621	1.66483055	-2.2032645	0.02757611	0.09766893	[Methanomassiliicoccaceae]	FastDNA vs QIAStool+BB
4.00044772	3.823021	1.74418415	2.19186775	0.02838906	0.09766893	Cytophagaceae	FastDNA vs QIAStool+BB
2.63481049	4.11626382	1.88669558	2.18173184	0.02912933	0.09766893	Cryomorphaceae	FastDNA vs QIAStool+BB
31.9177507	1.3344078	0.57886073	2.3052312	0.02115363	0.09783553	Succinivibrionaceae	QIAStool vs QIAStool+BB
2.95003546	4.1628328	1.90223045	2.18839563	0.0286408	0.09798167	Actinomycetaceae	PowerSoil.HMP vs QIAStool
30.2465249	-3.1673216	1.41854368	-2.2327981	0.02556226	0.09798868	Verrucomicrobiaceae	FastDNA vs PowerSoil.HMP
2576.15115	-0.7722792	0.37881863	-2.0386515	0.04148482	0.09805502	[Paraprevotellaceae]	FastDNA vs InnuPURE
20.723165	-2.6807638	1.24693501	-2.1498826	0.03156451	0.09811055	Pseudomonadaceae	InnuPURE vs QIAStool
7.94956632	-1.557632	0.72508445	-2.1482077	0.03169726	0.09811055	Christensenellaceae	InnuPURE vs QIAStool

7.46995934	-1.5690896	0.72723812	-2.1576009	0.03095888	0.09843754	RFP12	InnuPURE vs MagNAPure
22.9687795	1.19410946	0.55614757	2.14710901	0.0317846	0.09843754	Helicobacteraceae	InnuPURE vs MagNAPure
9.6383629	-1.384477	0.64643194	-2.1417212	0.03221592	0.09843754	Fibrobacteraceae	InnuPURE vs MagNAPure
9.6383629	-1.8427971	0.80591343	-2.2865943	0.02221951	0.09875339	Fibrobacteraceae	EasyDNA vs FastDNA
5.3314072	-3.5558936	1.59551674	-2.2286783	0.02583532	0.09878209	[Methanomassiliicoccaceae]	FastDNA vs MagNAPure
1574.16355	-0.86689	0.35975205	-2.4096875	0.01596619	0.0987908	Lachnospiraceae	EasyDNA vs MagNAPure
7308.6798	0.55606923	0.26154925	2.12605939	0.03349831	0.09882003	Prevotellaceae	InnuPURE vs PowerSoil.HMP
30.2465249	3.46560811	1.53285987	2.26087732	0.02376685	0.09902856	Verrucomicrobiaceae	EasyDNA vs QIAStool+BB
14966.648	0.71562954	0.32014118	2.23535608	0.02539398	0.09976207	Clostridiaceae	QIAStool vs MagNAPure
75.3501469	-1.6556432	0.80348783	-2.0605704	0.03934405	0.09978224	Mycoplasmataceae	FastDNA vs QIAStool
30.2465249	2.801054	1.35940485	2.06050023	0.03935074	0.09978224	Verrucomicrobiaceae	FastDNA vs QIAStool
8.41300461	2.92327159	1.38042248	2.11766444	0.0342035	0.09997945	Neisseriaceae	EasyDNA vs QIAStool

Table S2C. Differential abundance of families - Hospital sewage microbial community

baseMean	log2FoldCha	lfcSE	stat	pvalue	padj	family	compare
649.223144	6.82534658	0.30988145	22.0256701	1.63E-107	3.12E-105	Bifidobacteriaceae	EasyDNA vs QIAStool
649.223144	5.53968468	0.33942717	16.3206873	7.03E-60	1.15E-57	Bifidobacteriaceae	EasyDNA vs PowerSoil.HMP
858.112951	4.52279196	0.28850532	15.6766329	2.19E-55	3.13E-53	Legionellaceae	EasyDNA vs FastDNA
649.223144	4.65718093	0.31803068	14.6438102	1.48E-48	1.99E-46	Bifidobacteriaceae	EasyDNA vs MagNAPure
283.625111	6.76475532	0.54891922	12.323772	6.75E-35	6.44E-33	Lactobacillaceae	EasyDNA vs QIAStool
649.223144	4.37138738	0.3572889	12.2348815	2.02E-34	3.06E-32	Bifidobacteriaceae	EasyDNA vs QIAStool+BB
458.885072	4.53073551	0.37259059	12.1600912	5.07E-34	3.23E-32	Carnobacteriaceae	EasyDNA vs QIAStool
649.223144	3.79123624	0.31179797	12.1592717	5.12E-34	3.66E-32	Bifidobacteriaceae	EasyDNA vs FastDNA
858.112951	3.26216457	0.29164006	11.1855845	4.80E-29	3.91E-27	Legionellaceae	EasyDNA vs PowerSoil.HMP
283.625111	6.53180922	0.59946607	10.8960449	1.20E-27	8.13E-26	Lactobacillaceae	EasyDNA vs MagNAPure
858.112951	3.3518407	0.31881432	10.5134571	7.49E-26	5.65E-24	Legionellaceae	EasyDNA vs QIAStool+BB
858.112951	-3.2399311	0.31273297	-10.360056	3.77E-25	4.18E-23	Legionellaceae	FastDNA vs MagNAPure
505.493872	3.75708256	0.37090716	10.129442	4.09E-24	1.95E-22	Erysipelotrichaceae	EasyDNA vs QIAStool
3668.6678	4.98922885	0.49392154	10.1012579	5.45E-24	2.45E-22	Lachnospiraceae	EasyDNA vs MagNAPure
458.885072	4.14006653	0.40630321	10.1895985	2.21E-24	3.16E-22	Carnobacteriaceae	FastDNA vs QIAStool
649.223144	3.76051584	0.391297	9.6103875	7.23E-22	1.18E-19	Bifidobacteriaceae	EasyDNA vs InnuPURE
103.008126	3.09894526	0.32848091	9.43417169	3.94E-21	1.51E-19	Enterococcaceae	EasyDNA vs QIAStool
3668.6678	4.06945931	0.44648024	9.11453394	7.90E-20	2.52E-18	Lachnospiraceae	EasyDNA vs QIAStool
458.885072	-3.9036097	0.42130142	-9.2655984	1.94E-20	2.70E-18	Carnobacteriaceae	QIAStool vs QIAStool+BB
505.493872	3.74637486	0.41138597	9.10671522	8.49E-20	2.87E-18	Erysipelotrichaceae	EasyDNA vs MagNAPure
649.223144	3.03411034	0.3389894	8.9504579	3.54E-19	2.53E-17	Bifidobacteriaceae	FastDNA vs QIAStool
49.8040986	4.33239316	0.49224425	8.80130772	1.35E-18	3.69E-17	Actinomycetaceae	EasyDNA vs QIAStool
4234.46977	2.87073548	0.32448599	8.84702452	8.99E-19	4.28E-17	Ruminococcaceae	EasyDNA vs FastDNA
858.112951	2.1585116	0.25552818	8.44725466	2.98E-17	7.12E-16	Legionellaceae	EasyDNA vs QIAStool
283.625111	5.01823943	0.58931798	8.51533406	1.66E-17	9.03E-16	Lactobacillaceae	EasyDNA vs PowerSoil.HMP
2750.67146	3.61050489	0.43024624	8.39171743	4.79E-17	1.29E-15	Bacteroidaceae	EasyDNA vs MagNAPure
4234.46977	2.65205125	0.32465158	8.16891528	3.11E-16	7.00E-15	Ruminococcaceae	EasyDNA vs MagNAPure
458.885072	4.13915825	0.49523734	8.35792836	6.38E-17	8.87E-15	Carnobacteriaceae	InnuPURE vs QIAStool
858.112951	-2.3642804	0.2878549	-8.213445	2.15E-16	1.02E-14	Legionellaceae	FastDNA vs QIAStool
719.094782	2.76017849	0.34134669	8.08614399	6.16E-16	2.51E-14	Streptococcaceae	EasyDNA vs PowerSoil.HMP
719.094782	2.39430118	0.30288969	7.90486188	2.68E-15	5.69E-14	Streptococcaceae	EasyDNA vs QIAStool
103.008126	2.8436344	0.36066035	7.88452173	3.16E-15	6.09E-14	Enterococcaceae	EasyDNA vs MagNAPure
103.008126	3.01172614	0.38076559	7.90965945	2.58E-15	8.41E-14	Enterococcaceae	EasyDNA vs PowerSoil.HMP
505.493872	3.18511617	0.40804762	7.80574613	5.92E-15	1.71E-13	Erysipelotrichaceae	EasyDNA vs FastDNA
3668.6678	3.84726205	0.49296566	7.80432056	5.98E-15	1.71E-13	Lachnospiraceae	EasyDNA vs FastDNA
33.4444509	4.99601637	0.68201466	7.32537975	2.38E-13	4.55E-12	Coriobacteriaceae	EasyDNA vs QIAStool
118.339928	-2.2031982	0.29992413	-7.3458517	2.04E-13	7.31E-12	[Chthoniobacteraceae]	FastDNA vs QIAStool
649.223144	3.06483074	0.41298549	7.42115838	1.16E-13	8.07E-12	Bifidobacteriaceae	InnuPURE vs QIAStool
49.8040986	3.68571012	0.5128394	7.18687007	6.63E-13	1.90E-11	Actinomycetaceae	FastDNA vs QIAStool
3668.6678	3.52725908	0.49371146	7.14437356	9.04E-13	2.15E-11	Lachnospiraceae	EasyDNA vs PowerSoil.HMP
505.493872	2.94927376	0.41296872	7.14163966	9.22E-13	2.15E-11	Erysipelotrichaceae	EasyDNA vs PowerSoil.HMP
186.231671	-2.2374803	0.31528142	-7.0967719	1.28E-12	8.88E-11	Rhizobiaceae	QIAStool vs QIAStool+BB
719.094782	2.55887653	0.36330665	7.04329673	1.88E-12	9.45E-11	Streptococcaceae	EasyDNA vs QIAStool+BB
858.112951	2.49333772	0.35533582	7.0168488	2.27E-12	1.85E-10	Legionellaceae	EasyDNA vs InnuPURE
3668.6678	3.35179711	0.49869049	6.72119718	1.80E-11	6.80E-10	Lachnospiraceae	EasyDNA vs QIAStool+BB
4234.46977	2.19156111	0.32972499	6.64663329	3.00E-11	9.06E-10	Ruminococcaceae	EasyDNA vs QIAStool+BB
993.963868	-2.9717971	0.45776516	-6.4919687	8.47E-11	1.47E-09	Opitutaceae	EasyDNA vs QIAStool
505.493872	3.34829908	0.50439697	6.63822198	3.17E-11	1.73E-09	Erysipelotrichaceae	EasyDNA vs InnuPURE
49.8040986	3.93076665	0.601821	6.53145479	6.51E-11	3.02E-09	Actinomycetaceae	InnuPURE vs QIAStool
4234.46977	1.84495753	0.29167984	6.32528303	2.53E-10	4.02E-09	Ruminococcaceae	EasyDNA vs QIAStool
649.223144	-2.4539592	0.3810699	-6.4396564	1.20E-10	5.55E-09	Bifidobacteriaceae	QIAStool vs QIAStool+BB
505.493872	2.75232367	0.43583608	6.31504315	2.70E-10	6.80E-09	Erysipelotrichaceae	EasyDNA vs QIAStool+BB
458.885072	2.49945084	0.40038248	6.24265787	4.30E-10	7.26E-09	Carnobacteriaceae	EasyDNA vs MagNAPure
458.885072	2.52065316	0.40591353	6.20982789	5.30E-10	1.02E-08	Carnobacteriaceae	EasyDNA vs PowerSoil.HMP
47.9856545	-3.2556254	0.52513758	-6.1995666	5.66E-10	1.02E-08	Bacteriovoracaceae	EasyDNA vs PowerSoil.HMP
49.8040986	3.25085928	0.52651405	6.17430685	6.65E-10	1.02E-08	Actinomycetaceae	EasyDNA vs PowerSoil.HMP
33.4444509	4.67402796	0.75777778	6.16807211	6.91E-10	1.02E-08	Coriobacteriaceae	EasyDNA vs PowerSoil.HMP
858.112951	-1.9793037	0.31561673	-6.2712254	3.58E-10	2.50E-08	Legionellaceae	PowerSoil.HMP vs MagNAPure
622.632062	-2.1934305	0.35305951	-6.2126369	5.21E-10	2.50E-08	Flavobacteriaceae	PowerSoil.HMP vs MagNAPure
283.625111	3.63327256	0.59610996	6.09497039	1.09E-09	2.61E-08	Lactobacillaceae	FastDNA vs QIAStool
141.200238	-2.2467743	0.37457141	-5.9982536	1.99E-09	2.71E-08	Chitinophagaceae	EasyDNA vs PowerSoil.HMP
649.223144	-2.1681657	0.3447053	-6.2899111	3.18E-10	6.58E-08	Bifidobacteriaceae	QIAStool vs MagNAPure
616.023371	2.17009588	0.37325392	5.8139935	6.10E-09	9.15E-08	Veillonellaceae	EasyDNA vs MagNAPure

103.008126	2.0301104	0.34927259	5.81239536	6.16E-09	1.26E-07	Enterococcaceae	FastDNA vs QIAStool
858.112951	-2.0689798	0.34079652	-6.0710121	1.27E-09	1.31E-07	Legionellaceae	QIAStool+BB vs MagNAPure
141.200238	-2.3121306	0.39864712	-5.7999431	6.63E-09	1.43E-07	Chitinophagaceae	EasyDNA vs QIAStool+BB
2750.67146	2.58911904	0.42988976	6.02275112	1.71E-09	1.77E-07	Bacteroidaceae	QIAStool vs MagNAPure
579.211848	-1.7141861	0.29702596	-5.771166	7.87E-09	1.88E-07	Cryomorphaceae	EasyDNA vs FastDNA
622.632062	-1.8973044	0.31931391	-5.9418158	2.82E-09	1.94E-07	Flavobacteriaceae	QIAStool vs MagNAPure
283.625111	-3.671693	0.63002671	-5.8278371	5.62E-09	1.95E-07	Lactobacillaceae	QIAStool vs QIAStool+BB
118.339928	1.81003807	0.31730854	5.70434721	1.17E-08	2.39E-07	[Chthoniobacteraceae]	EasyDNA vs FastDNA
639.445335	-1.9434602	0.3470908	-5.5992847	2.15E-08	2.70E-07	Marinilabiaceae	EasyDNA vs PowerSoil.HMP
47.9856545	-2.7589818	0.49172905	-5.6107766	2.01E-08	2.96E-07	Bacteriovoracaceae	EasyDNA vs QIAStool
50.9419349	-3.1533338	0.56681969	-5.5632045	2.65E-08	3.08E-07	Desulfobulbaceae	EasyDNA vs PowerSoil.HMP
4234.46977	1.80165225	0.3248057	5.54686163	2.91E-08	3.16E-07	Ruminococcaceae	EasyDNA vs PowerSoil.HMP
169.692077	-2.5020001	0.43818754	-5.7098842	1.13E-08	3.62E-07	Pirellulaceae	PowerSoil.HMP vs MagNAPure
283.625111	3.13148276	0.56384853	5.55376592	2.80E-08	5.00E-07	Lactobacillaceae	EasyDNA vs FastDNA
639.445335	-1.9819676	0.35653587	-5.558957	2.71E-08	5.12E-07	Marinilabiaceae	EasyDNA vs QIAStool+BB
47.9856545	-2.8371254	0.51608043	-5.4974481	3.85E-08	6.12E-07	Bacteriovoracaceae	EasyDNA vs FastDNA
186.231671	-1.6588195	0.29104376	-5.6995534	1.20E-08	6.22E-07	Rhizobiaceae	QIAStool vs MagNAPure
49.8040986	2.6607802	0.48751569	5.45783502	4.82E-08	6.51E-07	Actinomycetaceae	EasyDNA vs MagNAPure
141.200238	-2.0144185	0.3687622	-5.4626491	4.69E-08	6.71E-07	Chitinophagaceae	EasyDNA vs FastDNA
33.4444509	3.7400853	0.68725107	5.44209458	5.27E-08	6.85E-07	Coriobacteriaceae	EasyDNA vs FastDNA
4234.46977	2.2738468	0.40301486	5.64209171	1.68E-08	6.85E-07	Ruminococcaceae	EasyDNA vs InnuPURE
50.9419349	-2.9788598	0.55810891	-5.3374166	9.43E-08	1.12E-06	Desulfobulbaceae	EasyDNA vs FastDNA
224.417038	-2.2900472	0.43302783	-5.2884528	1.23E-07	1.26E-06	Caulobacteraceae	EasyDNA vs PowerSoil.HMP
579.211848	-1.5784754	0.29952912	-5.2698562	1.37E-07	1.31E-06	Cryomorphaceae	EasyDNA vs PowerSoil.HMP
60.913589	1.80194128	0.33612104	5.36098928	8.28E-08	1.48E-06	SB-1	FastDNA vs QIAStool
50.9419349	-3.4975916	0.64107451	-5.455827	4.87E-08	1.59E-06	Desulfobulbaceae	EasyDNA vs InnuPURE
264.350163	1.67550628	0.29505857	5.67855486	1.36E-08	1.73E-06	Leptotrichiaceae	PowerSoil.HMP vs QIAStool
24.9235631	-2.9250852	0.56407379	-5.1856428	2.15E-07	1.95E-06	[Bryobacteraceae]	EasyDNA vs PowerSoil.HMP
2750.67146	2.31054863	0.43619881	5.29700806	1.18E-07	1.98E-06	Bacteroidaceae	EasyDNA vs QIAStool+BB
50.9419349	-2.9345835	0.56106287	-5.2304005	1.69E-07	2.08E-06	Desulfobulbaceae	EasyDNA vs MagNAPure
2750.67146	2.23082024	0.42928279	5.1966217	2.03E-07	2.23E-06	Bacteroidaceae	EasyDNA vs FastDNA
164.684355	2.43591952	0.46885051	5.19551427	2.04E-07	2.30E-06	Clostridiaceae	EasyDNA vs MagNAPure
616.023371	1.91438373	0.37167225	5.15073087	2.59E-07	2.65E-06	Veillonellaceae	EasyDNA vs FastDNA
579.211848	-1.5289396	0.29774329	-5.1350935	2.82E-07	2.93E-06	Cryomorphaceae	EasyDNA vs MagNAPure
264.350163	-1.5725743	0.29211855	-5.3833429	7.31E-08	3.03E-06	Leptotrichiaceae	QIAStool vs MagNAPure
47.9856545	-2.8959851	0.56014488	-5.1700644	2.34E-07	3.48E-06	Bacteriovoracaceae	EasyDNA vs QIAStool+BB
283.625111	3.09306229	0.60001044	5.15501411	2.54E-07	3.48E-06	Lactobacillaceae	EasyDNA vs QIAStool+BB
104.663517	2.01013043	0.39723097	5.06035681	4.18E-07	4.04E-06	Rikenellaceae	EasyDNA vs MagNAPure
719.094782	2.18960446	0.41686817	5.2525105	1.50E-07	4.08E-06	Streptococcaceae	EasyDNA vs InnuPURE
49.8040986	-2.9975018	0.57075775	-5.2517935	1.51E-07	4.19E-06	Actinomycetaceae	QIAStool vs QIAStool+BB
28.0267224	3.26712548	0.65449599	4.99181896	5.98E-07	5.38E-06	Succinivibrionaceae	EasyDNA vs MagNAPure
3668.6678	3.086453	0.5976439	5.16436792	2.41E-07	5.62E-06	Lachnospiraceae	EasyDNA vs InnuPURE
22.1599751	3.76303745	0.74421723	5.05636971	4.27E-07	5.83E-06	Sanguibacteraceae	EasyDNA vs QIAStool
283.625111	3.40032645	0.64218587	5.29492571	1.19E-07	6.61E-06	Lactobacillaceae	FastDNA vs MagNAPure
186.231671	1.46311188	0.28954379	5.05316272	4.35E-07	6.90E-06	Rhizobiaceae	FastDNA vs QIAStool
131.739477	-1.6538134	0.33613801	-4.9200427	8.65E-07	7.30E-06	Hyphomicrobiaceae	EasyDNA vs MagNAPure
719.094782	1.64683881	0.33485216	4.91810712	8.74E-07	8.33E-06	Streptococcaceae	EasyDNA vs FastDNA
639.445335	-1.6956173	0.34566547	-4.9053709	9.33E-07	8.33E-06	Marinilabiaceae	EasyDNA vs FastDNA
7.94601326	4.25743862	0.86954011	4.8961958	9.77E-07	1.24E-05	Micrococcaceae	EasyDNA vs QIAStool
354.810526	-1.2016891	0.25020884	-4.8027444	1.57E-06	1.25E-05	Pseudomonadaceae	EasyDNA vs FastDNA
20.5932418	3.40730412	0.70969866	4.80105753	1.58E-06	1.25E-05	[Mogibacteriaceae]	EasyDNA vs FastDNA
47.9856545	-2.9519041	0.59210402	-4.9854486	6.18E-07	1.26E-05	Bacteriovoracaceae	EasyDNA vs InnuPURE
33.4444509	3.30560527	0.69235087	4.77446541	1.80E-06	1.43E-05	Coriobacteriaceae	EasyDNA vs MagNAPure
31.145957	4.67590454	0.90414752	5.17161683	2.32E-07	1.47E-05	Aerococcaceae	PowerSoil.HMP vs QIAStool
1110.97909	-0.8552071	0.17647177	-4.8461411	1.26E-06	1.50E-05	Verrucomicrobiaceae	EasyDNA vs QIAStool
33.4444509	3.74329586	0.7734292	4.83986884	1.30E-06	1.63E-05	Coriobacteriaceae	EasyDNA vs QIAStool+BB
283.625111	3.43874693	0.67321308	5.10796215	3.26E-07	1.68E-05	Lactobacillaceae	QIAStool+BB vs MagNAPure
616.023371	2.26022292	0.4609869	4.90300899	9.44E-07	1.71E-05	Veillonellaceae	EasyDNA vs InnuPURE
92.6279814	2.25174145	0.47401585	4.75035053	2.03E-06	1.74E-05	Oxalobacteraceae	EasyDNA vs PowerSoil.HMP
858.112951	-2.0294542	0.37889716	-5.356214	8.50E-08	1.76E-05	Legionellaceae	FastDNA vs InnuPURE
44.0121162	-2.6519931	0.53414421	-4.9649384	6.87E-07	1.84E-05	Microbacteriaceae	QIAStool vs MagNAPure
458.885072	-2.0312847	0.410162	-4.9523961	7.33E-07	1.84E-05	Carnobacteriaceae	QIAStool vs MagNAPure
28.0267224	3.13232418	0.63285923	4.9494801	7.44E-07	1.84E-05	Succinivibrionaceae	QIAStool vs MagNAPure
104.663517	1.89306701	0.38356181	4.93549398	7.99E-07	1.84E-05	Rikenellaceae	QIAStool vs MagNAPure
283.625111	3.52238694	0.70298392	5.01062233	5.43E-07	1.89E-05	Lactobacillaceae	InnuPURE vs QIAStool

993.963868	2.43312399	0.49693261	4.89628565	9.77E-07	2.02E-05	Opitutaceae	QIAStool vs MagNAPure
224.417038	-2.0089553	0.43129404	-4.6579715	3.19E-06	2.40E-05	Caulobacteraceae	EasyDNA vs MagNAPure
2750.67146	2.5329193	0.52617828	4.81380436	1.48E-06	2.41E-05	Bacteroidaceae	EasyDNA vs InnuPURE
283.625111	3.24236838	0.67710254	4.78859282	1.68E-06	2.49E-05	Lactobacillaceae	EasyDNA vs InnuPURE
579.211848	-1.7395483	0.3646933	-4.7698939	1.84E-06	2.50E-05	Cryomorphaceae	EasyDNA vs InnuPURE
616.023371	1.78559065	0.37061859	4.81786587	1.45E-06	2.73E-05	Veillonellaceae	QIAStool vs MagNAPure
127.559639	-1.7610135	0.38087354	-4.6236173	3.77E-06	3.07E-05	Bradyrhizobiaceae	EasyDNA vs PowerSoil.HMP
354.810526	-1.1755292	0.25502204	-4.6095201	4.04E-06	3.13E-05	Pseudomonadaceae	EasyDNA vs PowerSoil.HMP
1110.97909	1.00356055	0.20789468	4.82725467	1.38E-06	3.21E-05	Verrucomicrobiaceae	QIAStool vs QIAStool+BB
458.885072	2.12998418	0.43685713	4.8756997	1.08E-06	3.63E-05	Carnobacteriaceae	FastDNA vs PowerSoil.HMP
103.008126	1.94289127	0.39867209	4.87340682	1.10E-06	3.63E-05	Enterococcaceae	FastDNA vs PowerSoil.HMP
649.223144	1.74844843	0.36606848	4.77628779	1.79E-06	3.63E-05	Bifidobacteriaceae	FastDNA vs PowerSoil.HMP
49.8040986	2.60417624	0.54562554	4.77282692	1.82E-06	3.63E-05	Actinomycetaceae	FastDNA vs PowerSoil.HMP
4830.98886	1.39637929	0.30634779	4.55815037	5.16E-06	3.82E-05	Moraxellaceae	EasyDNA vs PowerSoil.HMP
458.885072	2.10878186	0.43175866	4.8841681	1.04E-06	3.84E-05	Carnobacteriaceae	FastDNA vs MagNAPure
186.231671	1.44657273	0.29780928	4.8573796	1.19E-06	4.18E-05	Rhizobiaceae	PowerSoil.HMP vs QIAStool
458.885072	2.01008235	0.41555158	4.83714281	1.32E-06	4.18E-05	Carnobacteriaceae	PowerSoil.HMP vs QIAStool
127.559639	1.70915508	0.35669596	4.79163005	1.65E-06	4.20E-05	Bradyrhizobiaceae	PowerSoil.HMP vs QIAStool
858.112951	1.28286089	0.28337494	4.5270795	5.98E-06	4.25E-05	Legionellaceae	EasyDNA vs MagNAPure
113.494552	1.61419048	0.36198295	4.45929977	8.22E-06	5.55E-05	Alcaligenaceae	EasyDNA vs MagNAPure
2750.67146	1.91527189	0.42988963	4.45526421	8.38E-06	5.94E-05	Bacteroidaceae	EasyDNA vs PowerSoil.HMP
103.008126	1.77479953	0.37958812	4.67559296	2.93E-06	6.60E-05	Enterococcaceae	FastDNA vs MagNAPure
21.4381793	4.3110629	0.92258237	4.67282169	2.97E-06	6.60E-05	Turicibacteraceae	FastDNA vs MagNAPure
186.231671	-1.4894853	0.32863304	-4.5323662	5.83E-06	6.77E-05	Rhizobiaceae	EasyDNA vs QIAStool+BB
719.094782	1.45997524	0.33529292	4.35432771	1.33E-05	8.58E-05	Streptococcaceae	EasyDNA vs MagNAPure
24.9235631	-2.4240291	0.5584747	-4.3404456	1.42E-05	8.73E-05	[Bryobacteraceae]	EasyDNA vs MagNAPure
141.200238	-1.9858992	0.44217029	-4.4912543	7.08E-06	8.88E-05	Chitinophagaceae	EasyDNA vs InnuPURE
60.913589	-1.5556429	0.34130474	-4.5579295	5.17E-06	8.91E-05	SB-1	QIAStool vs MagNAPure
21.4381793	3.83550551	0.84989348	4.51292495	6.39E-06	9.14E-05	Turicibacteraceae	FastDNA vs QIAStool
47.9856545	-2.257859	0.52469926	-4.3031488	1.68E-05	9.88E-05	Bacteriovoracaceae	EasyDNA vs MagNAPure
129.664767	-1.7354308	0.40486896	-4.2864011	1.82E-05	0.00010214	Sphingomonadaceae	EasyDNA vs MagNAPure
186.231671	1.65979847	0.36004846	4.60993081	4.03E-06	0.00010414	Rhizobiaceae	InnuPURE vs QIAStool
7.94601326	4.39122021	0.9573039	4.5870702	4.50E-06	0.00010414	Micrococcaceae	InnuPURE vs QIAStool
103.008126	1.74349936	0.39463833	4.41796766	9.96E-06	0.00010746	Enterococcaceae	EasyDNA vs QIAStool+BB
24.9235631	-2.6423032	0.60049176	-4.4002323	1.08E-05	0.00010886	[Bryobacteraceae]	EasyDNA vs QIAStool+BB
639.445335	-1.4715753	0.34627638	-4.2497132	2.14E-05	0.00011558	Marinilabiaceae	EasyDNA vs MagNAPure
59.9667273	1.93226679	0.42523105	4.54403979	5.52E-06	0.00011681	Phyllobacteriaceae	PowerSoil.HMP vs QIAStool
21.4381793	4.27081182	0.9437759	4.52523934	6.03E-06	0.00011979	Turicibacteraceae	InnuPURE vs QIAStool
622.632062	-1.3757081	0.32541716	-4.2275217	2.36E-05	0.00012268	Flavobacteriaceae	EasyDNA vs MagNAPure
79.5100271	-1.7761899	0.42104528	-4.2185246	2.46E-05	0.00012295	Bdellovibrionaceae	EasyDNA vs MagNAPure
616.023371	1.72314588	0.3956667	4.35504397	1.33E-05	0.00012556	Veillonellaceae	EasyDNA vs QIAStool+BB
269.467613	3.83469555	0.91517191	4.19013688	2.79E-05	0.00013441	[Paraprevotellaceae]	EasyDNA vs MagNAPure
224.417038	-1.9574683	0.45287758	-4.3222901	1.54E-05	0.00013716	Caulobacteraceae	EasyDNA vs QIAStool+BB
622.632062	1.57003075	0.35300582	4.44760591	8.68E-06	0.00013893	Flavobacteriaceae	FastDNA vs PowerSoil.HMP
579.211848	-1.3487004	0.31530459	-4.2774525	1.89E-05	0.00015859	Cryomorphaceae	EasyDNA vs QIAStool+BB
17.3475351	-3.0168408	0.68543834	-4.4013307	1.08E-05	0.00017131	Isosphaeraceae	QIAStool vs MagNAPure
642.395629	-1.4644737	0.33909709	-4.3187444	1.57E-05	0.0001763	Rhodocyclaceae	EasyDNA vs QIAStool
21.4381793	4.36627385	0.95987712	4.54878417	5.40E-06	0.00018525	Turicibacteraceae	QIAStool+BB vs MagNAPure
736.495903	-3.1494341	0.72564066	-4.3402117	1.42E-05	0.00018979	Enterobacteriaceae	FastDNA vs PowerSoil.HMP
354.810526	-0.9826337	0.22992472	-4.273719	1.92E-05	0.00020399	Pseudomonadaceae	EasyDNA vs QIAStool
310.195483	-1.5002553	0.3530494	-4.2494202	2.14E-05	0.00021545	Aeromonadaceae	EasyDNA vs QIAStool
127.559639	-1.5335396	0.37748821	-4.0624835	4.86E-05	0.00022602	Bradyrhizobiaceae	EasyDNA vs MagNAPure
736.495903	3.2147007	0.72609436	4.42738697	9.54E-06	0.00022892	Enterobacteriaceae	PowerSoil.HMP vs MagNAPure
21.4381793	3.65889131	0.90779156	4.03054124	5.56E-05	0.00025042	Turicibacteraceae	EasyDNA vs MagNAPure
22.1599751	3.8088383	0.87850368	4.33559743	1.45E-05	0.00025257	Sanguibacteraceae	InnuPURE vs QIAStool
28.7248963	-2.4613767	0.5983952	-4.1132963	3.90E-05	0.0002592	Acetobacteraceae	EasyDNA vs PowerSoil.HMP
20.5932418	3.06459248	0.74584242	4.10890073	3.98E-05	0.0002592	[Mogibacteriaceae]	EasyDNA vs PowerSoil.HMP
21.4381793	-3.8907165	0.8934125	-4.3548937	1.33E-05	0.00026436	Turicibacteraceae	QIAStool vs QIAStool+BB
20.5932418	2.73509037	0.65453561	4.17867316	2.93E-05	0.00028002	[Mogibacteriaceae]	EasyDNA vs QIAStool
169.692077	-1.6566863	0.38767879	-4.2733476	1.93E-05	0.00028471	Pirellulaceae	QIAStool vs MagNAPure
19.2945448	-2.3378592	0.57446007	-4.0696636	4.71E-05	0.00029516	BA008	EasyDNA vs PowerSoil.HMP
59.9667273	-1.9028345	0.45384514	-4.1926957	2.76E-05	0.00031504	Phyllobacteriaceae	FastDNA vs PowerSoil.HMP
24.9235631	2.00013401	0.46676422	4.28510563	1.83E-05	0.00033138	[Bryobacteraceae]	PowerSoil.HMP vs QIAStool
118.159531	-1.6325049	0.41315264	-3.9513361	7.77E-05	0.00033844	Rhodobacteraceae	EasyDNA vs MagNAPure
31.145957	4.0364622	0.94315101	4.27976235	1.87E-05	0.00035922	Aerococcaceae	PowerSoil.HMP vs MagNAPure

127.559639	-1.4816812	0.35306201	-4.19666	2.71E-05	0.00036159	Bradyrhizobiaceae	QIAStool vs MagNAPure
118.339928	1.24831573	0.29795829	4.18956536	2.79E-05	0.00036159	[Chthoniobacteraceae]	QIAStool vs MagNAPure
59.9667273	-1.8333257	0.46049263	-3.981227	6.86E-05	0.0004139	Phyllobacteriaceae	EasyDNA vs PowerSoil.HMP
639.445335	1.40828004	0.33538657	4.19897568	2.68E-05	0.00042565	Marinilabiaceae	PowerSoil.HMP vs QIAStool
79.5100271	-1.6859989	0.41893896	-4.0244501	5.71E-05	0.00042982	Bdellovibrionaceae	EasyDNA vs FastDNA
24.9235631	-2.221681	0.55402879	-4.0100462	6.07E-05	0.00043405	[Bryobacteraceae]	EasyDNA vs FastDNA
118.339928	-1.589293	0.37966561	-4.1860337	2.84E-05	0.00043842	[Chthoniobacteraceae]	InnuPURE vs QIAStool
616.023371	-1.5298785	0.3690248	-4.1457335	3.39E-05	0.00044035	Veillonellaceae	FastDNA vs QIAStool
6.92597984	-3.2118216	0.79077444	-4.0616154	4.87E-05	0.00044325	[Chromatiaceae]	EasyDNA vs QIAStool
354.810526	-1.2624747	0.30675563	-4.1155713	3.86E-05	0.00044967	Pseudomonadaceae	EasyDNA vs InnuPURE
21.4381793	4.74636921	1.00321669	4.73115056	2.23E-06	0.00046213	Turicibacteraceae	InnuPURE vs MagNAPure
639.445335	-1.4467874	0.34517174	-4.1915002	2.77E-05	0.00048149	Marinilabiaceae	QIAStool vs QIAStool+BB
17.3475351	-3.0488699	0.72528896	-4.2036624	2.63E-05	0.00048587	Isosphaeraceae	FastDNA vs MagNAPure
91.6048239	-1.2060837	0.30202592	-3.9933119	6.52E-05	0.00053723	Halomonadaceae	EasyDNA vs QIAStool
50.9419349	-2.1166588	0.53101195	-3.986085	6.72E-05	0.00053723	Desulfobulbaceae	EasyDNA vs QIAStool
141.200238	-1.3720459	0.34431013	-3.9849128	6.75E-05	0.00053723	Chitinophagaceae	EasyDNA vs QIAStool
70.3986464	1.43164248	0.37367651	3.83123484	0.0001275	0.0005379	Desulfovibrionaceae	EasyDNA vs MagNAPure
103.008126	1.77265924	0.43241403	4.0994489	4.14E-05	0.00055112	Enterococcaceae	InnuPURE vs QIAStool
616.023371	-1.8757177	0.45889686	-4.0874494	4.36E-05	0.00055112	Veillonellaceae	InnuPURE vs QIAStool
11.2601759	3.51749155	0.88819118	3.9602865	7.49E-05	0.00057193	Peptococcaceae	EasyDNA vs QIAStool
310.195483	-1.5679178	0.3821482	-4.1029052	4.08E-05	0.00057573	Aeromonadaceae	PowerSoil.HMP vs QIAStool
50.9419349	-2.4153589	0.61074665	-3.9547641	7.66E-05	0.00060885	Desulfobulbaceae	EasyDNA vs QIAStool+BB
118.339928	1.41559349	0.34454956	4.10853378	3.98E-05	0.00061497	[Chthoniobacteraceae]	QIAStool vs QIAStool+BB
182.91656	-1.4289407	0.34903446	-4.0939816	4.24E-05	0.00067844	Planctomycetaceae	PowerSoil.HMP vs MagNAPure
458.885072	1.87232506	0.44582209	4.19971356	2.67E-05	0.00068818	Carnobacteriaceae	QIAStool+BB vs MagNAPure
593.953594	-0.8557847	0.22798878	-3.7536264	0.00017429	0.00071302	Cytophagaceae	EasyDNA vs MagNAPure
622.632062	1.27390462	0.3192542	3.99025176	6.60E-05	0.00073743	Flavobacteriaceae	FastDNA vs QIAStool
12.2684488	-2.4781182	0.62161852	-3.9865578	6.70E-05	0.00073743	Synergistaceae	FastDNA vs QIAStool
164.684355	1.63111762	0.42086767	3.87560684	0.00010636	0.00078133	Clostridiaceae	EasyDNA vs QIAStool
17.3475351	-2.7235825	0.73173203	-3.7221038	0.00019757	0.00078447	Isosphaeraceae	EasyDNA vs MagNAPure
12.0832988	2.86029733	0.72533982	3.94338935	8.03E-05	0.0008206	Microthrixaceae	FastDNA vs QIAStool
858.112951	-1.2606274	0.32020896	-3.9368897	8.25E-05	0.00082545	Legionellaceae	FastDNA vs PowerSoil.HMP
49.8040986	2.84923277	0.62918338	4.52846159	5.94E-06	0.00084963	Actinomycetaceae	InnuPURE vs PowerSoil.HMP
141.200238	-1.3748839	0.3732346	-3.6836989	0.00022987	0.00087939	Chitinophagaceae	EasyDNA vs MagNAPure
20.5932418	2.60659271	0.70858012	3.67861394	0.0002345	0.00087939	[Mogibacteriaceae]	EasyDNA vs MagNAPure
283.625111	3.28944084	0.74068442	4.44108277	8.95E-06	0.0009264	Lactobacillaceae	InnuPURE vs MagNAPure
21.4381793	3.18333391	0.83254469	3.82361927	0.00013151	0.00093029	Turicibacteraceae	EasyDNA vs QIAStool
129.664767	-1.5315971	0.40361056	-3.79474	0.0001478	0.00100644	Sphingomonadaceae	EasyDNA vs FastDNA
131.739477	-1.2097072	0.30846328	-3.921722	8.79E-05	0.00107054	Hyphomicrobiaceae	QIAStool vs MagNAPure
736.495903	-2.5211072	0.67768495	-3.7201758	0.00019908	0.00115895	Enterobacteriaceae	EasyDNA vs PowerSoil.HMP
105.619852	-1.7219182	0.47856033	-3.5981215	0.00032052	0.00116948	Nitrosomonadaceae	EasyDNA vs MagNAPure
118.159531	-1.6800207	0.44427482	-3.7814899	0.00015589	0.00117699	Rhodobacteraceae	EasyDNA vs QIAStool+BB
49.8040986	2.01409717	0.50832968	3.96218684	7.43E-05	0.00117765	Actinomycetaceae	FastDNA vs MagNAPure
616.023371	1.37539476	0.37422072	3.67535705	0.00023752	0.00133501	Veillonellaceae	EasyDNA vs PowerSoil.HMP
59.9667273	-1.7905328	0.4630503	-3.8668214	0.00011026	0.00153266	Phyllobacteriaceae	QIAStool vs QIAStool+BB
4421.89202	1.4148799	0.38994132	3.62844316	0.00028514	0.00154924	Campylobacteraceae	EasyDNA vs PowerSoil.HMP
7.76499835	3.00619582	0.78949885	3.80772665	0.00014025	0.00161288	Victivallaceae	QIAStool vs MagNAPure
858.112951	-1.103653	0.29099694	-3.7926618	0.00014904	0.00168722	Legionellaceae	PowerSoil.HMP vs QIAStool
118.339928	-1.1641985	0.30830147	-3.7761692	0.00015926	0.00168722	[Chthoniobacteraceae]	PowerSoil.HMP vs QIAStool
224.417038	1.54686811	0.40966719	3.77591408	0.00015942	0.00168722	Caulobacteraceae	PowerSoil.HMP vs QIAStool
31.145957	3.18626251	0.86955576	3.66424172	0.00024807	0.00169221	Aerococcaceae	EasyDNA vs QIAStool
264.350163	1.08538354	0.29166878	3.72128808	0.00019821	0.00188959	Leptotrichiaceae	FastDNA vs QIAStool
91.6048239	-1.1722866	0.3237836	-3.6205868	0.00029394	0.00191058	Halomonadaceae	EasyDNA vs FastDNA
7.94601326	3.25719295	0.87981198	3.70214661	0.00021378	0.00191069	Micrococcaceae	FastDNA vs QIAStool
15.0781299	2.03353532	0.58767751	3.46029119	0.00053959	0.00191697	[Odoribacteraceae]	EasyDNA vs MagNAPure
38.1375375	1.96739339	0.52466333	3.74982069	0.00017696	0.00192794	Procabacteriaceae	QIAStool vs MagNAPure
12.0832988	2.60554261	0.71947574	3.62144612	0.00029296	0.0019295	Microthrixaceae	EasyDNA vs QIAStool
269.467613	3.28592868	0.91384685	3.59571046	0.00032351	0.00201137	[Paraprevotellaceae]	EasyDNA vs FastDNA
59.9667273	-1.5618619	0.41967538	-3.7215952	0.00019797	0.00204897	Phyllobacteriaceae	QIAStool vs MagNAPure
92.6279814	1.60546842	0.44860355	3.57881343	0.00034516	0.00205114	Oxalobacteraceae	EasyDNA vs FastDNA
60.913589	-1.2906483	0.36164555	-3.5688212	0.00035859	0.00205114	SB-1	EasyDNA vs FastDNA
8.22875632	-3.0323745	0.85554848	-3.5443632	0.00039356	0.00206938	Solibacteraceae	EasyDNA vs PowerSoil.HMP
458.885072	2.1290759	0.52006231	4.09388616	4.24E-05	0.00207036	Carnobacteriaceae	InnuPURE vs PowerSoil.HMP
649.223144	1.77916883	0.43517387	4.08840914	4.34E-05	0.00207036	Bifidobacteriaceae	InnuPURE vs PowerSoil.HMP
118.159531	-1.4258087	0.38539195	-3.6996327	0.00021591	0.00212827	Rhodobacteraceae	QIAStool vs MagNAPure

3.24919787	3.6188219	1.00940315	3.58511056	0.00033694	0.00214516	Leuconostocaceae	EasyDNA vs QIAStool
33.4444509	2.98208837	0.80192515	3.71866173	0.00020028	0.00217639	Coriobacteriaceae	EasyDNA vs InnuPURE
1110.97909	-0.706397	0.19141459	-3.6904028	0.0002239	0.00218732	Verrucomicrobiaceae	PowerSoil.HMP vs QIAStool
858.112951	1.1933291	0.31822856	3.74991205	0.0001769	0.00223533	Legionellaceae	QIAStool vs QIAStool+BB
1181.58837	3.09655277	0.88052293	3.51672018	0.00043691	0.00240303	Prevotellaceae	EasyDNA vs FastDNA
28.7248963	-2.008188	0.59242969	-3.389749	0.00069957	0.00242158	Acetobacteraceae	EasyDNA vs MagNAPure
19.2945448	-1.9141579	0.56688273	-3.3766382	0.00073378	0.00246687	BA008	EasyDNA vs MagNAPure
92.6279814	1.52494408	0.4523832	3.37091228	0.0007492	0.00246687	Oxalobacteraceae	EasyDNA vs MagNAPure
169.692077	2.05994287	0.51874697	3.97099742	7.16E-05	0.00255871	Pirellulaceae	InnuPURE vs PowerSoil.HMP
354.810526	-0.98635	0.27628461	-3.5700506	0.00035691	0.00256637	Pseudomonadaceae	EasyDNA vs QIAStool+BB
129.664767	-1.3164872	0.3730486	-3.5289965	0.00041714	0.00257011	Sphingomonadaceae	EasyDNA vs QIAStool
4.87640195	3.65458882	1.09184921	3.34715525	0.00081645	0.00262432	Corynebacteriaceae	EasyDNA vs MagNAPure
22.1599751	-3.0574814	0.83143843	-3.6773395	0.00023568	0.00272995	Sanguibacteraceae	QIAStool vs QIAStool+BB
44.0121162	2.3469519	0.63926998	3.67130004	0.00024132	0.00279529	Microbacteriaceae	InnuPURE vs QIAStool
2.31382502	3.84858947	1.07433352	3.5823042	0.00034058	0.00286485	Nocardiaceae	FastDNA vs QIAStool
458.885072	2.10787358	0.51588	4.08597657	4.39E-05	0.00302853	Carnobacteriaceae	InnuPURE vs MagNAPure
17.3475351	-2.7947582	0.75672129	-3.6932464	0.00022141	0.00303647	Isosphaeraceae	PowerSoil.HMP vs MagNAPure
47.3908296	1.37466258	0.40163928	3.42262981	0.00062018	0.00328468	Christensenellaceae	EasyDNA vs FastDNA
169.692077	-1.7496979	0.46383396	-3.7722504	0.00016178	0.0033327	Pirellulaceae	QIAStool+BB vs MagNAPure
113.494552	1.2476785	0.34930574	3.57188087	0.00035443	0.00333483	Alcaligenaceae	QIAStool vs MagNAPure
113.494552	1.20497493	0.35380394	3.40577026	0.00065978	0.00336957	Alcaligenaceae	EasyDNA vs FastDNA
12.2684488	2.29895523	0.64642084	3.55643735	0.00037592	0.00338326	Synergistaceae	QIAStool vs MagNAPure
28.7248963	-1.9922099	0.56229317	-3.5430093	0.00039559	0.00351634	Acetobacteraceae	FastDNA vs PowerSoil.HMP
2750.67146	1.695233	0.46819787	3.6207619	0.00029374	0.00352484	Bacteroidaceae	PowerSoil.HMP vs MagNAPure
118.159531	-1.7715548	0.49520976	-3.5773826	0.00034705	0.00353559	Rhodobacteraceae	EasyDNA vs InnuPURE
92.6279814	-1.6411801	0.46414377	-3.5359305	0.00040634	0.00354978	Oxalobacteraceae	PowerSoil.HMP vs QIAStool
649.223144	1.28566191	0.36445274	3.52765052	0.00041927	0.00354978	Bifidobacteriaceae	PowerSoil.HMP vs QIAStool
44.0121162	-2.1137016	0.58933766	-3.5865715	0.00033505	0.00357391	Microbacteriaceae	PowerSoil.HMP vs MagNAPure
354.810526	-0.8207072	0.25232262	-3.2526104	0.0011435	0.00359006	Pseudomonadaceae	EasyDNA vs MagNAPure
12.0832988	-2.8162903	0.78971557	-3.5662084	0.00036218	0.00387258	Microthrixaceae	QIAStool vs QIAStool+BB
169.692077	1.41344996	0.42000085	3.36535026	0.00076447	0.003894	Pirellulaceae	EasyDNA vs PowerSoil.HMP
1110.97909	-0.8492203	0.23853992	-3.5600764	0.00037075	0.00396414	Verrucomicrobiaceae	InnuPURE vs QIAStool
118.159531	-1.5107086	0.41671211	-3.6253053	0.00028862	0.00400461	Rhodobacteraceae	FastDNA vs MagNAPure
639.445335	1.16043718	0.33390826	3.47531735	0.00051025	0.00403097	Marinilabiaceae	FastDNA vs QIAStool
993.963868	-1.7178466	0.49615794	-3.4622979	0.00053558	0.00403097	Opitutaceae	FastDNA vs QIAStool
59.9667273	-1.4629208	0.45541119	-3.2123076	0.00131673	0.00403998	Phyllobacteriaceae	EasyDNA vs MagNAPure
7.4067071	-2.8464888	0.85314609	-3.3364612	0.00084852	0.00419119	Desulfobacteraceae	EasyDNA vs PowerSoil.HMP
719.094782	-1.3002032	0.36982603	-3.5157159	0.00043857	0.00421027	Streptococcaceae	PowerSoil.HMP vs MagNAPure
118.159531	-1.4733244	0.41877181	-3.5182035	0.00043448	0.00431376	Rhodobacteraceae	QIAStool vs QIAStool+BB
593.953594	-0.7654102	0.23067053	-3.3181968	0.00090601	0.0043435	Cytophagaceae	EasyDNA vs PowerSoil.HMP
59.9667273	-1.6915917	0.49533902	-3.4150181	0.00063778	0.00437747	Phyllobacteriaceae	EasyDNA vs QIAStool+BB
70.3986464	1.2061253	0.36301817	3.32249295	0.00089217	0.00439932	Desulfovibrionaceae	EasyDNA vs FastDNA
269.467613	3.16345108	0.93031323	3.40041501	0.00067284	0.00441732	[Paraprevotellaceae]	EasyDNA vs QIAStool+BB
1181.58837	2.792938	0.8806047	3.17161376	0.00151594	0.00454783	Prevotellaceae	EasyDNA vs MagNAPure
182.91656	-1.3835089	0.37942483	-3.6463319	0.00026601	0.00456651	Planctomycetaceae	QIAStool+BB vs MagNAPure
4421.89202	1.229723	0.38951484	3.15706329	0.00159367	0.00467707	Campylobacteraceae	EasyDNA vs MagNAPure
19.2945448	-2.0575467	0.61259008	-3.3587659	0.00078291	0.00476931	BA008	EasyDNA vs QIAStool+BB
92.6279814	1.70551314	0.50813645	3.35640779	0.00078962	0.00476931	Oxalobacteraceae	EasyDNA vs QIAStool+BB
15.0781299	2.00704774	0.61163899	3.28142545	0.00103284	0.00481008	[Odoribacteraceae]	EasyDNA vs PowerSoil.HMP
254.528648	-1.3406432	0.42677372	-3.1413445	0.00168174	0.00483053	Xanthomonadaceae	EasyDNA vs MagNAPure
129.664767	-1.4706229	0.44044394	-3.3389559	0.00084094	0.00488391	Sphingomonadaceae	EasyDNA vs QIAStool+BB
60.913589	1.45888204	0.41924116	3.4798159	0.00050176	0.00498174	SB-1	InnuPURE vs QIAStool
11.2601759	2.86575309	0.92393381	3.10168657	0.00192422	0.00541186	Peptococcaceae	EasyDNA vs MagNAPure
458.885072	-1.8935274	0.45074517	-4.2008822	2.66E-05	0.00550365	Carnobacteriaceae	PowerSoil.HMP vs QIAStool+BB
736.495903	-3.0921527	0.82941526	-3.7281117	0.00019292	0.00551751	Enterobacteriaceae	InnuPURE vs PowerSoil.HMP
129.664767	-1.3324584	0.41218724	-3.2326533	0.00122646	0.00555315	Sphingomonadaceae	EasyDNA vs PowerSoil.HMP
118.159531	-1.3509387	0.4195755	-3.2197749	0.00128291	0.00565175	Rhodobacteraceae	EasyDNA vs PowerSoil.HMP
11.2601759	3.04335101	0.94850206	3.20858662	0.00133389	0.00572169	Peptococcaceae	EasyDNA vs PowerSoil.HMP
616.023371	1.33864065	0.39319285	3.40453967	0.00066276	0.00607517	Veillonellaceae	QIAStool vs QIAStool+BB
103.008126	-1.3554459	0.40137289	-3.377024	0.00073275	0.00607517	Enterococcaceae	QIAStool vs QIAStool+BB
24.9235631	-1.717352	0.51140949	-3.3580762	0.00078487	0.00607517	[Bryobacteraceae]	QIAStool vs QIAStool+BB
60.913589	-1.3029449	0.38807828	-3.3574279	0.00078671	0.00607517	SB-1	QIAStool vs QIAStool+BB
22.5747955	1.6881564	0.55662937	3.0328195	0.0024228	0.00667507	Peptostreptococcaceae	EasyDNA vs MagNAPure
7.4067071	-2.6173944	0.80329827	-3.2583096	0.00112078	0.00668966	Desulfobacteraceae	EasyDNA vs QIAStool
12.2684488	2.11983061	0.66428024	3.19116918	0.00141698	0.00675428	Synergistaceae	EasyDNA vs FastDNA

104.663517	1.42534693	0.4244879	3.35780346	0.00078564	0.00685653	Rikenellaceae	PowerSoil.HMP vs MagNAPure
20.5932418	2.78844857	0.82723718	3.37079697	0.00074951	0.00718649	[Mogibacteriaceae]	EasyDNA vs InnuPURE
993.963868	-2.017559	0.60189132	-3.3520321	0.00080221	0.00743379	Opitutaceae	InnuPURE vs QIAStool
186.231671	-0.9108244	0.30550845	-2.9813394	0.0028699	0.00768192	Rhizobiaceae	EasyDNA vs MagNAPure
1110.97909	-0.5821481	0.19548775	-2.9779262	0.00290206	0.00768192	Verrucomicrobiaceae	EasyDNA vs MagNAPure
59.9667273	-1.5324296	0.44867974	-3.4154197	0.00063684	0.00785434	Phyllobacteriaceae	FastDNA vs MagNAPure
579.211848	-0.8687005	0.27121003	-3.2030546	0.00135978	0.00787025	Cryomorphaceae	EasyDNA vs QIAStool
103.008126	1.06883487	0.34143186	3.13044852	0.0017454	0.00805134	Enterococcaceae	EasyDNA vs FastDNA
118.159531	1.5648585	0.47276645	3.31000327	0.00093295	0.00810499	Rhodobacteraceae	InnuPURE vs QIAStool
8.22875632	2.50314686	0.76230268	3.28366528	0.00102467	0.00813328	Solibacteraceae	PowerSoil.HMP vs QIAStool
20.5932418	2.47178571	0.77677805	3.18210037	0.00146211	0.00817699	[Mogibacteriaceae]	EasyDNA vs QIAStool+BB
49.8040986	2.25915369	0.59809529	3.7772471	0.00015857	0.00820607	Actinomycetaceae	InnuPURE vs MagNAPure
103.008126	1.68544012	0.47255029	3.56668943	0.00036152	0.00858467	Enterococcaceae	InnuPURE vs PowerSoil.HMP
622.632062	1.49623869	0.42421902	3.52704296	0.00042023	0.00858467	Flavobacteriaceae	InnuPURE vs PowerSoil.HMP
6.92597984	-2.9939275	0.90591845	-3.3048533	0.00095026	0.00860514	[Chromatiaceae]	EasyDNA vs InnuPURE
7.94601326	2.36493345	0.80553543	2.93585279	0.00332632	0.00863564	Micrococcaceae	EasyDNA vs MagNAPure
28.0267224	2.247816	0.69396685	3.23908267	0.00119915	0.00959318	Succinivibrionaceae	PowerSoil.HMP vs MagNAPure
24.9235631	-1.4990779	0.45978042	-3.2604213	0.00111247	0.00959504	[Bryobacteraceae]	QIAStool vs MagNAPure
4421.89202	1.22665838	0.39306924	3.12071835	0.0018041	0.00972928	Campylobacteraceae	EasyDNA vs QIAStool+BB
736.495903	2.16738284	0.67437254	3.21392511	0.00130934	0.00978153	Enterobacteriaceae	PowerSoil.HMP vs QIAStool
19.2945448	1.55104302	0.48513414	3.19714258	0.00138796	0.00979285	BA008	PowerSoil.HMP vs QIAStool
269.467613	2.67877514	0.85846322	3.12043087	0.00180587	0.01014472	[Paraprevotellaceae]	EasyDNA vs QIAStool
254.528648	-1.2938909	0.42934953	-3.0136073	0.00258162	0.01078984	Xanthomonadaceae	EasyDNA vs PowerSoil.HMP
4421.89202	-1.2253129	0.38948425	-3.1459884	0.00165527	0.01106414	Campylobacteraceae	PowerSoil.HMP vs QIAStool
60.913589	-1.04435	0.3664465	-2.8499385	0.00437277	0.01113818	SB-1	EasyDNA vs MagNAPure
4234.46977	-1.025778	0.3242894	-3.163156	0.00156069	0.01115891	Ruminococcaceae	FastDNA vs QIAStool
17.3475351	-2.6763124	0.799256	-3.3485046	0.00081249	0.01146109	Isosphaeraceae	QIAStool+BB vs MagNAPure
622.632062	-1.2115734	0.36458966	-3.3231151	0.00089018	0.01146109	Flavobacteriaceae	QIAStool+BB vs MagNAPure
264.350163	0.85382433	0.2783669	3.0672624	0.00216029	0.01178902	Leptotrichiaceae	EasyDNA vs QIAStool
12.2684488	1.94066769	0.68710829	2.82439859	0.00473694	0.01184236	Synergistaceae	EasyDNA vs MagNAPure
993.963868	-1.503144	0.50502245	-2.9763905	0.00291663	0.01186219	Opitutaceae	EasyDNA vs PowerSoil.HMP
131.739477	-1.0249579	0.34517249	-2.969408	0.00298374	0.01186219	Hyphomicrobiaceae	EasyDNA vs PowerSoil.HMP
4.87640195	3.28815114	1.07742791	3.05185257	0.00227434	0.01206663	Corynebacteriaceae	EasyDNA vs QIAStool
31.145957	2.54682017	0.91162549	2.79371319	0.00521067	0.01278982	Aerococcaceae	EasyDNA vs MagNAPure
1110.97909	-0.7305016	0.22421632	-3.2580214	0.00112192	0.01283975	Verrucomicrobiaceae	QIAStool+BB vs MagNAPure
113.494552	1.40369726	0.44319404	3.16722954	0.00153899	0.0132029	Alcaligenaceae	EasyDNA vs InnuPURE
60.913589	1.09927041	0.35735416	3.07613715	0.00209701	0.01331604	SB-1	PowerSoil.HMP vs QIAStool
4.06716246	2.70023017	0.85880434	3.14417387	0.00166556	0.01379087	Elusimicrobiaceae	QIAStool vs MagNAPure
49.8040986	-1.671613	0.53708995	-3.1123519	0.00185603	0.0139388	Actinomycetaceae	QIAStool vs MagNAPure
224.417038	-1.2657762	0.40782434	-3.103729	0.00191098	0.0139388	Caulobacteraceae	QIAStool vs MagNAPure
182.91656	-0.9604176	0.30999369	-3.0981843	0.0019471	0.0139388	Planctomycetaceae	QIAStool vs MagNAPure
858.112951	-0.8756507	0.28271224	-3.0973216	0.00195278	0.0139388	Legionellaceae	QIAStool vs MagNAPure
4830.98886	0.90318919	0.30567964	2.954692	0.00312981	0.01398635	Moraxellaceae	EasyDNA vs FastDNA
127.559639	-1.1983362	0.38295763	-3.1291612	0.00175306	0.01402449	Bradyrhizobiaceae	FastDNA vs PowerSoil.HMP
4.81963649	-2.5920036	0.86507369	-2.9962807	0.00273295	0.01410792	auto67_4W	EasyDNA vs QIAStool
3.21882027	-2.9945197	1.02979292	-2.9078853	0.00363882	0.01412208	Geobacteraceae	EasyDNA vs PowerSoil.HMP
103.008126	1.32628602	0.42621318	3.11179028	0.00185957	0.01422317	Enterococcaceae	EasyDNA vs InnuPURE
269.467613	3.07998123	0.99275227	3.10246708	0.00191915	0.01422317	[Paraprevotellaceae]	EasyDNA vs InnuPURE
28.7248963	-2.1158352	0.68200314	-3.1023834	0.00191969	0.01422317	Acetobacteraceae	EasyDNA vs InnuPURE
12.0832988	2.60257042	0.83588608	3.11354678	0.00184853	0.01511447	Microthrixaceae	InnuPURE vs QIAStool
15.0781299	1.90584145	0.64043532	2.9758531	0.00292175	0.01521324	[Odoribacteraceae]	EasyDNA vs QIAStool+BB
7.94601326	-2.9156223	0.9471339	-3.0783634	0.00208141	0.01522715	Micrococcaceae	QIAStool vs QIAStool+BB
118.339928	-1.0389997	0.34156999	-3.0418353	0.00235141	0.01531368	[Chthoniobacteraceae]	FastDNA vs PowerSoil.HMP
4234.46977	-1.0690832	0.35423431	-3.0180115	0.00254439	0.01531368	Ruminococcaceae	FastDNA vs PowerSoil.HMP
719.094782	1.11333967	0.36942897	3.01367723	0.00258102	0.01531368	Streptococcaceae	FastDNA vs PowerSoil.HMP
169.692077	1.31061062	0.43885856	2.98640779	0.00282276	0.01531368	Pirellulaceae	FastDNA vs PowerSoil.HMP
283.625111	1.88675667	0.63288731	2.98118896	0.00287132	0.01531368	Lactobacillaceae	FastDNA vs PowerSoil.HMP
91.6048239	-0.9688326	0.3376377	-2.8694444	0.00411194	0.0153584	Halomonadaceae	EasyDNA vs PowerSoil.HMP
269.467613	2.62362002	0.91515855	2.86684753	0.00414583	0.0153584	[Paraprevotellaceae]	EasyDNA vs PowerSoil.HMP
169.692077	-1.0885501	0.39949647	-2.7248053	0.00643394	0.01551039	Pirellulaceae	EasyDNA vs MagNAPure
118.339928	0.85515562	0.31545607	2.71085484	0.006711	0.01589447	[Chthoniobacteraceae]	EasyDNA vs MagNAPure
4.27355164	-2.8506251	0.93680851	-3.0429112	0.00234301	0.01628395	Eubacteriaceae	QIAStool vs QIAStool+BB
21.5427213	2.62816676	0.89319412	2.94243625	0.00325641	0.01636774	Spirochaetaceae	EasyDNA vs QIAStool
59.9667273	1.56849566	0.51094835	3.0697734	0.00214221	0.01654264	Phyllobacteriaceae	InnuPURE vs QIAStool
118.339928	1.19613294	0.39335639	3.04083768	0.00235921	0.01671962	[Chthoniobacteraceae]	EasyDNA vs InnuPURE

224.417038	-1.3016651	0.44367212	-2.9338446	0.00334792	0.01673959	Caulobacteraceae	FastDNA vs PowerSoil.HMP
993.963868	-1.499755	0.51224519	-2.9278069	0.00341362	0.01679999	Opitutaceae	EasyDNA vs QIAStool+BB
131.739477	-1.0909518	0.37302612	-2.9245989	0.003449	0.01679999	Hyphomicrobiaceae	EasyDNA vs QIAStool+BB
21.5427213	2.53561288	0.94480086	2.68375379	0.00728007	0.01690336	Spirochaetaceae	EasyDNA vs MagNAPure
333.941631	1.06355661	0.39701881	2.67885697	0.00738739	0.01690336	Porphyromonadaceae	EasyDNA vs MagNAPure
28.7345598	-1.6199105	0.55646094	-2.9110947	0.00360165	0.01699528	Parachlamydiaceae	EasyDNA vs QIAStool+BB
21.4381793	2.87852569	0.94506216	3.04585858	0.00232017	0.01713356	Turicibacteraceae	PowerSoil.HMP vs MagNAPure
118.159531	-1.2291424	0.42307595	-2.9052522	0.00366957	0.01726858	Rhodobacteraceae	FastDNA vs PowerSoil.HMP
104.663517	1.26962895	0.43903252	2.89187904	0.00382945	0.01732243	Rikenellaceae	EasyDNA vs QIAStool+BB
21.5427213	2.85758764	0.99453991	2.87327597	0.00406239	0.01732243	Spirochaetaceae	EasyDNA vs QIAStool+BB
1181.58837	2.53355579	0.88411161	2.86565152	0.00416152	0.01732243	Prevotellaceae	EasyDNA vs QIAStool+BB
355.031722	1.13809798	0.39794486	2.85993889	0.00423723	0.01732243	[Weeksellaceae]	EasyDNA vs QIAStool+BB
113.494552	1.15165021	0.40396852	2.85084148	0.00436037	0.01732243	Alcaligenaceae	EasyDNA vs QIAStool+BB
28.7248963	-1.8378156	0.64637612	-2.8432603	0.00446546	0.01732243	Acetobacteraceae	EasyDNA vs QIAStool+BB
118.339928	1.02243338	0.35967603	2.84265084	0.00447401	0.01732243	[Chthoniobacteraceae]	EasyDNA vs QIAStool+BB
7.4067071	-2.533754	0.89397879	-2.834244	0.00459342	0.01734018	Desulfobacteraceae	EasyDNA vs QIAStool+BB
4.81963649	-2.5721622	0.89514869	-2.8734468	0.00406019	0.01759417	auto67_4W	EasyDNA vs FastDNA
38.1375375	-1.588819	0.53677015	-2.9599616	0.00307677	0.0176333	Procabacteriaceae	PowerSoil.HMP vs QIAStool
993.963868	-1.4686531	0.49715551	-2.9541121	0.0031357	0.0176333	Opitutaceae	PowerSoil.HMP vs QIAStool
377.356263	0.66481791	0.22547835	2.94847773	0.00319343	0.0176333	Sphingobacteriaceae	PowerSoil.HMP vs QIAStool
70.3986464	1.07418554	0.3564197	3.01382203	0.00257979	0.01780056	Desulfovibrionaceae	QIAStool vs MagNAPure
4830.98886	0.86879822	0.30910597	2.8106808	0.00494368	0.01820721	Moraxellaceae	EasyDNA vs QIAStool+BB
622.632062	1.20011256	0.39698082	3.02309962	0.002502	0.0183041	Flavobacteriaceae	InnuPURE vs QIAStool
22.1599751	2.34056949	0.78040891	2.99915784	0.00270727	0.01833074	Sanguibacteraceae	FastDNA vs QIAStool
579.211848	0.84548558	0.2831535	2.98596192	0.00282688	0.01833074	Cryomorphaceae	FastDNA vs QIAStool
310.195483	-1.1140138	0.37470056	-2.9730774	0.0029483	0.01833074	Aeromonadaceae	FastDNA vs QIAStool
21.5427213	2.67648334	0.94093314	2.84449896	0.00444813	0.01841406	Spirochaetaceae	EasyDNA vs FastDNA
3.24919787	2.81122058	0.98975752	2.84031241	0.00450694	0.01841406	Leuconostocaceae	EasyDNA vs FastDNA
118.159531	1.14424238	0.39230985	2.91668023	0.00353778	0.01872077	Rhodobacteraceae	PowerSoil.HMP vs QIAStool
1181.58837	2.37174212	0.82321907	2.88105827	0.00396342	0.01884447	Prevotellaceae	EasyDNA vs QIAStool
4830.98886	0.79076118	0.27480994	2.87748395	0.0040086	0.01884447	Moraxellaceae	EasyDNA vs QIAStool
4.27355164	2.54518735	0.8853998	2.87461931	0.00404515	0.01884447	Eubacteriaceae	EasyDNA vs QIAStool
113.494552	1.15379451	0.38639387	2.98605802	0.00282599	0.01937822	Alcaligenaceae	PowerSoil.HMP vs MagNAPure
92.6279814	1.64386434	0.5519235	2.97842786	0.00289731	0.01967758	Oxalobacteraceae	EasyDNA vs InnuPURE
18.7347412	1.74564306	0.58736763	2.97197698	0.00295889	0.01975774	[Barnesiellaceae]	QIAStool vs MagNAPure
24.9235631	-1.9126891	0.64531773	-2.9639494	0.00303718	0.01980243	[Bryobacteraceae]	EasyDNA vs InnuPURE
243.261282	-1.0090776	0.33828228	-2.9829456	0.00285489	0.01984147	GZKB119	InnuPURE vs QIAStool
12.0832988	1.9612567	0.7511158	2.61112429	0.00902451	0.02030515	Microthrixaceae	EasyDNA vs MagNAPure
2750.67146	1.28916277	0.43584769	2.95782861	0.00309814	0.02050676	Bacteroidaceae	QIAStool vs QIAStool+BB
243.261282	-0.7295655	0.25759568	-2.8322118	0.00462272	0.02102238	GZKB119	EasyDNA vs QIAStool
79.5100271	-1.191318	0.43277484	-2.7527432	0.00590982	0.0214067	Bdellovibrionaceae	EasyDNA vs PowerSoil.HMP
19.2945448	-1.5596526	0.56244073	-2.7730079	0.00555408	0.02193946	BA008	EasyDNA vs FastDNA
11.2601759	2.50254376	0.90615756	2.76170929	0.00574997	0.02193946	Peptococcaceae	EasyDNA vs FastDNA
593.953594	-0.62696	0.22739104	-2.7571889	0.00583007	0.02193946	Cytophagaceae	EasyDNA vs FastDNA
10.9576795	-2.0973714	0.76485713	-2.7421741	0.0061034	0.02194317	Acholeplasmataceae	EasyDNA vs QIAStool+BB
593.953594	-0.5824492	0.20721534	-2.8108403	0.00494123	0.02194826	Cytophagaceae	EasyDNA vs QIAStool
993.963868	1.47204213	0.50450751	2.91778038	0.00352533	0.02227365	Opitutaceae	QIAStool vs QIAStool+BB
10.9576795	-1.8987571	0.68029491	-2.7910793	0.00525326	0.02280392	Acholeplasmataceae	EasyDNA vs QIAStool
7.4067071	-2.284038	0.8363462	-2.7309719	0.00631478	0.02315421	Desulfobacteraceae	EasyDNA vs FastDNA
1110.97909	-0.5394896	0.18836632	-2.8640448	0.00418269	0.02338949	Verrucomicrobiaceae	FastDNA vs QIAStool
377.356263	0.62991124	0.22044344	2.85747327	0.00427029	0.02338949	Sphingobacteriaceae	FastDNA vs QIAStool
24.9235631	1.29672979	0.45420932	2.85491676	0.00430481	0.02338949	[Bryobacteraceae]	FastDNA vs QIAStool
31.145957	2.57510178	0.90456212	2.84679374	0.0044162	0.02338949	Aerococcaceae	FastDNA vs QIAStool
12.9936921	-1.8506044	0.72523789	-2.5517205	0.01071925	0.02372292	Hyphomonadaceae	EasyDNA vs MagNAPure
355.031722	0.99703315	0.36955369	2.69793854	0.00697703	0.02431227	[Weeksellaceae]	EasyDNA vs PowerSoil.HMP
264.350163	-0.8216819	0.30473801	-2.6963553	0.00701029	0.02431227	Leptotrichiaceae	EasyDNA vs PowerSoil.HMP
2750.67146	-1.2094344	0.42892542	-2.8196845	0.00480709	0.02455049	Bacteroidaceae	FastDNA vs QIAStool
283.625111	1.74651589	0.61997486	2.81707535	0.00484632	0.02461928	Lactobacillaceae	PowerSoil.HMP vs QIAStool
79.5100271	-1.2567499	0.46625307	-2.6954243	0.00702991	0.02468641	Bdellovibrionaceae	EasyDNA vs QIAStool+BB
3668.6678	1.63743174	0.54128315	3.02509274	0.00248557	0.02560136	Lachnospiraceae	QIAStool+BB vs MagNAPure
44.0121162	1.43218661	0.52197913	2.74376222	0.00607395	0.02578055	Microbacteriaceae	EasyDNA vs QIAStool
1756.14884	-0.8875915	0.35309787	-2.5137266	0.0119463	0.02592108	Neisseriaceae	EasyDNA vs MagNAPure
18.7347412	1.55956336	0.62150923	2.50931647	0.01209651	0.02592108	[Barnesiellaceae]	EasyDNA vs MagNAPure
2750.67146	-1.5115334	0.52589418	-2.8742159	0.00405032	0.02611934	Bacteroidaceae	InnuPURE vs QIAStool
4.27355164	2.76832398	0.97008355	2.85369643	0.00432138	0.02611934	Eubacteriaceae	InnuPURE vs QIAStool

17.3475351	2.27655242	0.79776644	2.85365781	0.00432191	0.02611934	Isosphaeraceae	InnuPURE vs QIAStool
38.1375375	-1.4159276	0.50824048	-2.7859402	0.00533727	0.02631827	Procabacteriaceae	FastDNA vs QIAStool
2.3093169	-2.8715958	1.08020362	-2.6583838	0.00785164	0.02694541	Myxococcaceae	EasyDNA vs QIAStool+BB
2.31382502	3.1273455	1.10587857	2.82792847	0.00468503	0.02713412	Nocardiaceae	InnuPURE vs QIAStool
254.528648	-1.1861816	0.44926195	-2.6402895	0.00828352	0.02779582	Xanthomonadaceae	EasyDNA vs QIAStool+BB
8.22875632	-2.3772037	0.9049355	-2.6269316	0.00861586	0.02828251	Solibacteraceae	EasyDNA vs QIAStool+BB
4.06716246	-2.6505323	0.9462686	-2.8010358	0.00509389	0.02832201	Elusimicrobiaceae	InnuPURE vs QIAStool
224.417038	-1.2142892	0.43070396	-2.8193127	0.00481266	0.02908521	Caulobacteraceae	QIAStool vs QIAStool+BB
8.97993102	-1.9720958	0.74525118	-2.6462163	0.00813978	0.0290997	Sediment-4	EasyDNA vs FastDNA
1.55959833	3.04990549	1.10011358	2.77235509	0.00556523	0.02914922	Dermatophilaceae	InnuPURE vs QIAStool
31.145957	2.73793794	0.98959174	2.76673484	0.00566208	0.02914922	Aerococcaceae	InnuPURE vs QIAStool
164.684355	2.00560381	0.59200326	3.38782561	0.00070449	0.0291659	Clostridiaceae	InnuPURE vs MagNAPure
21.4381793	2.4029683	0.87615781	2.74262042	0.00609511	0.02977227	Turicibacteraceae	PowerSoil.HMP vs QIAStool
22.1599751	-2.2288796	0.7867898	-2.8328781	0.0046131	0.02984097	Sanguibacteraceae	QIAStool vs MagNAPure
11.4855136	-1.8652956	0.66110538	-2.82148	0.00478026	0.02998528	Gemmatimonadaceae	QIAStool vs MagNAPure
7.76499835	2.08136474	0.84932062	2.4506231	0.01426092	0.03008163	Victivallaceae	EasyDNA vs MagNAPure
20.0435737	-1.4557469	0.56093764	-2.5952027	0.00945351	0.03037193	Saprospiraceae	EasyDNA vs QIAStool+BB
11.2601759	2.81611955	0.99962466	2.81717696	0.00484478	0.03037306	Peptococcaceae	EasyDNA vs InnuPURE
103.008126	1.51734838	0.45684108	3.32139215	0.0008957	0.03090151	Enterococcaceae	InnuPURE vs MagNAPure
639.445335	-0.9363952	0.33454193	-2.7990367	0.00512553	0.03114536	Marinilabiaceae	QIAStool vs MagNAPure
719.094782	-0.9343259	0.33484933	-2.7902876	0.00526612	0.03114536	Streptococcaceae	QIAStool vs MagNAPure
4.06716246	2.44686122	0.880977	2.77744052	0.00547889	0.03173189	Elusimicrobiaceae	QIAStool vs QIAStool+BB
858.112951	-1.2104768	0.37506448	-3.2273833	0.00124928	0.03314836	Legionellaceae	InnuPURE vs MagNAPure
1.55959833	3.53407623	1.09747746	3.22018115	0.0012811	0.03314836	Dermatophilaceae	InnuPURE vs MagNAPure
3.21882027	-2.7128444	1.06112987	-2.5565621	0.01057122	0.03325529	Geobacteraceae	EasyDNA vs QIAStool+BB
12.9936921	-1.8610075	0.71942811	-2.5867873	0.00968754	0.03378824	Hyphomonadaceae	EasyDNA vs FastDNA
49.8040986	1.33489132	0.52490846	2.54309356	0.01098758	0.03385969	Actinomycetaceae	EasyDNA vs QIAStool+BB
164.684355	1.20633872	0.46822066	2.57643208	0.00998258	0.03389918	Clostridiaceae	EasyDNA vs PowerSoil.HMP
2.61442095	-2.3742008	0.98904022	-2.4005099	0.01637225	0.0340039	Ellin515	EasyDNA vs MagNAPure
2750.67146	1.37968465	0.46764596	2.95027598	0.0031749	0.03433796	Bacteroidaceae	FastDNA vs MagNAPure
12.0832988	2.21601142	0.75663079	2.92878833	0.00340286	0.03433796	Microthrixaceae	FastDNA vs MagNAPure
2750.67146	1.02138586	0.38739727	2.63653347	0.00837579	0.03446271	Bacteroidaceae	EasyDNA vs QIAStool
2.90378317	-2.6542909	1.00834595	-2.6323217	0.00848035	0.03446271	Shewanellaceae	EasyDNA vs QIAStool
2.61442095	-2.5754714	0.93748593	-2.7472107	0.00601045	0.03456009	Ellin515	QIAStool vs MagNAPure
1.27584029	-3.0144798	1.10165276	-2.7363248	0.00621297	0.03475903	Methanobacteriaceae	QIAStool vs MagNAPure
4.18916425	2.38221387	0.99895023	2.38471727	0.01709225	0.03480958	Nocardioidaceae	EasyDNA vs MagNAPure
264.350163	-0.71875	0.30189642	-2.3807835	0.01727586	0.03480958	Leptotrichiaceae	EasyDNA vs MagNAPure
10.9576795	-2.197664	0.79886476	-2.7509837	0.00594166	0.03577349	Acholeplasmataceae	EasyDNA vs InnuPURE
1181.58837	2.64574182	0.96781367	2.73373057	0.00626213	0.03577349	Prevotellaceae	EasyDNA vs InnuPURE
59.9667273	-1.4695546	0.54001007	-2.7213466	0.00650165	0.03577349	Phyllobacteriaceae	EasyDNA vs InnuPURE
1.55959833	-2.9910789	1.10080263	-2.71718	0.00658408	0.03577349	Dermatophilaceae	EasyDNA vs InnuPURE
28.0267224	1.50756944	0.58964909	2.55672309	0.01056633	0.03597584	Succinivibrionaceae	EasyDNA vs FastDNA
616.023371	-0.9908895	0.37159274	-2.6666009	0.00766226	0.036041	Veillonellaceae	PowerSoil.HMP vs QIAStool
131.739477	-0.8564683	0.33629328	-2.5467899	0.01087189	0.03615534	Hyphomicrobiaceae	EasyDNA vs FastDNA
186.231671	0.74799507	0.28674278	2.60859252	0.00909154	0.03617677	Rhizobiaceae	EasyDNA vs QIAStool
118.339928	-0.9548824	0.33231545	-2.8734217	0.00406052	0.03636071	[Chthoniobacteraceae]	FastDNA vs MagNAPure
169.692077	-1.1913895	0.4193644	-2.8409409	0.00449807	0.03636071	Pirellulaceae	FastDNA vs MagNAPure
182.91656	-0.9532472	0.33627107	-2.8347584	0.00458604	0.03636071	Planctomycetaceae	FastDNA vs MagNAPure
91.6048239	-0.7763827	0.32992933	-2.3531788	0.01861368	0.03695363	Halomonadaceae	EasyDNA vs MagNAPure
22.1599751	1.97579153	0.77845518	2.53809287	0.01114584	0.03707698	Sanguibacteraceae	EasyDNA vs PowerSoil.HMP
28.7345598	-1.2703451	0.50442951	-2.5183798	0.01178961	0.03804106	Parachlamydiaceae	EasyDNA vs FastDNA
254.528648	-1.068488	0.42623526	-2.5068033	0.01218285	0.03804106	Xanthomonadaceae	EasyDNA vs FastDNA
6.92597984	-2.0759884	0.82940286	-2.5029916	0.01231485	0.03804106	[Chromatiaceae]	EasyDNA vs FastDNA
4421.89202	0.97253378	0.38938417	2.49762026	0.012503	0.03804106	Campylobacteraceae	EasyDNA vs FastDNA
993.963868	-1.2539505	0.50404268	-2.4877864	0.01285409	0.03829448	Opitutaceae	EasyDNA vs FastDNA
104.663517	1.15256553	0.42682061	2.70035119	0.00692663	0.03851207	Rikenellaceae	QIAStool vs QIAStool+BB
642.395629	-0.8728261	0.37490681	-2.3281148	0.01990601	0.03894653	Rhodocyclaceae	EasyDNA vs MagNAPure
6.92597984	-1.9641006	0.84680666	-2.3194203	0.02037226	0.03928935	[Chromatiaceae]	EasyDNA vs MagNAPure
3.5697885	-2.663582	0.99480591	-2.6774891	0.00741763	0.03965577	Beutenbergiaceae	QIAStool vs QIAStool+BB
8.22875632	-2.0849652	0.8464636	-2.4631481	0.0137723	0.04019264	Solibacteraceae	EasyDNA vs FastDNA
141.200238	0.87472846	0.33424873	2.61699863	0.00887067	0.04023482	Chitinophagaceae	PowerSoil.HMP vs QIAStool
4421.89202	1.04015603	0.38905723	2.67352962	0.00750576	0.04080907	Campylobacteraceae	QIAStool vs MagNAPure
4.27355164	-2.3695014	0.8889705	-2.6654444	0.00768866	0.04080907	Eubacteriaceae	QIAStool vs MagNAPure
28.0267224	1.91589962	0.72230348	2.65248565	0.00799015	0.04095456	Succinivibrionaceae	EasyDNA vs InnuPURE
639.445335	-1.1176738	0.42467418	-2.6318384	0.00849243	0.04095456	Marinilabiaceae	EasyDNA vs InnuPURE

224.417038	-1.3704338	0.52104201	-2.6301791	0.00853399	0.04095456	Caulobacteraceae	EasyDNA vs InnuPURE
104.663517	1.25209331	0.47716645	2.62401793	0.00868992	0.04095456	Rikenellaceae	EasyDNA vs InnuPURE
4830.98886	0.9966009	0.38038735	2.61996329	0.00879392	0.04095456	Moraxellaceae	EasyDNA vs InnuPURE
21.5427213	2.3806915	0.95400598	2.49546812	0.01257911	0.04100788	Spirochaetaceae	EasyDNA vs PowerSoil.HMP
4.18916425	2.4527686	0.95878924	2.5581937	0.01052175	0.04101334	Nocardioidaceae	EasyDNA vs QIAStool
3.21882027	-2.354502	1.02533773	-2.2963185	0.02165768	0.04118009	Geobacteraceae	EasyDNA vs MagNAPure
3668.6678	1.46196977	0.53675835	2.72370194	0.00645547	0.04131503	Lachnospiraceae	PowerSoil.HMP vs MagNAPure
9.46385082	-1.9959877	0.80350941	-2.4840875	0.01298839	0.04151191	Polyangiaceae	EasyDNA vs PowerSoil.HMP
28.7248963	-1.5390212	0.55585699	-2.7687358	0.00562742	0.04164294	Acetobacteraceae	FastDNA vs MagNAPure
355.031722	1.0232927	0.39395485	2.59748725	0.00939086	0.04173716	[Weeksellaceae]	FastDNA vs PowerSoil.HMP
7.76499835	2.85424593	0.91600206	3.11598202	0.00183333	0.04216669	Victivallaceae	InnuPURE vs MagNAPure
104.663517	1.13861777	0.41527505	2.74184006	0.00610961	0.04238541	Rikenellaceae	FastDNA vs MagNAPure
4421.89202	1.03709142	0.39261611	2.64148974	0.00825423	0.042494	Campylobacteraceae	QIAStool vs QIAStool+BB
622.632062	0.81772245	0.33204467	2.4626881	0.01378998	0.04266148	Flavobacteriaceae	EasyDNA vs PowerSoil.HMP
4.87640195	2.6847996	1.09159219	2.45952621	0.01391205	0.04266148	Corynebacteriaceae	EasyDNA vs PowerSoil.HMP
3.24919787	2.49848134	1.01818471	2.45385864	0.01413325	0.04266148	Leuconostocaceae	EasyDNA vs PowerSoil.HMP
28.7345598	-1.1664509	0.51264479	-2.275359	0.02288441	0.04290826	Parachlamydiaceae	EasyDNA vs MagNAPure
377.356263	-0.5824062	0.2219046	-2.6245793	0.00867561	0.04489627	Sphingobacteriaceae	QIAStool vs MagNAPure
2.31382502	-2.5964006	1.07577379	-2.4135191	0.0157993	0.045186	Nocardiaceae	EasyDNA vs FastDNA
719.094782	-1.0989013	0.39008341	-2.8170931	0.00484605	0.04537662	Streptococcaceae	QIAStool+BB vs MagNAPure
642.395629	-0.8990233	0.37439073	-2.4012969	0.01633708	0.04580788	Rhodocyclaceae	EasyDNA vs FastDNA
28.0267224	2.05253499	0.73852611	2.77923143	0.00544877	0.04586337	Succinivibrionaceae	QIAStool+BB vs MagNAPure
2750.67146	1.29995626	0.47394333	2.74285169	0.00609082	0.04586337	Bacteroidaceae	QIAStool+BB vs MagNAPure
355.031722	-1.1521059	0.42121139	-2.7352202	0.00623386	0.04586337	[Weeksellaceae]	QIAStool+BB vs MagNAPure
141.200238	-0.9400847	0.36121392	-2.6025706	0.00925278	0.04593342	Chitinophagaceae	QIAStool vs QIAStool+BB
164.684355	1.34712441	0.50064185	2.69079464	0.00712821	0.04654299	Clostridiaceae	FastDNA vs MagNAPure
59.9667273	-1.7611005	0.48922251	-3.5997946	0.00031847	0.04757822	Phyllobacteriaceae	FastDNA vs QIAStool+BB
118.159531	-1.5582244	0.44756329	-3.4815733	0.00049848	0.04757822	Rhodobacteraceae	FastDNA vs QIAStool+BB
858.112951	-1.1709513	0.34503626	-3.393705	0.00068954	0.04757822	Legionellaceae	FastDNA vs QIAStool+BB
44.0121162	-1.2198065	0.54714401	-2.2294067	0.02578686	0.04768803	Microbacteriaceae	EasyDNA vs MagNAPure
1756.14884	-1.401038	0.46013475	-3.0448428	0.00232802	0.04819001	Neisseriaceae	InnuPURE vs MagNAPure
1756.14884	-1.1234205	0.43466028	-2.5845943	0.00974937	0.04839863	Neisseriaceae	InnuPURE vs QIAStool
8.97993102	-1.6808038	0.75875734	-2.215206	0.02674593	0.04879324	Sediment-4	EasyDNA vs MagNAPure
3668.6678	1.90277585	0.63250342	3.00832499	0.00262692	0.04943387	Lachnospiraceae	InnuPURE vs MagNAPure
164.684355	1.08879511	0.4606952	2.36337412	0.01810938	0.04980079	Clostridiaceae	EasyDNA vs FastDNA
79.5100271	-0.9798214	0.380222	-2.5769718	0.00996701	0.05032123	Bdellovibrionaceae	QIAStool vs MagNAPure
186.231671	-0.7151168	0.30408292	-2.3517165	0.01868701	0.05041968	Rhizobiaceae	EasyDNA vs FastDNA
44.0121162	-1.4843431	0.56162354	-2.6429504	0.00821871	0.05068203	Microbacteriaceae	FastDNA vs MagNAPure
7.94601326	2.35096963	0.93228322	2.52173329	0.01167782	0.0511408	Micrococcaceae	PowerSoil.HMP vs QIAStool
333.941631	0.99974328	0.39058752	2.55958841	0.01047962	0.05164955	Porphyromonadaceae	QIAStool vs MagNAPure
19.2945448	-1.6538315	0.65473079	-2.5259718	0.01153788	0.05224094	BA008	EasyDNA vs InnuPURE
169.692077	1.21462904	0.47776311	2.5423249	0.01101178	0.05253157	Pirellulaceae	InnuPURE vs QIAStool
28.0267224	-1.7810983	0.703404	-2.5321129	0.01133775	0.05253157	Succinivibrionaceae	InnuPURE vs QIAStool
118.339928	0.77103841	0.32521728	2.37084086	0.01774767	0.05259764	[Chthoniobacteraceae]	EasyDNA vs PowerSoil.HMP
22.2584553	1.82334122	0.76805203	2.37398139	0.01759744	0.05314426	S24-7	EasyDNA vs QIAStool+BB
3.24919787	2.62550795	1.04618943	2.50959136	0.01208709	0.05324855	Leuconostocaceae	EasyDNA vs InnuPURE
243.261282	-0.6779435	0.28737692	-2.3590743	0.01832059	0.053326	GZKB119	EasyDNA vs PowerSoil.HMP
70.3986464	1.1324594	0.45348268	2.49724953	0.01251608	0.05368742	Desulfovibrionaceae	EasyDNA vs InnuPURE
7.94601326	1.90646899	0.81181724	2.34839678	0.01885442	0.05391704	Micrococcaceae	EasyDNA vs PowerSoil.HMP
129.664767	-1.213787	0.48816066	-2.4864499	0.01290247	0.05392572	Sphingomonadaceae	EasyDNA vs InnuPURE
12.0832988	2.17200442	0.8174185	2.65715106	0.00788041	0.05411217	Microthrixaceae	QIAStool+BB vs MagNAPure
28.0267224	1.75955604	0.67705141	2.59885144	0.00935362	0.05464486	Succinivibrionaceae	FastDNA vs MagNAPure
622.632062	-0.7523083	0.32535862	-2.3122433	0.02076428	0.05498688	Flavobacteriaceae	EasyDNA vs FastDNA
579.211848	0.70977486	0.28578343	2.48361094	0.01300578	0.05505781	Cryomorphaceae	PowerSoil.HMP vs QIAStool
11.2601759	2.2792279	0.96857952	2.35316549	0.01861435	0.05511307	Peptococcaceae	EasyDNA vs QIAStool+BB
22.9873604	-2.4720555	0.97855392	-2.5262333	0.01152929	0.05526107	Staphylococcaceae	QIAStool vs QIAStool+BB
310.195483	1.0118008	0.40293184	2.51109669	0.01203567	0.05576528	Aeromonadaceae	QIAStool vs QIAStool+BB
6.96503794	1.90349744	0.77276169	2.46323991	0.01376878	0.05640756	Piscirickettsiaceae	PowerSoil.HMP vs QIAStool
224.417038	-0.9883822	0.43171742	-2.2894192	0.02205501	0.05734302	Caulobacteraceae	EasyDNA vs FastDNA
2.31382502	-2.7483795	1.10394795	-2.4895916	0.012789	0.05734421	Nocardiaceae	QIAStool vs QIAStool+BB
182.91656	-0.6936928	0.32325492	-2.145962	0.03187601	0.05737304	Planctomycetaceae	EasyDNA vs MagNAPure
3.14112548	-2.3181345	1.08289035	-2.1406919	0.0322989	0.05737304	OM60	EasyDNA vs MagNAPure
186.231671	-0.9118034	0.37170792	-2.4530104	0.01416662	0.05772899	Rhizobiaceae	EasyDNA vs InnuPURE
5.49329045	-1.8319005	0.72941169	-2.5114767	0.01202272	0.05787682	Cyclobacteriaceae	QIAStool vs MagNAPure
8.97993102	-2.0613208	0.84378326	-2.4429505	0.01456773	0.05791562	Sediment-4	EasyDNA vs InnuPURE

12.2684488	-1.5691396	0.64325243	-2.4393838	0.01471233	0.05838958	Synergistaceae	PowerSoil.HMP vs QIAStool
104.663517	0.87151266	0.3830252	2.27534024	0.02288553	0.05843983	Rikenellaceae	EasyDNA vs FastDNA
127.559639	-0.9708623	0.3795923	-2.5576449	0.01053836	0.05848792	Bradyrhizobiaceae	FastDNA vs MagNAPure
11.4855136	1.65096216	0.68095654	2.42447508	0.01533054	0.05899933	Gemmatimonadaceae	PowerSoil.HMP vs QIAStool
4830.98886	0.64823777	0.30574015	2.12022453	0.03398711	0.0595878	Moraxellaceae	EasyDNA vs MagNAPure
4234.46977	0.80709372	0.3244551	2.48753594	0.01286315	0.06051525	Ruminococcaceae	QIAStool vs MagNAPure
6.96503794	1.86675561	0.74910667	2.49197568	0.01270347	0.06055322	Piscirickettsiaceae	FastDNA vs QIAStool
377.356263	-0.5435773	0.23659302	-2.2975206	0.02158909	0.06067279	Sphingobacteriaceae	EasyDNA vs PowerSoil.HMP
579.211848	0.87084781	0.35361388	2.46270821	0.01378921	0.06068046	Cryomorphaceae	InnuPURE vs QIAStool
50.9419349	1.38093288	0.56180151	2.45804409	0.0139696	0.06068046	Desulfobulbaceae	InnuPURE vs QIAStool
6.92597984	-1.9786325	0.86497437	-2.2875042	0.02216641	0.06123941	[Chromatiaceae]	EasyDNA vs PowerSoil.HMP
16.3205056	-1.7055823	0.7409508	-2.3018834	0.02134175	0.06197316	Methylophilaceae	EasyDNA vs QIAStool+BB
113.494552	-0.8384629	0.34080263	-2.4602596	0.01388365	0.06234019	Alcaligenaceae	FastDNA vs QIAStool
70.3986464	-0.8486684	0.34519175	-2.4585418	0.01395025	0.06234019	Desulfovibrionaceae	FastDNA vs QIAStool
355.031722	-1.0110411	0.39463636	-2.5619562	0.01040844	0.06245066	[Weeksellaceae]	PowerSoil.HMP vs MagNAPure
47.3908296	-0.9325292	0.38114769	-2.4466348	0.01441969	0.06248531	Christensenellaceae	FastDNA vs QIAStool
104.663517	-1.1350299	0.46606219	-2.4353614	0.01487692	0.06266339	Rikenellaceae	InnuPURE vs QIAStool
4.18916425	2.29762813	1.0113383	2.27186899	0.02309442	0.06273985	Nocardioidaceae	EasyDNA vs PowerSoil.HMP
28.0267224	-1.3727681	0.56486719	-2.4302494	0.01508844	0.06346019	Succinivibrionaceae	FastDNA vs QIAStool
264.350163	0.8838018	0.36532112	2.41924641	0.0155527	0.0635831	Leptotrichiaceae	InnuPURE vs QIAStool
113.494552	-1.0371853	0.43307898	-2.3949102	0.01662443	0.06456144	Alcaligenaceae	InnuPURE vs QIAStool
33.4444509	2.01392801	0.8425903	2.39016283	0.0168409	0.06456144	Coriobacteriaceae	InnuPURE vs QIAStool
3.5697885	2.44429648	1.0258444	2.38271659	0.01718542	0.06456144	Beutenbergiaceae	InnuPURE vs QIAStool
103.008126	1.10013504	0.42775185	2.57190011	0.01011421	0.06511021	Enterococcaceae	QIAStool+BB vs MagNAPure
649.223144	0.86594468	0.34641928	2.49970117	0.01242981	0.06570043	Bifidobacteriaceae	FastDNA vs MagNAPure
164.684355	1.13212163	0.49900063	2.26877795	0.02328183	0.06633126	Clostridiaceae	EasyDNA vs QIAStool+BB
28.7248963	1.22866154	0.51896204	2.36753646	0.01790696	0.06688775	Acetobacteraceae	PowerSoil.HMP vs QIAStool
186.231671	-0.6985777	0.3119467	-2.2394135	0.02512902	0.06714804	Rhizobiaceae	EasyDNA vs PowerSoil.HMP
3.21882027	-2.2403395	1.01657284	-2.203816	0.02753729	0.06722862	Geobacteraceae	EasyDNA vs FastDNA
243.261282	-0.6192531	0.28126021	-2.2017089	0.02768588	0.06722862	GZKB119	EasyDNA vs FastDNA
4.87640195	2.37711291	1.0817303	2.19750978	0.02798406	0.06722862	Corynebacteriaceae	EasyDNA vs FastDNA
377.356263	-0.5086707	0.23180568	-2.194384	0.02820781	0.06722862	Sphingobacteriaceae	EasyDNA vs FastDNA
4.27355164	2.11193339	0.88047945	2.39861747	0.0164571	0.067239	Eubacteriaceae	FastDNA vs QIAStool
264.350163	-0.7890883	0.32627137	-2.4185032	0.01558451	0.06769521	Leptotrichiaceae	QIAStool vs QIAStool+BB
17.3475351	-1.9832942	0.83527701	-2.3744149	0.01757679	0.06821467	Isosphaeraceae	EasyDNA vs InnuPURE
1181.58837	1.95342448	0.88069728	2.21804306	0.0265519	0.06980579	Prevotellaceae	EasyDNA vs PowerSoil.HMP
19.2945448	-1.2707305	0.53094712	-2.3933277	0.01669632	0.07032693	BA008	QIAStool vs QIAStool+BB
3.24919787	2.02439771	0.98885767	2.04720839	0.04063763	0.07033436	Leuconostocaceae	EasyDNA vs MagNAPure
70.3986464	0.99071862	0.39671413	2.49731114	0.01251391	0.07066679	Desulfovibrionaceae	PowerSoil.HMP vs MagNAPure
22.1599751	1.53415787	0.75524233	2.03134519	0.04221999	0.07214808	Sanguibacteraceae	EasyDNA vs MagNAPure
79.5100271	0.88963046	0.37786776	2.35434341	0.01855546	0.0724779	Bdellovibrionaceae	FastDNA vs QIAStool
5.49329045	1.69380301	0.72064323	2.3504044	0.01875302	0.0724779	Cyclobacteriaceae	FastDNA vs QIAStool
4.81963649	-2.3118932	0.98963119	-2.336116	0.0194852	0.07386249	auto67_4W	EasyDNA vs InnuPURE
2.55199853	-2.2827904	1.04339628	-2.187846	0.02868082	0.07420014	Holophagaceae	EasyDNA vs PowerSoil.HMP
182.91656	0.73524789	0.33701129	2.18167136	0.0291338	0.07420014	Planctomycetaceae	EasyDNA vs PowerSoil.HMP
22.9873604	2.14709978	0.91924374	2.33572414	0.01950562	0.07451148	Staphylococcaceae	EasyDNA vs QIAStool
642.395629	-0.8615187	0.3894487	-2.2121495	0.02695634	0.07537791	Rhodocyclaceae	EasyDNA vs QIAStool+BB
1.01494259	-2.6305311	1.10333506	-2.3841635	0.017118	0.07685828	Gemmataceae	QIAStool vs MagNAPure
310.195483	0.8934204	0.37551544	2.37918422	0.017351	0.07685828	Aeromonadaceae	QIAStool vs MagNAPure
254.528648	-0.9770092	0.41101457	-2.3770671	0.01745091	0.07685828	Xanthomonadaceae	QIAStool vs MagNAPure
4.06716246	-1.8067423	0.77930346	-2.3184066	0.02042724	0.07687091	Elusimicrobiaceae	FastDNA vs QIAStool
19.2945448	-1.1273417	0.47588079	-2.3689582	0.01783827	0.07692754	BA008	QIAStool vs MagNAPure
4.81963649	-2.1101927	0.9610752	-2.1956582	0.02811642	0.07719234	auto67_4W	EasyDNA vs QIAStool+BB
2.3093169	-2.3470892	1.01440126	-2.313768	0.02068045	0.07745031	Myxococcaceae	EasyDNA vs QIAStool
22.5747955	1.12730926	0.52924208	2.13004465	0.03316793	0.07775432	Peptostreptococcaceae	EasyDNA vs FastDNA
736.495903	2.47243917	0.73392038	3.36881115	0.00075493	0.07813539	Enterobacteriaceae	PowerSoil.HMP vs QIAStool+BB
22.9873604	1.9190924	0.96704297	1.98449547	0.04720063	0.07965107	Staphylococcaceae	EasyDNA vs MagNAPure
164.684355	1.22958079	0.50750114	2.42281388	0.01540081	0.07974548	Clostridiaceae	PowerSoil.HMP vs MagNAPure
4234.46977	0.850399	0.35438523	2.39964574	0.01641095	0.07974548	Ruminococcaceae	PowerSoil.HMP vs MagNAPure
141.200238	0.87189043	0.36402356	2.39514836	0.01661364	0.07974548	Chitinophagaceae	PowerSoil.HMP vs MagNAPure
649.223144	-0.8825037	0.37134215	-2.3765246	0.0174766	0.07989301	Bifidobacteriaceae	PowerSoil.HMP vs MagNAPure
377.356263	-0.4611657	0.23319393	-1.9776057	0.0479732	0.07995534	Sphingobacteriaceae	EasyDNA vs MagNAPure
13.1651756	-1.4992527	0.69891407	-2.1451174	0.03194346	0.08010438	Helicobacteraceae	EasyDNA vs PowerSoil.HMP
182.91656	1.18518163	0.41749716	2.83877771	0.00452867	0.08094996	Planctomycetaceae	InnuPURE vs PowerSoil.HMP
1.55959833	3.06644109	1.09689413	2.79556704	0.00518088	0.08231836	Dermatophilaceae	InnuPURE vs PowerSoil.HMP

131.739477	-0.797345	0.33199055	-2.4017101	0.01631864	0.08233494	Hyphomicrobiaceae	FastDNA vs MagNAPure
131.739477	-0.9313915	0.40909714	-2.2767002	0.02280414	0.08447896	Hyphomicrobiaceae	EasyDNA vs InnuPURE
13.1651756	-1.4567213	0.64093998	-2.272789	0.0230389	0.08462364	Helicobacteraceae	EasyDNA vs QIAStool
579.211848	-0.6602391	0.2839074	-2.3255439	0.0200429	0.08467101	Cryomorphaceae	QIAStool vs MagNAPure
92.6279814	-0.9949071	0.43808997	-2.2710109	0.02314632	0.08486984	Oxalobacteraceae	FastDNA vs QIAStool
29.0135086	-1.2323803	0.54478605	-2.2621363	0.02368898	0.08536974	Alteromonadaceae	EasyDNA vs QIAStool
243.261282	-0.6671317	0.31081555	-2.1463909	0.0318418	0.08585914	GZKB119	EasyDNA vs QIAStool+BB
38.1375375	1.08211604	0.55767402	1.94040963	0.05232993	0.08615293	Procabacteriaceae	EasyDNA vs MagNAPure
505.493872	-1.0047589	0.43784747	-2.2947692	0.02174636	0.08647001	Erysipelotrichaceae	QIAStool vs QIAStool+BB
4.18916425	-2.3294501	1.0153187	-2.2943043	0.02177303	0.08647001	Nocardioidaceae	QIAStool vs QIAStool+BB
11.4855136	-1.3573909	0.70420385	-1.927554	0.05391063	0.08768596	Gemmatimonadaceae	EasyDNA vs MagNAPure
5.49329045	-1.5087589	0.78541121	-1.9209795	0.05473429	0.08796582	Cyclobacteriaceae	EasyDNA vs MagNAPure
3.07801265	2.12104453	0.94429648	2.24616376	0.02469352	0.08827934	Caldilineaceae	FastDNA vs QIAStool
719.094782	0.74746237	0.33440796	2.23518117	0.02540546	0.08860928	Streptococcaceae	FastDNA vs QIAStool
254.528648	0.93025684	0.41369801	2.24863746	0.02453557	0.08902907	Xanthomonadaceae	PowerSoil.HMP vs QIAStool
20.0435737	-1.0503917	0.50791832	-2.0680327	0.03863694	0.08911424	Saprospiraceae	EasyDNA vs FastDNA
164.684355	1.30379789	0.53572935	2.43368761	0.01494589	0.0905545	Clostridiaceae	QIAStool+BB vs MagNAPure
141.200238	0.93724671	0.38880135	2.41060559	0.01592606	0.09113246	Chitinophagaceae	QIAStool+BB vs MagNAPure
377.356263	0.62154676	0.27788464	2.23670791	0.02530544	0.09256462	Sphingobacteriaceae	InnuPURE vs QIAStool
33.4444509	-1.6904111	0.74230936	-2.2772326	0.02277234	0.09427748	Coriobacteriaceae	QIAStool vs MagNAPure
105.619852	-1.021215	0.44996553	-2.2695406	0.02323547	0.09430867	Nitrosomonadaceae	QIAStool vs MagNAPure
22.1599751	1.78724592	0.80863491	2.21020129	0.0270912	0.09439858	Sanguibacteraceae	PowerSoil.HMP vs QIAStool
4.81963649	-1.8045008	0.81885869	-2.2036779	0.027547	0.09439858	auto67_4W	PowerSoil.HMP vs QIAStool
50.9419349	1.03667507	0.47253405	2.1938632	0.02824525	0.09439858	Desulfobulbaceae	PowerSoil.HMP vs QIAStool
8.22875632	-1.847976	0.8213212	-2.2500041	0.02444869	0.0943991	Solibacteraceae	QIAStool vs QIAStool+BB
29.0135086	-1.1098265	0.58897604	-1.8843321	0.05952006	0.09453186	Alteromonadaceae	EasyDNA vs MagNAPure
91.6048239	-0.7732133	0.36814481	-2.1002967	0.03570275	0.09458097	Halomonadaceae	EasyDNA vs QIAStool+BB
2.3093169	-2.2044833	1.06566745	-2.0686409	0.0385798	0.09503659	Myxococcaceae	EasyDNA vs PowerSoil.HMP
642.395629	-0.7773137	0.37669477	-2.0635108	0.03906412	0.09503659	Rhodocyclaceae	EasyDNA vs PowerSoil.HMP
118.159531	-1.6497585	0.49812382	-3.3119446	0.0009265	0.09589264	Rhodobacteraceae	FastDNA vs InnuPURE
49.8040986	-1.915968	0.59985447	-3.1940546	0.0014029	0.0967998	Actinomycetaceae	PowerSoil.HMP vs QIAStool+BB
31.145957	-2.1008028	0.92480483	-2.2716174	0.02310963	0.0973037	Aerococcaceae	FastDNA vs PowerSoil.HMP
15.0781299	1.10535598	0.54633858	2.02320688	0.04305182	0.0977208	[Odoribacteraceae]	EasyDNA vs FastDNA
44.0121162	-1.4733537	0.62305494	-2.3647251	0.01804346	0.09781453	Microbacteriaceae	QIAStool+BB vs MagNAPure
1756.14884	-1.2328734	0.46055978	-2.6769019	0.00743064	0.09793244	Neisseriaceae	InnuPURE vs PowerSoil.HMP
243.261282	-0.9574556	0.36120868	-2.6506993	0.00803253	0.09793244	GZKB119	InnuPURE vs PowerSoil.HMP
44.0121162	1.80866043	0.68432745	2.64297515	0.00821811	0.09793244	Microbacteriaceae	InnuPURE vs PowerSoil.HMP
2.61442095	2.04322522	0.93447232	2.18650159	0.02877893	0.0979854	Ellin515	FastDNA vs QIAStool
10.9576795	-1.3578423	0.72891946	-1.8628152	0.06248827	0.09809204	Acholeplasmataceae	EasyDNA vs MagNAPure
593.953594	-0.6205976	0.28170633	-2.2029949	0.0275951	0.09841013	Cytophagaceae	EasyDNA vs InnuPURE
28.7345598	-1.3134923	0.59690916	-2.2004895	0.02777218	0.09841013	Parachlamydiaceae	EasyDNA vs InnuPURE
22.2584553	1.27850913	0.68878738	1.85617385	0.06342878	0.09842397	S24-7	EasyDNA vs MagNAPure
44.0121162	1.16764997	0.53725954	2.17334431	0.02975441	0.09856449	Microbacteriaceae	FastDNA vs QIAStool
3.5697885	2.04025878	0.94204076	2.1657861	0.03032754	0.09856449	Beutenbergiaceae	FastDNA vs QIAStool
38.1375375	1.28724283	0.57931343	2.22201449	0.02628233	0.09873631	Procabacteriaceae	QIAStool vs QIAStool+BB
283.625111	1.51356979	0.66399228	2.27949907	0.02263742	0.09878145	Lactobacillaceae	PowerSoil.HMP vs MagNAPure
70.3986464	0.85713572	0.41436171	2.06856886	0.03858656	0.09879332	Desulfovibrionaceae	EasyDNA vs QIAStool+BB
4.87640195	2.25110021	1.09119838	2.06296146	0.03911629	0.09879332	Corynebacteriaceae	EasyDNA vs QIAStool+BB
1.41904526	2.24885942	1.09088645	2.06149725	0.03925563	0.09879332	Dehalobacteriaceae	EasyDNA vs QIAStool+BB
4.46443875	1.94043662	0.89629733	2.16494746	0.03039171	0.09896787	Xanthobacteraceae	PowerSoil.HMP vs QIAStool
28.7248963	-1.2327152	0.56097099	-2.1974669	0.02798712	0.09899147	Acetobacteraceae	EasyDNA vs QIAStool
38.1375375	-1.3782365	0.62635739	-2.2003995	0.02777856	0.09900564	Procabacteriaceae	InnuPURE vs QIAStool
7.94601326	2.49871504	0.90484448	2.76148565	0.0057539	0.09925485	Micrococcaceae	InnuPURE vs MagNAPure
13.1651756	-1.6658237	0.76085582	-2.1894078	0.02856721	0.09927107	Helicobacteraceae	InnuPURE vs QIAStool
224.417038	-1.0205732	0.44198438	-2.3090707	0.02093966	0.09943231	Caulobacteraceae	FastDNA vs MagNAPure
1756.14884	-0.8783179	0.38202572	-2.2991067	0.02149888	0.09943231	Neisseriaceae	FastDNA vs MagNAPure
49.8040986	1.32588889	0.5667839	2.33931996	0.01931888	0.09949224	Actinomycetaceae	QIAStool+BB vs MagNAPure
1756.14884	-0.7194269	0.35366435	-2.034208	0.04193062	0.09951465	Neisseriaceae	EasyDNA vs PowerSoil.HMP
10.9576795	-1.5130076	0.74448962	-2.0322749	0.04212583	0.09951465	Acholeplasmataceae	EasyDNA vs PowerSoil.HMP
1.41904526	2.33278776	1.06856001	2.18311347	0.02902745	0.09977434	Dehalobacteriaceae	EasyDNA vs InnuPURE
4.87640195	2.35164946	1.07956479	2.17833102	0.0293814	0.09977434	Corynebacteriaceae	EasyDNA vs InnuPURE
15.0781299	1.47004964	0.68024796	2.16104968	0.0306915	0.09985342	[Odoribacteraceae]	EasyDNA vs InnuPURE
60.913589	-0.9475891	0.43964149	-2.1553677	0.03113307	0.09985342	SB-1	EasyDNA vs InnuPURE
1.54406709	-2.3845863	1.10706543	-2.1539706	0.03124248	0.09985342	Mycobacteriaceae	EasyDNA vs InnuPURE
