# Supplementary material for: Impact of Sample Type and DNA Isolation Procedure on Genomic Inference of Microbiome Composition
Source: mSystems. 2016 Oct 18;1(5):e00095-16. doi: 10.1128/mSystems.00095-16 (PMC5080404; doi:10.1128/mSystems.00095-16)
Supplement: Text S1 [file sys005162057s1.pdf]

doi:10.1128/mSystems.00095-16

SUPPLEMENTAL MATERIAL AND METHODS

Specimen Collection and Handling

Human fecal specimens were collected from a healthy individual at three time points over a single day. The specimens were kept at 4°C, and transported to the laboratory within 24 hours. Upon arrival, the three samples were pooled and homogenized. For this study, fecal specimens from an infant were chosen, as infant fecal samples often contain a high proportion of Actinobacteria (e.g. Bifidobacteria), from which genomic DNA can be difficult to isolate. Pig fecal specimens were collected from animals at a conventional pig production farm in Denmark. Samples from individual animals were obtained directly after defecation, stored in a cooling box, and transported to the laboratory within four hours. Upon arrival, three random samples were pooled and homogenized. Untreated sewage was collected from the sewage inlet of the Herlev hospital waste water treatment plant, Denmark. Specimens were stored in a cooling box and transported to the laboratory within two hours. Upon arrival 24 x 40 ml sewage samples were sedimented for 10 minutes at 8000xg in an Eppendorf 5810R centrifuge. The sewage pellets were pooled and homogenized. For all three types of specimen (human feces, pig feces, sewage), the homogenized samples were separated into 0.5 g aliquots, respectively. A subset of aliquots for each specimen type was spiked with two bacterial strains (see details below). The individual sample aliquots with and without strain mix were stored at -80°C until further processing.

Spiking with strain mix

Subsequent to specimen collection, about half of the aliquots from the human feces, pig feces, and sewage were spiked with a representative of Gram-positive and Gram-negative bacteria, namely *Staphylococcus aureus* ST398 (strain S0385) and *Salmonella enterica* serotype Typhimurium DT104. The strains were cultivated in Luria-Bertani (LB) broth at 37°C. Cells were harvested when the culture reached late exponential growth phase at OD_{600} ~0.9. The strain mix was prepared by mixing equal volumes of the bacterial cultures. To determine the number of cells of *S. aureus* ST398 and *Salmonella* Typhimurium DT104 in the two cultures, dilutions of these were plated on LB agar, the plates incubated overnight at 37°C, and colony forming units (CFU) determined the following day. The strain mix was added at about 5% of the volume of the aliquot, and the added cell numbers of *S. aureus* and *S. enterica* Thyphimurium were calculated based on the CFU determinations.

DNA isolation

In a first step, seven DNA isolation procedures were examined, namely: InnuPure® C16 (Analytic Jena AG), MagNA Pure LC DNA isolation Kit III (Roche), Easy-DNA™ gDNA Purification Kit (Invitrogen), MP FastDNA™ Spin Kit (MP Biomedicals), PowerSoil® DNA Isolation kit (MoBio), QIAamp® DNA Stool Mini Kit (Qiagen), QIAamp® DNA Stool Mini Kit (Qiagen) +Bead Beating (see Table 1, and details below). These methods were selected because they are

widely used and represent a variety of isolation procedures involving manual or automated DNA isolation, DNA separation using filter-columns or magnetic beads, chemical or mechanical lysis, and phenol/chloroform-based or non-chloroform based isolations. Bead-beating steps were performed in a Qiagen TissueLyser II if not stated otherwise, and centrifugation steps were carried out in an Ole Dich 157.MP Microcentrifuge (Denmark). DNA isolation was performed on duplicate or triplicate aliquots, dependent on specimen availability. One to two isolation controls were included at each round of isolation.

InnuPure® C16, Analytic Jena AG (InnuPURE)
Automatic isolation with the InnuPURE–C16 robot using the InnuPURE Stool DNA Kit–IP–C16 according to the manufacture's instructions. Prior to the automatic isolation, a lysis step was performed according to the protocol for lysis of bacterial DNA from stool samples using a SpeedMILL PLUS provided by the manufacturer. The cell disruption process was carried out two times for 30 sec at 50 Hz (50 s^{-1}). The DNA was eluted in 100 ul of buffer supplied with the kit.

MagNA Pure LC DNA isolation Kit III, Roche (MagNAPure)
Automatic isolation with the MagNA Pure LC instrument using the DNA Isolation Kit III (Bacteria, Fungi) according to the manufacture's instructions. The pre-isolation step for stool samples described in the protocol was performed before transferring the samples to the MagNA Pure LC. The protocol states a starting amount of a peanut-size sample, and in order to ensure consistency across isolations a starting amount of 0.25 g was chosen. The DNA was eluted in 100 ul of buffer supplied with the kit.

Easy-DNA™ gDNA Purification Kit, Invitrogen (Easy-DNA)
The DNA isolation was performed according to the manufacturer's instructions with minor modifications. Pretreatment of the samples were performed following the protocol for small amounts of cells, tissues, or plant leaves. Initially, 0.25 g sample aliquots were resuspended in 1.5 ml 0.9% NaCl, respectively. The samples were centrifuged at 600xg for 3 minutes. The supernatant was transferred to new tubes and centrifuged at 8000xg for 10 minutes. After centrifugation, the supernatant was discarded and the pellet was resuspended in 200 µl PBS. 30 µl lysozyme (10 mg/ml) and 15 µl lysostaphin (10 mg/ml) were added and the samples were incubated at 37°C for 20 minutes shaking at 550 rpm, before adding 30 µl 10% SDS. The final pretreatment step included the addition of 15 µl proteinase K (20 mg/ml) and incubation at 37°C for 20 minutes. The final step in the isolation protocol was prolonged to an incubation for 1.5 (instead of 0.5) hours at 37°C. The DNA was eluted in 100 ul of buffer supplied with the kit.

MP FastDNA™ Spin Kit, MP Biomedicals (FastDNA)
The DNA isolation was performed according to the manufacturer's instructions with minor modifications. A centrifugation step at 3000xg for 2 minutes was included to ensure proper settling of the silica matrix. The protocol suggested

eluting the DNA in 50-100 µl DNase/Pyrogen-Free water, and here the DNA was eluted in 100 µl.

PowerSoil® DNA Isolation kit, MoBio Laboratories Inc. (PowerSoil.HMP)
The DNA isolation was performed according to the protocol employed in the Human Microbiome Project (HMP Protocol # 07-001 version 12), with a minor modification to the initial protocol step. The HMP protocol states to resuspend 2 ml fecal sample in 5 ml MoBio lysis buffer. Here, we resuspended 0.5 g sample in 1.25 ml MoBio lysis buffer (i.e. same ratio). Subsequently, the samples were centrifuged according to the HMP protocol and 1 ml of supernatant transferred to a garnet bead tube containing 0.75 ml MoBio buffer. The samples were heated at 65°C for 10 minutes followed by an additional heating step at 95°C for 10 minutes. The samples were processed further according to the HMP protocol including the modification at step 12, where the centrifugation step was prolonged to 2 minutes. The DNA was eluted in 100 ul of buffer supplied with the kit.

QIAamp® DNA Stool Mini Kit, Qiagen (QIAStool)
The DNA isolation was performed according to the manufacturer's protocol for isolation of DNA from stool for pathogen detection with minor modifications. In the lysis step, the samples were first heated at 70°C for 5 minutes and subsequently at 95°C for 5 minutes. The DNA was eluted in 100 µl elution buffer.

QIAamp® DNA Stool Mini Kit, Qiagen +Bead Beating (QIAStool+BB)
The DNA isolation was performed according to the manufacturer's protocol for isolation of DNA from stool for pathogen detection with minor modifications that included a bead-beating step. Sample aliquots of 0.2 g were mixed with 1.4 ml ASL buffer, respectively, and added to Lysing matrix A bead beating tubes (MP Biomedicals) and were briefly homogenized. The samples were treated in a Qiagen TissueLyser II at 30 f/s (Hz) three times for 30 seconds, with placement of the samples on ice in between bead beating steps. Subsequently, the samples were heated at 95°C for 15 minutes. The remaining steps were carried out according to the manufacturer's recommendations, and the DNA eluted in 100 ul elution buffer.

In a second step, a variety of modifications to two Qiagen kits were examined, namely the QIAamp® DNA Stool Mini Kit (QIAStool), and QIAamp® Fast DNA Stool Mini Kit (QIAFast). The latter was released to the market during the course of this study. The main difference between the QIAStool and the QIAFast kits relies in the way inhibitor compounds are being removed. In the QIAStool kit, InhibitEX tablets are being dissolved in the samples that are adsorbing the inhibitors and together are removed via centrifugation. The QIAFast kit contains an InhibitEX buffer to remove inhibitor compounds, and no tablets are required.

QIAamp® DNA Stool Mini Kit, Qiagen and Modifications

Five different protocols based on the QIAamp® DNA Stool Mini Kit were examined. i) QIAStool: see above, ii) QIAStool+BB.LMA: QIAStool+BB procedure using Lysing matrix A tubes (see above), iii) QIAStool+BB.LMA+2Trans: QIAStool+BB procedure using Lysing matrix A tubes (see above) with modifications. To reduce the loss of sample, the double amount of supernatant was transferred to proteinase K (i.e. 400 µl instead of 200 µl). The volumes of Proteinase K, buffer AL and ethanol were doubled, respectively. Due to the increased volume, the passing of the sample through the spin columns is performed in two centrifugation steps. The DNA was washed twice before elution in 100 µl elution buffer. iv) QIAStool+PreT+BB.LMA: QIAStool+BB procedure using Lysing matrix A tubes (see above) with modifications. An increased starting sample amount was used and pre-treated. 0.5g of sample was mixed with 1.5 ml 0.9% NaCl solution. After homogenization by vortexing, the samples were centrifuged at 600 x g for 3 minutes to settle large particles. The supernatant was centrifuged at 8000 x g for 10 minutes to pellet microbial cells. The pellet was resuspended in 200 µl PBS and transferred to Lysing Matrix A bead beating tubes. v) QIAStool+PreT+BB.LMA+2Trans: QIAStool+BB procedure using Lysing matrix A tubes (see above) with modifications described in iii) and iv).

QIAamp® Fast DNA Stool Mini Kit, Qiagen and Modifications
Six different protocols (i–vi) based on the QIAamp® Fast DNA Stool Mini Kit were examined using five different bead types (ii–vi). i) QIAFast: The DNA isolation was performed according to the manufacturer's protocol for isolation of DNA from stool for pathogen detection with minor modifications. In the lysis-step, the samples were first heated at 70°C for 5 minutes and subsequently at 95°C for 5 minutes. The DNA was eluted in 100 µl elution buffer for 2 minutes. ii) QIAFast+BB.LMA+2Trans: The DNA isolation was performed according to the manufacturer's protocol for isolation of DNA from stool for pathogen detection with minor modifications that included a bead-beating step. Sample aliquots of 0.2 g were mixed with 1 ml InhibitEX buffer, respectively, and added to Lysing matrix A bead beating tubes (MP Biomedicals) and are briefly homogenized. The samples are treated in a Qiagen TissueLyser II at 30 f/s (Hz) three times for 30 seconds, with placement of the samples on ice in between bead beating steps. Subsequently, the samples are heated at 95°C for 7 minutes. Similar to the modifications described above, following the bead-beating and heating steps, the double amount of supernatant was transferred to proteinase K (i.e. 400 µl instead of 200 µl). The volumes of proteinase K, Buffer AL and ethanol were also doubled. The passing of the sample through the filter columns were subsequently carried out in two centrifugation steps rather than one, to accommodate the increased sample volume. The remaining steps were carried out according to the manufacturer's recommendations, and the DNA eluted in 100 ul elution buffer. A laboratory protocol for this procedure can be found at https://dx.doi.org/10.6084/m9.figshare.3475406. iii) QIAFast+BB.LTS+2Trans: Same procedure as described in ii) with Pathogen Lysis Tubes S (Qiagen). iv) QIAFast+BB.LTL+2Trans: Same procedure as described in ii) with Pathogen Lysis Tubes L (Qiagen). v) QIAFast+BB.GBT+2Trans: Same procedure as

doi:10.1128/mSystems.00095-16

described in ii) with Garnet Bead Tubes (MoBio). vi) QIAFast+BB.AAB+2Trans: Same procedure as described in ii) with A&A Bead Tubes (A&A Biotechnology, Gdynia, Poland).

Together, the evaluation and improvements of DNA isolation methods were carried out in a step-wise approach. In the first step, seven DNA extraction kits were evaluated using human feces, pig feces, and hospital sewage (Figures 1–4, Supplemental Figures S1-S4, and Supplemental Tables S1+S2). The standard and modified procedures based on the QIAStool and QIAFast methods were tested using a second set of pig fecal samples (Figure 5A, and Supplemental Figure S5, and Supplemental Table S3). Upon evaluation of the different DNA isolation methods, promising procedures were selected and examined using a new set of human feces, pig feces, and hospital sewage (Figure 5B).

DNA quantitation and quality assessment
Subsequent to DNA isolation, the DNA was portioned into 10 µl aliquots to prevent repeated freeze-thawing cycles, and stored at -20°C. DNA concentrations were measured using Qubit® dsDNA BR Assay Kit on a Qubit® 2.0 Fluorometer (Invitrogen, Carlsbad, CA). As DNA extracts can contain contaminants, such as proteins or other organic molecules that can affect downstream procedures such as DNA amplifications in PCR, we determined the DNA purity by measuring the ratios of absorbance at 260/280 and 260/230, respectively, using a NanoDrop 1000 Spectrophotometer (Thermo Scientific, Pittsburgh, USA). DNA extracts with a 260/280 ratio between ~1.7 to ~ 2.0, and 260/230 ration between ~2.0 to ~2.2 are regarded as "pure". The stability of the DNA in the extracts was determined by measuring the DNA concentration after 2 and 7 days incubation at 22°C. A decrease in DNA concentration over time can indicate the presence of DNases in the extract.

16S rRNA gene profiling
16S rRNA amplicon libraries were generated using a two-step protocol similar as described in Part # 15044223 Rev. B by Illumina (http://www.illumina.com/content/dam/illumina-support/documents/documentation/chemistry_documentation/16s/16s-metagenomic-library-prep-guide-15044223-b.pdf). In a first PCR, the V4 region of the 16S rRNA genes were amplified using the universal primers (515f 5'-TGCCAGCAGCCGCGGTAATAC (1) and 806r 5'-GGACTACNNGGGTATCTAAT (2). Each 20-µl PCR reaction contained 2 µl of 10 x AccuPrime PCR Buffer II (15mM $MgCl_2$, Invitrogen), 1 µl (10 µM) of the primers, 0.12 µl AccuPrime Taq DNA polymerase (2 units/µl, Invitrogen), 1 µl template DNA and 14.88 µl ddH_2O. PCR conditions: denaturation at 94°C for 2 min; 30 cycles at 94°C for 20 s, 56°C for 20 s, 68°C for 30 s; followed by 68°C for 5 min, and 3 min at 70 °C. Subsequently, the PCR products were placed on ice to prevent hybridization between PCR products and nonspecific amplicons. Samples were quantified using Quant-iT™ PicoGreen® dsDNA Assay Kit (Invitrogen, Carlsbad, CA) on a Lightcycler 96 (Roche, Mannheim, Germany) and adjusted to equal concentrations. In the second PCR the same conditions were

used as in the first round, except the PCR was reduced to 15 cycles and the primers had a unique adaptor/linker/index sequence per sample (3). The PCR products were purified using Agencourt AmPure XP beads (Beckman Coulter Inc, A63881), and concentrations were measured using Quant-iT™ PicoGreen® dsDNA Assay Kit on a Lightcycler 96. The samples were pooled in equal concentrations, and concentrated using 'DNA clean and concentrator-5 kit' (Zymo Research, Orange, CA). Paired-end 2 × 250 bp sequencing of barcoded amplicons was performed on a MiSeq machine running v2 chemistry (Illumina Inc., San Diego, CA, USA) at University of Copenhagen, Section of Microbiology.

The primer sequences were trimmed, quality filtering performed, and paired sequences assembled using the UPARSE pipeline http://drive5.com/usearch/manual/uparse_pipeline.html (4). Low quality reads were removed with a maximum expected error threshold of 0.5 (maxee). Sequences were barcoded and pooled before dereplication and removal of duplicates (-minseqlength 64). Prior to clustering of the OTUs the dereplicated reads were sorted according to abundance, and singeltons where removed (http://drive5.com/usearch/manual/upp_readprep.html). Chimera filtering was performed using UCHIME (5) with rdp_gold.fa as reference database. The reads were mapped back to OTUs, including singletons, at a 97% identity level and an OTU-table was generated using uc2otutab.py. Using QIIME1.8.0 (6), taxonomy was assigned with uclust using assign_taxonomy.py based on the Greengenes 13.8 reference database. The average number of reads per sample was 192965, and the read length was between 186-251 bp. The average number of reads in the isolation controls was 34063 and the majority of these reads were affiliated with the two strains used for spiking (Enterobacteriaceae and Staphylococcaceae), as well as dominant taxa that were present in the complex samples, such as Ruminococcaceae, Prevotellaceae, and Bacteroidales. Ecological diversity estimates and microbial community comparisons were performed using the relevant scripts provided by QIIME, phyloseq, and R (6-8). For the estimation of bacterial diversity and richness (Fig. 1C, and Fig. 5A), and principal coordinate analysis (Fig. 2A-C, and Fig. S3) the samples were rarefied to 800 reads per sample. The abundance of Gram-positive and Gram-negative bacteria was predicted at order levels based on information from the literature. For some bacteria (mainly Firmicutes), the Gram status could not be assigned at this level, and for those the family level was used instead.

Metagenomics

A subset of thirty-nine DNA extracts was subjected to metagenomic sequencing. The samples were prepared and sequenced following the Nextera XT DNA Library Preparation Guide for the MiSeq system, Part # 15031942 Rev. D (http://support.illumina.com/content/dam/illumina-support/documents/documentation/chemistry_documentation/samplepreps_nextera/nextera-xt/nextera-xt-library-prep-guide-15031942-01.pdf), using paired-end v2 2×250bp sequencing. The taxonomic microbiome compositions were determined through the use of the MGmapper pipeline (9). The MGmapper package is available for download at

www.cbs.dtu.dk/public/MGmapper/. The analysis consisted of three main steps: i) Pre-processing and quality trimming of raw reads, ii) Mapping of reads to reference sequence databases, and iii) Analysis of read count data. In the first step, cutadapt (10) was employed for adapter sequence removal, trimming of low-quality bases from the ends of the reads (-q 30), and removal of reads that were shorter than 30 bp. In a second step, the remaining paired-end reads were mapped in chain-mode to four databases: 1. complete bacterial genomes, 2. draft bacterial genomes, 3. MetaHit Assembly (http://www.sanger.ac.uk/resources/downloads/bacteria/metahit/, July 2014), and 4. Human Microbiome assemby (http://www.hmpdacc.org/resources/data_browser.php, July 2014) using the BWA-MEM algorithm (http://bio-bwa.sourceforge.net). For the analysis in the present study only the reads mapping to the two primary bacterial databases (complete and draft bacterial genomes) were considered. These two databases were composed of 2685 complete and 22224 draft bacterial and archaeal genomes obtained from Genbank on July 2014 and December 2014, respectively. The order by which the databases are specified in chain-mode is important, as reads that exhibit a significant hit to the previous reference database are removed before mapping to the next database. Samtools (10) was used to remove singletons and all reads that did not map as pairs. An alignment of a read pair with a region in a genome was considered a hit only if the sum of the alignment scores (SAS) was higher than any SAS values from other hits in the database. In the third step, the alignments were filtered based on the Fraction of Matches+Mismatches (FMM) threshold, i.e. the fraction of a read that should align. Here, the default FMM threshold of 80% was used. From 96 155 142 raw read pairs, 7 567 574 read pairs mapped genomes in the two reference databases. The final read count table was composed of 9436 bacterial and archaeal reference strains with an average of 69952 mapped read pairs per sample. For each sample, the read counts were normalized according to the genome length of the respective genomes in the database, and for sequencing depth using total sum scaling.

Differential abundance analysis

In order to test for the differential abundance of taxa that may drive the differences observed between the communities derived from the different DNA isolation procedures, we performed DESeq2 analyses. The (unnormalized) read count tables from the 16S rRNA gene profiling and metagenomics sequence analysis, respectively, were aggregated to the family level in R (v. 3.2.3, 64bit) (8). We performed an analysis that allows for varied sequencing depth, similar as suggested previously (12), and carried out two-sided Wald tests as implemented in the DESeq2 package (v. 1.10.1) (13). The size factors were determined by DESeq2 from the read count tables. An example for such an analysis is available from https://dx.doi.org/10.6084/m9.figshare.3811251 (available as .Rmd, .html, and .pdf).

When testing the effect of added strain mix, we included the samples to which the strain mix was added as well as the corresponding samples to which no strain mix was added and accounted for DNA isolation method and sample

matrix type. When testing the effects of the DNA isolation method, we analyzed the data from the three types of fecal specimen separately and extracted results from all two-wise comparisons of DNA isolation methods. For each DESeq2 test, p-values were adjusted for the false-discovery rate (FDR) using the Benjamini-Hochberg procedure (14). As recommended by DESeq2, comparisons with an FDR below 0.1 were considered significant. For the visualization of the data, the read count data were variance-stabilized using the DESeq2 regularized log (rlog) transformation. This transformation also accounts for sequencing depth differences, allowing inter-sample comparisons of taxa.

Quantification of strain mix

The samples that were spiked with the strain mix composed of *S. enterica* Typhimurium DT104 and *S. aureus* ST398 were extracted, sequenced, and analyzed together with the non-spiked samples. For each type of specimen and isolation method, the relative abundance of Enterobacteriaceae and Staphylococcaceae for 16S rRNA gene profiling and metagenomics, respectively, were determined. Our differential abundance analysis using DESeq2 confirmed, that these two strains were present in significantly higher abundance in the spiked samples than in the not spiked samples for 16S rRNA gene profiling: Enterobacteriaceae adjusted P-value 3.08^{-30} and Staphylococcaceae adjusted P-value 2.13^{-10}; and for metagenomics: Enterobacteriaceae adjusted P-value 1.74^{-77} and Staphylococcaceae adjusted P-value 1.07^{-4}. The average relative abundance values from the samples without added strain mix were subtracted from the corresponding samples to which the strain mix was added. Subsequently, the 16S rRNA gene copy numbers of the two added strains were taken into account with 5 for *S. aureus* and 7 for *S. enterica* (for 16S rRNA gene profiling). The ratios between Enterobacteriaceae and Staphylococcaceae were determined for each sample matrix and isolation method, and compared to the *S. enterica* Typhimurium DT104 / *S. aureus* ST398 ratio of CFU that were added to the original samples.

Supplemental References

1. **Yu Y**, **Lee C**, **Kim J**, **Hwang S**. 2005. Group-specific primer and probe sets to detect methanogenic communities using quantitative real-time polymerase chain reaction. Biotechnol Bioeng **89**:670–679.

2. **Sundberg C**, **Al-Soud WA**, **Larsson M**, **Alm E**, **Yekta SS**, **Svensson BH**, **Sørensen SJ**, **Karlsson A**. 2013. 454 pyrosequencing analyses of bacterial and archaeal richness in 21 full-scale biogas digesters. FEMS Microbiology Ecology **85**:612–626.

3. **Riber L**, **Poulsen PHB**, **Al-Soud WA**, **Skov Hansen LB**, **Bergmark L**, **Brejnrod A**, **Norman A**, **Hansen LH**, **Magid J**, **Sørensen SJ**. 2014. Exploring the immediate and long-term impact on bacterial communities in soil amended with animal and urban organic waste fertilizers using pyrosequencing and screening for horizontal transfer of antibiotic resistance. FEMS Microbiology Ecology **90**:206–224.

4. **Edgar RC**. 2013. UPARSE: highly accurate OTU sequences from microbial amplicon reads. Nature Methods **10**:996–998.

5. **Edgar RC**, **Haas BJ**, **Clemente JC**, **Quince C**, **Knight R**. 2011. UCHIME improves sensitivity and speed of chimera detection. Bioinformatics **27**:2194–2200.

6. **Caporaso JG**, **Kuczynski J**, **Stombaugh J**, **Bittinger K**, **Bushman FD**, **Costello EK**, **Fierer N**, **Peña AG**, **Goodrich JK**, **Gordon JI**, **Huttley GA**, **Kelley ST**, **Knights D**, **Koenig JE**, **Ley RE**, **Lozupone CA**, **McDonald D**, **Muegge BD**, **Pirrung M**, **Reeder J**, **Sevinsky JR**, **Turnbaugh PJ**, **Walters WA**, **Widmann J**, **Yatsunenko T**, **Zaneveld J**, **Knight R**. 2010. QIIME allows analysis of high-throughput community sequencing data. Nature Methods **7**:335–336.

7. **McMurdie PJ**, **Holmes S**. 2013. phyloseq: An R Package for Reproducible Interactive Analysis and Graphics of Microbiome Census Data. PLoS ONE **8**:e61217.

8. **R Development Core Team**. 2014. R: A Language and Environment for Statistical Computing. R Foundation for Statistical Computing, Vienna, Austria.

9. **Petersen TN**, **Lukjancenko O**, **Thomsen MCF**, **Sperotto MM**, **Lund O**, **Aarestrup FM**, **Sicheritz-Ponten T**. 2015. MGmapper: Reference based mapping and taxonomy annotation of metagenomic sequence reads. In preparation. Contact: tnp@cbs.dtu.dk.

10. **Martin M**. 2011. Cutadapt removes adapter sequences from high-throughput sequencing reads. EMBnetjournal **17**:10–12.

11. **Li H**, **Handsaker B**, **Wysoker A**, **Fennell T**, **Ruan J**, **Homer N**, **Marth G**, **Abecasis G**, **Durbin R**, **1000 Genome Project Data Processing Subgroup**. 2009. The Sequence Alignment/Map format and SAMtools. Bioinformatics

25:2078–2079.

12. **McMurdie PJ**, **Holmes S**. 2014. Waste Not, Want Not: Why Rarefying Microbiome Data Is Inadmissible. PLOS Computational Biology **10**:e1003531.

13. **Love MI**, **Huber W**, **Anders S**. 2014. Moderated estimation of fold change and dispersion for RNA-seq data with DESeq2. Genome Biology **15**:31.

14. **Benjamini Y**, **Hochberg Y**. 1995. Controlling the False Discovery Rate: a Practical and Powerful Approach to Multiple Testing. Journal of the Royal Statistical Society Series B (Methodological) **57**:289–300.
